# Interface Contractility between Differently Fated Cells Drives Cell Elimination and Cyst Formation

**DOI:** 10.1016/j.cub.2015.12.063

**Published:** 2016-03-07

**Authors:** Christina Bielmeier, Silvanus Alt, Vanessa Weichselberger, Marco La Fortezza, Hartmann Harz, Frank Jülicher, Guillaume Salbreux, Anne-Kathrin Classen

**Affiliations:** 1 Ludwig-Maximilians-University Munich, Faculty of Biology, Grosshadernerstrasse 2-4, 82152 Planegg-Martinsried, Germany; 2 Max Planck Institute for the Physics of Complex Systems, Nöthnitzer Strasse 38, 01187 Dresden, Germany; 3 The Francis Crick Institute, Lincoln’s Inn Fields Laboratories, 44 Lincoln’s Inn Fields, London WC2A 3LY, UK

**Keywords:** epithelium, actomyosin contractility, epithelial cyst, cell elimination, tissue patterning, physical modeling, continuum mechanics, vertex model

## Abstract

Although cellular tumor-suppression mechanisms are widely studied, little is known about mechanisms that act at the level of tissues to suppress the occurrence of aberrant cells in epithelia. We find that ectopic expression of transcription factors that specify cell fates causes abnormal epithelial cysts in *Drosophila* imaginal discs. Cysts do not form cell autonomously but result from the juxtaposition of two cell populations with divergent fates. Juxtaposition of wild-type and aberrantly specified cells induces enrichment of actomyosin at their entire shared interface, both at adherens junctions as well as along basolateral interfaces. Experimental validation of 3D vertex model simulations demonstrates that enhanced interface contractility is sufficient to explain many morphogenetic behaviors, which depend on cell cluster size. These range from cyst formation by intermediate-sized clusters to segregation of large cell populations by formation of smooth boundaries or apical constriction in small groups of cells. In addition, we find that single cells experiencing lateral interface contractility are eliminated from tissues by apoptosis. Cysts, which disrupt epithelial continuity, form when elimination of single, aberrantly specified cells fails and cells proliferate to intermediate cell cluster sizes. Thus, increased interface contractility functions as error correction mechanism eliminating single aberrant cells from tissues, but failure leads to the formation of large, potentially disease-promoting cysts. Our results provide a novel perspective on morphogenetic mechanisms, which arise from cell-fate heterogeneities within tissues and maintain or disrupt epithelial homeostasis.

## Highlights

• Different cell fates induce actomyosin contractility at shared cellular interfaces
• Our study implies cell-cluster-size-dependent consequences for tissue integrity
• Single cells are eliminated while intermediate-sized cell clusters form cysts
• Interface contractility may function in tissue homeostasis or tumor initiation

## Graphical Abstract

**Figure:**
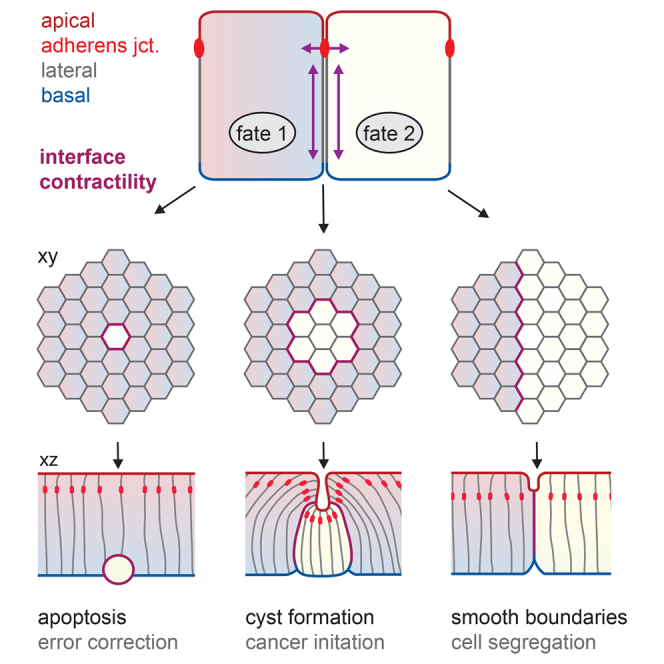

## Teaser

Bielmeier et al. demonstrate that actomyosin contractility increases at interfaces between normal and aberrantly specified epithelial cells. This drives apoptotic elimination, cyst formation, or cell segregation. These results thus provide a novel perspective on morphogenetic mechanisms arising from cell-fate heterogeneities within tissues.

## Introduction

Epithelial morphogenesis is tightly regulated to allow epithelia to act as barriers between exterior and interior environments and to fulfill functions such as protection, secretion, or absorption. Epithelial morphogenesis relies on the coordinated interplay between cell fate, cell shape, and tissue remodeling [1, 2, 3, 4]. Epithelia thus need to eliminate aberrantly specified cells to prevent disruption of tissue function or the occurrence of cancer. However, while aberrantly specified cells have been observed in tumors, little is known about how aberrant fates contribute to disruption of epithelial integrity.

In development, cell fates are specified by signaling pathways, such as Wnt/β-catenin, transforming growth factor β (TGF-β)/SMAD, Shh/Ci, or JAK/STAT. Strikingly, cells with altered Wnt/β-catenin components give rise to abnormal epithelial cysts in mouse models of colon cancer [5, 6]. Altered expression of cell-surface molecules have been suggested to drive coordinated invagination of mutant cells into cysts. In *Drosophila* imaginal discs, cell clusters mutant for Wnt/β-catenin and TGF-β/SMAD components similarly disrupt epithelial continuity through formation of cysts [7, 8, 9, 10]. In contrast to the surface-molecule-driven segregation of cell populations suggested to occur in the mouse colon, cell-autonomous reduction in mutant cell height has been implicated as direct cause of cysts in fly tissues [9, 10].

Cyst formation in *Drosophila* epithelia is not restricted to disruption of Wnt/β-catenin or TGF-β/SMAD signaling but was observed for various unrelated genetic alterations [11, 12, 13, 14, 15, 16, 17, 18, 19, 20, 21, 22, 23, 24, 25]. While cyst formation has severe consequences for epithelial function, it is not understood what cellular mechanisms drive cyst formation in these different contexts and if cyst formation is associated with a biological function. We thus sought to identify the cell-biological processes and physical forces driving cyst formation in *Drosophila* imaginal discs, which have been instrumental in elucidating mechanisms controlling epithelial architecture in development and disease. We wanted to specifically understand whether cell-autonomous shape changes [9, 10], expression of cell-surface molecules [5], coordinated apical constriction [26], or proliferation within a confined space [27] drive cyst formation to elucidate how aberrant cells disrupt epithelial integrity.

## Results

### Misexpression of Cell-Fate-Specifying Transcription Factors Underlies Cyst Formation in Imaginal Discs

**Figure 1:**
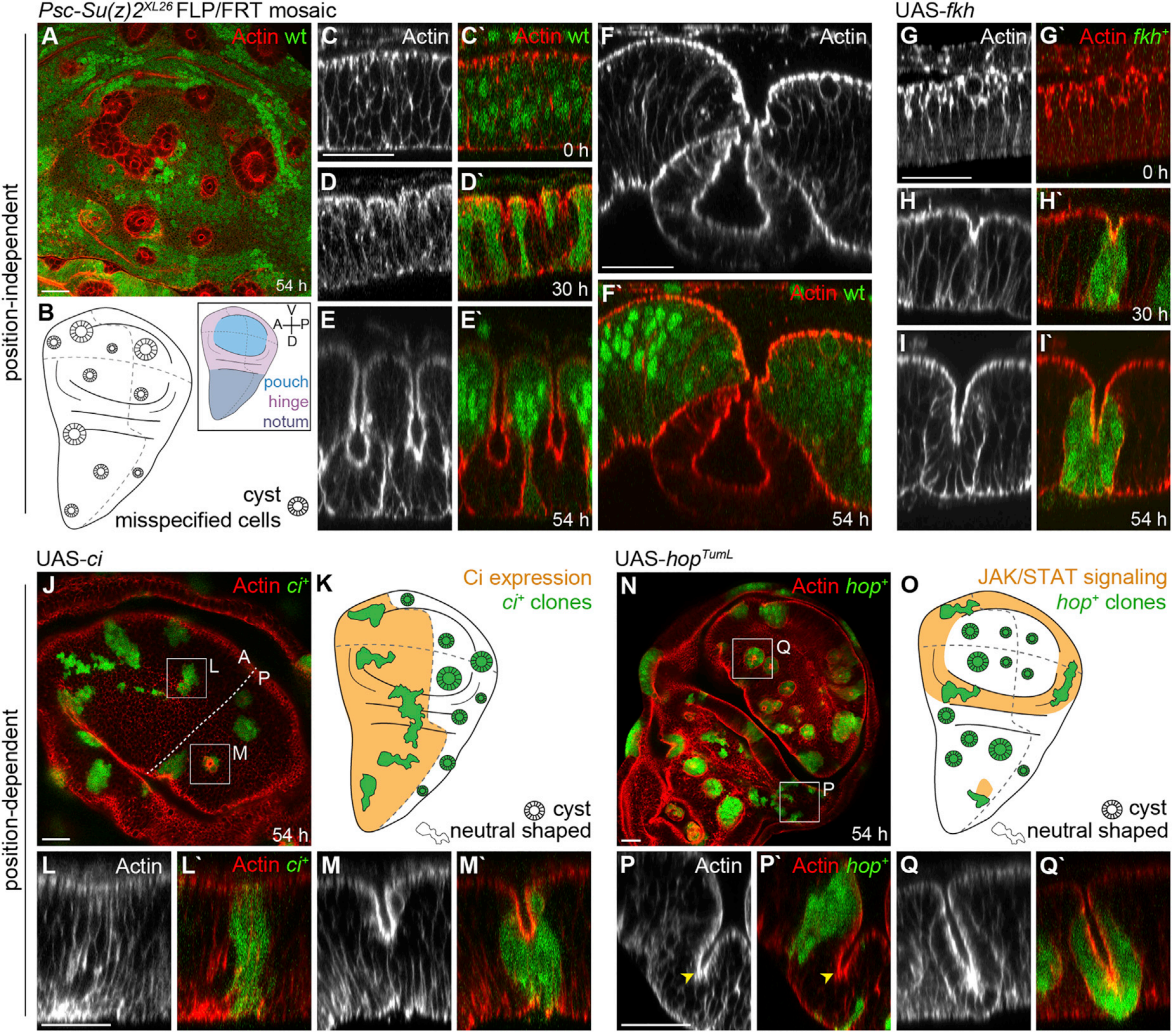
Ectopic Expression of Cell-Fate-Specifying Transcription Factors Causes Cysts (A, C–J, L–N, P–Q) Wing disc pouch containing GFP-negative, *Psc/Su(z)2*^*XL26*^ clones (A–F; GFP is shown as green in A and C–F), or GFP-positive, *fkh*-expressing (G–I), *ci*-expressing (J–M), and *hop*^*tumL*^-expressing (N–Q) clones (green in G–Q). Actin is shown in red or gray (A, C–I, J, L–N, P, and Q). Confocal xy sections at 54 hr (A, J, and N) and cross-sections (C–I, L, M, P, and Q) at 0, 30, and 54 hr after clone induction are shown. (B) Scheme of position-independent cyst formation by *Psc/Su(z)2*^*XL26*^ and *fkh*^*+*^ clones (gray). Inset defines disc subregions, compartment boundaries (dotted lines) and anterior, posterior, dorsal, and ventral axis. (K and O) Scheme of position-dependent cyst formation by *ci*-expressing (K) and *hop*^*tumL*^-expressing (O) clones in regions where Hh/Ci (K) or JAK/STAT (O) signaling (orange) is low. Boxes in (J) and (N) frame clones whose cross-sections are displayed below. Arrowhead (P) points to endogenous tissue fold. Scale bars, 25 μm. See also Figures S1 and S2.

Imaginal discs mutant for the redundantly acting, homologous tumor suppressor genes *Posterior sex combs* (*Psc*) and *Suppressor of zeste 2* (*Su(z)2*) contain epithelial cysts [21]. *Psc* and *Su(z)2* encode Polycomb proteins, which epigenetically silence cell-fate-specifying transcription factors during development [28] and restrain proliferation by repressing JAK/STAT and Notch signaling [21, 29]. FLP/FRT-induced cell clusters (“clones”) [30] homozygous for a precise deletion of both *Psc* and *Su(z)2* retracted from the apical surface of wing imaginal discs (Figures 1A–1D) and formed cyst-like structures locating to the basal side of the epithelium (Figures 1E and 1F). At late stages, many *Psc/Su(z)2* clones completely resolved contacts with wild-type cells and gave rise to persistent, proliferating cysts encapsulating an apical lumen (Movie S1).

To test whether high rates of cell proliferation were responsible for cyst initiation, we reduced proliferation in *Psc/Su(z)2* cells by interfering with the growth-promoting function of the Hippo/Yorkie pathway. We created *Psc/Su(z)2*, *yorkie* double-mutant clones and found that cysts still formed (Figures S1A–S1F). These observations strongly imply that cysts are not a result of spatial constraints imposed on proliferating *Psc/Su(z)2* cells.

In addition to restraining growth, Polycomb activity represses expression of numerous transcription factors involved in cell-fate specification [28]. To test whether fate misspecification in *Psc/Su(z)2* clones underlied cyst formation, we individually overexpressed unrelated transcription factors silenced by *Psc/Su(z)2* (Figure S1M) using the GAL4/UAS flip-out system [30]. Intriguingly, ectopic expression of the forkhead-box transcription factor *fork head* (*fkh*) involved in salivary gland morphogenesis (Figures 1G–1I and S2A–S2D) [31], the homeobox factor *Abdominal-B* (*AbdB*) involved in segment specification (Figure S2F) [32], or the Runt-domain factor *lozenge* (*lz*) required for hemocyte differentiation (see Figure S2F) [33] were each sufficient to give rise to cysts. This suggests that ectopic activation of Polycomb-silenced cell-fate-specifying transcription factors may be sufficient to drive cyst formation in *Psc/Su(z)2* clones. Accordingly, downregulation of just one derepressed transcription factor, like *fkh* or *AbdB*, in Polycomb mutant cells is insufficient to prevent cysts [12] (data not shown).

We wanted to test whether cysts are specific to transcription factors silenced by *Psc/Su(z)2*, or to cell-fate misspecification in general. We ectopically expressed randomly selected transcription factors not regulated by Psc/Su(z)2 (Figure S1M). Clones expressing the homeobox factor *ultrabithorax* (*Ubx*) (see Figure S2F) or the Pax6-homolog *eyeless* (*ey*) (Figure S2J) caused cysts in wing discs. Ectopic expression of cell-fate transcription factors caused cysts in eye discs as well (Figures S2E and S2H). In contrast, a transcription factor involved in cellular growth (*dMyc*) [34] (Figures S2N and S2N′), transcription factors dependent on co-factors for activity (*pan*, *exd*) [14, 35] (data not shown), or a cytoplasmic protein characteristic of muscle fate (Figure S2N′′), did not cause cysts. Combined, these observations suggest that aberrant activity of transcription factors specifying cell fate underlies cyst formation in imaginal discs.

Because we observed significant levels of apoptosis in clones overexpressing *fkh* (Figure S1I) or *ey* (Figure S6O), we tested whether inhibiting apoptosis could prevent cysts. However, neither co-expression of *p35* nor *dIAP1* prevented cysts (Figures S1J–S1L). Similarly, activation of JNK signaling could not account for cysts, as *Psc/Su(z)2* cysts still formed in discs mutant for the JNK-kinase *hemipterous* (*hep*) (Figures S1G and S1H). These results support that cell-fate misspecification, rather than a secondary stress response, underlies cyst formation.

The transcription factors tested caused cysts independent of clone position within the tissue (Figure 1B). Importantly, none of them are endogenously expressed in wings. In contrast, Wnt/β-catenin and TGF-β/SMAD mutant clones are reported to give rise to cysts dependent on position within endogenous signaling gradients (Figures S2P and S2Q) [7, 8, 9, 10, 25]. While Wnt/β-catenin and TGF-β/SMAD-dependent cell-autonomous shape changes were suggested to cause cysts, we wanted to test whether, instead, cell-fate misspecification in general drives cyst formation. We thus generated clones activating downstream transcriptional effectors of the conserved patterning pathways Hh/Ci (Figures 1J–1M) or JAK/STAT (Figures 1N–1Q). We found that *cubitus interruptus* (*ci*)-expressing clones maintained normal epithelial shapes in anterior compartments, where Hh/Ci signaling is high (Figures 1J–1L). However, *ci*-expressing clones formed cysts in posterior compartments, where repression of *ci* prevents Hh/Ci signaling (Figures 1J, 1K, and 1M) [36]. Likewise, expression of a dominant-active JAK (*hop*^*tum-L*^), which activates STAT [37], induced cysts only in pouch and notum regions of the disc, where JAK/STAT signaling is low (Figures 1N–1Q).

We found that misspecification within other patterning domains caused position-dependent cyst formation. Whereas *vestigial* (*vg*)-expressing clones gave rise to cysts in the hinge and notum, *homothorax* (*hth*)-expressing clones did so in the pouch. These patterns are complementary to endogenous regions of expression, which specify the proximal-distal axis of wing fates (Figures S2R and S2S). Ectopic expression of other factors, such as *Iro-C*, *salm*, and *omb*, were previously described to cause cysts in regions of low endogenous Iro-C, Spalt, and Omb activity [23, 24, 25]. Moreover, expression of the eye selector gene *eyeless* (*ey*) caused cysts in wings (Figure S2J) but rarely in eyes (Figure S2L). Collectively, these observations emphasize that cyst formation in imaginal discs represents a surprisingly general response to cell-fate misspecification and is driven by relative fate differences between misspecified and surrounding wild-type cells.

### Cyst Formation Is Driven by Non-autonomous Enrichment of Actomyosin at the Interface between Misspecified and Wild-Type Cells

**Figure 2:**
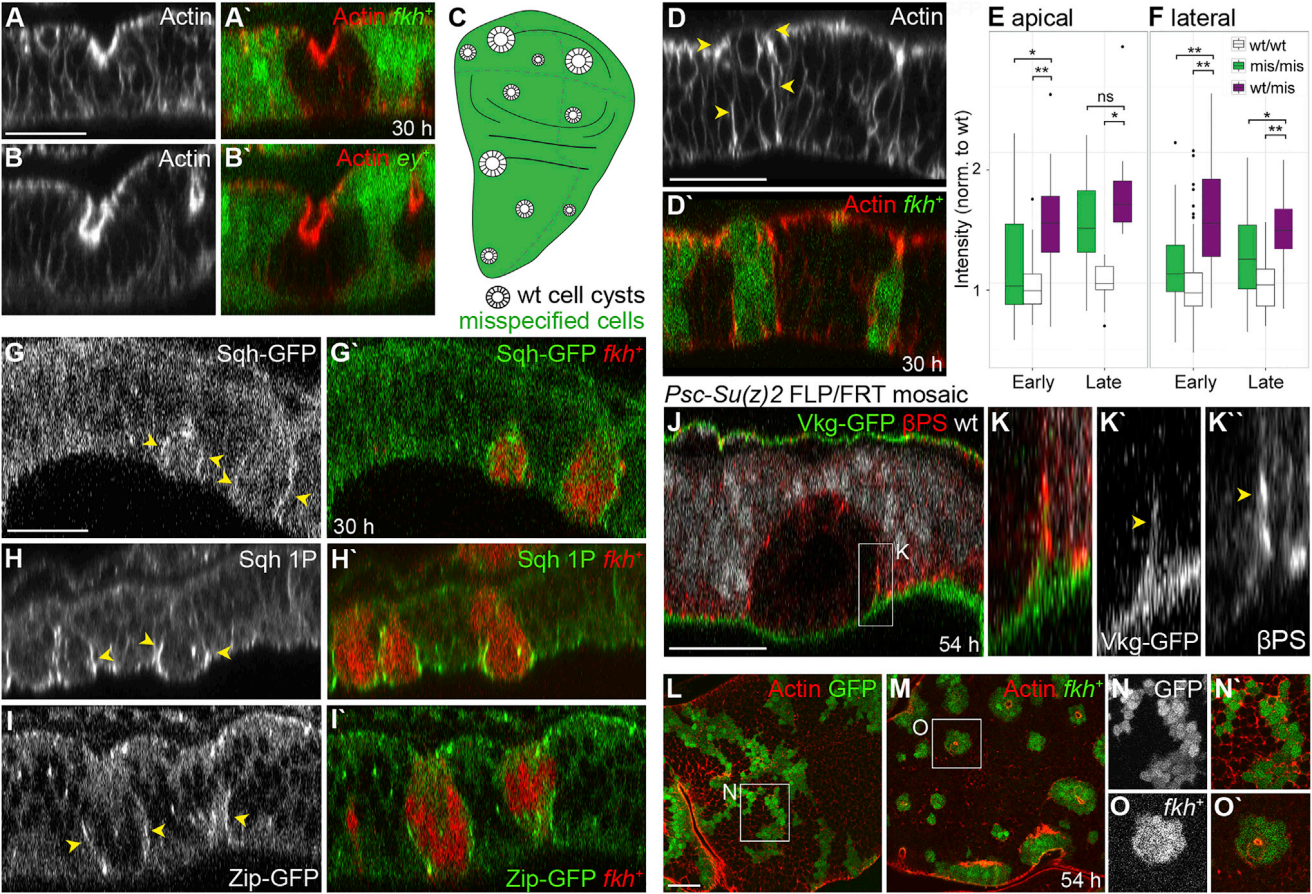
Cyst Formation Is Cell Non-autonomous and Correlates with Actomyosin Enrichment at the MWI (A and B) xz cross-sections of wild-type clones 30 hr after induction of large domains of (A) *fkh*^*+*^- or (B) *ey*^*+*^-expressing cells (green). Actin in red or gray. (C) Scheme of position-independent wild-type cysts surrounded by misspecified cells (green). (D and G–I) xz cross-section of *fkh*-expressing clones (green, D; red, G–I) 30 hr after induction in discs stained for Actin (D), expressing Sqh-GFP (G), stained for Sqh-1P (H), or expressing Zip-GFP (I) (gray or red, D; green, G–I). Arrowheads highlight enrichment at MWI. (E and F) Boxplots of normalized actin intensity at apical adherens junction (E) and basolateral interfaces (F) between wild-type (wt/wt), misspecified *fkh*^*+*^ (mis/mis), and wild-type and *fkh*^*+*^ cells (wt/mis). A two-tailed WSR test was applied. ^∗^p < 0.01, ^∗∗^p < 0.001; ns, not significant. See Figure S3R for details. (J and K) xz cross-section of *Psc/Su(z)2*^*XL26*^ clones in discs expressing CollagenIV-GFP (Vkg-GFP), stained for βPS-Integrin (βPS) 54 hr after induction. Wild-type cells are gray in (J). Arrowheads (K) point to basement membrane deformation at MWI. (L–O) xy sections of wild-type (green or gray, L and N) and *fkh*-expressing clones (green or gray, M and O) 54 hr after induction at one-third of cell height. Boxes frame regions shown at higher magnification in (K), (N), and (O). Scale bars, 25 μm. See also Figures S2 and S3.

We then asked why cysts appear in response to the presence of differently fated cells. We first wanted to understand whether cyst formation is a cell-autonomous process, reflecting altered cell shape caused by altered gene expression [9, 10]. To visualize cell-autonomous changes, we generated wing discs where the majority of cells expressed *fkh* by lengthening the heat shock, thus increasing the likelihood of neighboring cells to activate the GAL4/UAS flip-out system [30]. While *fkh*-expressing cells remained columnar, we were surprised to find that small clusters of wild-type cells in the tissue retracted from the apical surface and gave rise to cysts (Figures 2A and 2C and S2A′–S2E′). Cyst formation of wild-type clones could also be induced by overexpression of *AbdB*, *Ubx*, or *ey* (Figures 2B, S2G, S2I, S2K, and S2M). Similarly, broad expression of *ci* caused wild-type cysts, however, only in posterior compartments where Hh/Ci signaling is low (Figure S2U). Our results therefore indicate that cyst formation is not driven cell autonomously by misspecified cells, but instead by apposition of differently fated cells. Consequently, we reasoned that cysts must form by a mechanism acting at the misspecified wild-type cell interface (MWI).

To elucidate this mechanism, we analyzed cell adhesion, polarity, and cytoskeletal markers in early cysts. However, levels and localization at interfaces of *fkh*-expressing or *Psc/Su(z)2* cells were not consistently different to those at interfaces of wild-type cells (data not shown). Actin enrichment at apical surfaces of invaginating cells is likely a consequence of apical constriction, as it was seen in misspecified and wild-type cysts (compare Figure 1H with Figures 2A and 2B).

Next, we focused on interfaces between different cell fates. We found that phalloidin-labeling intensities of actin at MWI adherens junctions of *fkh*-expressing clones were increased by 30%, even if some clones had not yet undergone invagination (Figures 2D, 2E, S3N, S3P, and S3R). Importantly, we found that, in addition, actin-labeling intensities were 40% higher at basolateral MWI interfaces (Figures 2D, 2F, S3O, S3Q, and S3R). At late stages of cyst formation, enrichment at the MWI persisted (Figures 2E, 2F, and S3R). Increased actin was also detected at the MWI of *ey*-expressing clones (Figure S3S) and when wild-type cells formed cysts (Figures 2E, 2F, S2B′, S2D′, S2E′, and S3R). The resolution of a confocal microscope did not allow us to distinguish in cell-autonomous actin-labeling experiments if actin enriched at just one or both interface cortices. However, actin enrichment at the MWI, rather than cell-autonomous changes in misspecified or wild-type cells, appears to be a defining feature of early and late cyst stages.

A detailed analysis of cytoskeletal components demonstrated that the myosin II regulatory light-chain Sqh (Figures 2G, S3A, and S3B), an activated form of Sqh (Figures 2H, S3C, and S3D), the heavy-chain Zip (Figures 2I, S3E, and S3F), the FERM-domain protein Moesin (Figures S3I and S3J), and an activated form of Moesin (Figures S3K–S3M) localized to basolateral MWIs. In contrast, the myosin II regulator Rho1 was not enriched at MWI interfaces (Figures S3G and S3H). These observations demonstrate that, like actin, activated myosin and moesin are specifically recruited to the MWI and suggest that the MWI may be under increased actomyosin-mediated contractile tension.

Concurrent with enrichment in contractile components, we observed dramatic changes to the shape of the MWI. The basement membrane underwent an upward deformation accompanied by focused integrin (Figures 2J and 2K) and actin (Figures S3O and S3Q) enrichment at the MWI. These changes may reflect actin polymerization and integrin engagement as cells respond to contraction of the MWI away from the basement membrane.

While smoothening of adherens junction between differently fated cells has been described (i.e., [38]) (Figures S3E, S3N, and S3P), we observed that misspecified clones also exhibited striking minimization of basolateral contact areas (Figures 2L–2O). Basolateral clone circularity increased from 0.32 in wild-type to 0.76 in *fkh*-expressing clones (Figures S3T and S3U) and was similar to circularity of cysts formed by wild-type cells (Figure S3T).

In summary, recruitment of Actin, Myosin, and Moesin to the MWI correlated with dramatic minimization of the entire lateral contact area between wild-type and misspecified cells. This causes clones to acquire a characteristic smooth ball-like shape and culminates in complete resolution of MWI contacts, releasing *Psc/Su(z)2* cysts from the epithelium (Movie S1). We thus suggest that contractility at the MWI is indeed higher than at other cellular interfaces in the tissue.

### Changes to Mechanical Properties of the MWI Are Sufficient and Necessary to Recapitulate Cyst Formation

**Figure 3:**
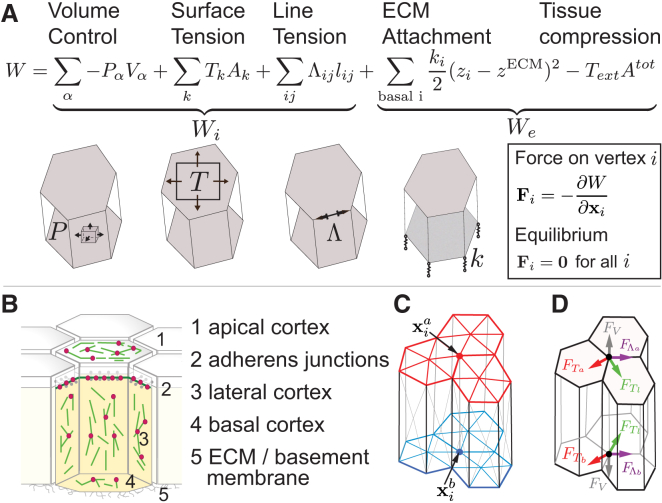
A Physical Description of Epithelia in a Three-Dimensional Vertex Model (A) Forces are obtained from an effective mechanical work function *W* that is the sum of internal and external work functions, *W*_*i*_ and *W*_*e*_. *W*_*e*_ takes into account (1) intracellular pressure *P*_*α*_ constraining cell volume, (2) surface tensions *T*_*k*_ acting on cell surfaces k, and (3) line tensions Λ_*ij*_ acting on edges between vertices i and j. *W*_*e*_ takes into account (1) springs resisting the deformation of basal vertices away from a reference plane and (2) external forces establishing compressive stress *T*_*ext*_ < 0. (B) Epithelial surface tensions arise from actomyosin cortices (actin green, myosin red) associated with apical, lateral, and basal faces. Line tensions arise from actin cables observed at adherens junctions. Extracellular matrix proteins (ECMs) cover the basal tissue surface. (C) In the model, tissue geometry is characterized by a set of vertices with positions x_i_. An additional vertex is introduced at the barycenter of each surface. Triangles connecting central and contour vertices define cell boundaries. (D) Forces acting on vertex i are obtained by differentiating the mechanical work with respect to vertex positions x_i_. Forces have contributions from surface tensions (F_T_), line tensions (F_Λ_), and cellular pressures (F_v_). See also Figure S4.

**Figure 4:**
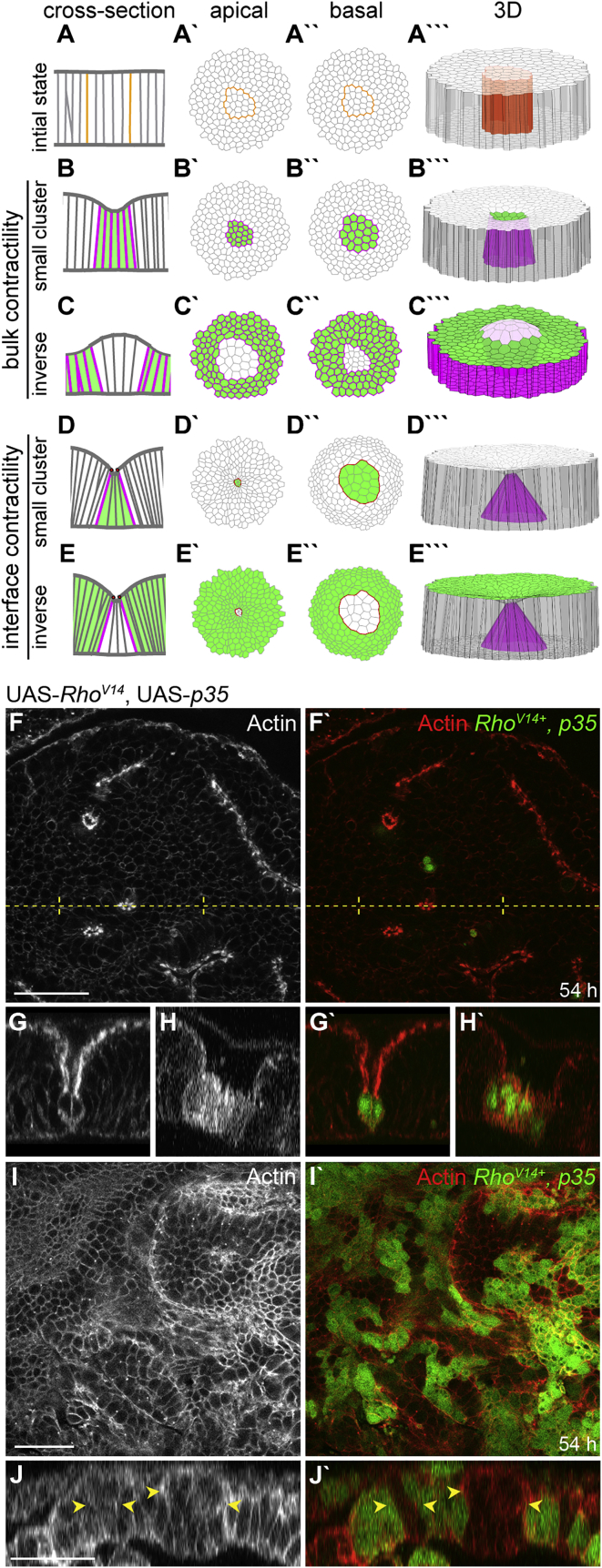
MWI Contractility Is Sufficient and Necessary to Recapitulate Cyst Formation (A–E′′′) Vertex model simulations visualize epithelial shapes in cross-section (A–E), apical (A′–E′), basal (A′′–E′′), and 3D (A′′′–E′′′) views. A clone of 20 misspecified cells is shown before (A) and after changes to mechanical properties of misspecified cells (green) (B and C, “bulk contractility”) or the MWI (D and E, “interface contractility”). Magenta and red lines represent a 3-fold increase in lateral surface and apical line tension, respectively. (F–J) xy sections (F and I) and cross-sections (G, H, and J) of *Rho*^*V14*^*,p35*-expressing cells (green) 54 hr after induction. Actin is in gray or red. Arrowheads in (J) point to interspersed wild-type clones failing to form cysts. Dotted line in (F) indicates position at which cross-section (G) was reconstructed. Scale bars, 25 μm. See also Figure S3.

To understand how mechanical properties of cells and changes in the distribution of cytoskeletal forces could drive cyst formation, we developed a three-dimensional vertex model for epithelia (Figures 3 and S4A–S4C; see “Modeling Procedures” in the Supplemental Information). We simulated the presence of a clone by placing a number of misspecified cells *N*_*c*_ within a wild-type cell population (Figure 4A) and then applied two types of mechanical changes. We modified line or surface tensions (1) in misspecified cells (“bulk contractility”; Figures 4B and 4C) or (2) only at the interface between misspecified and wild-type cells (“interface contractility”; Figures 4D and 4E).

Because reduction in cell height has been previously linked to cyst formation [7, 8, 9, 10], we first performed bulk contractility simulations where all misspecified cells experienced increased lateral surface tensions. This perturbation altered preferred aspect ratios toward cuboidal shapes and indeed caused cysts in simulations (Figure 4B). However, cyst formation by wild-type cells could not be recapitulated: wild-type cells did not invaginate but remained tall (Figure 4C).

To confirm this prediction experimentally, we expressed an activated form of the Rho1-GTPase, which caused actin to accumulate at lateral cortices and reduced the cell height (Figure 4H) [10]. As predicted by simulations, small *Rho1*^*V14*^-expressing clones caused deep indentations in discs (Figures 4F–4H). However, overexpression of *Rho1*^*V14*^ in large domains did not cause the remaining wild-type cells to invaginate (Figures 4I and 4J). Instead, both cell types exhibited different heights and, as predicted by simulations (Figures 4B and 4C), failed to minimize MWI contacts (Figures 4I, 4J, and S3T). Our results thus confirm that altering mechanical properties of individual cells can cause cysts but cannot induce cysts by wild-type cells. This suggests that cysts observed after cell-fate misspecification do not solely arise from cell-autonomous shape changes. Instead, apposition of fates induces a cellular response upstream of shape changes within misspecified cells, which drives cyst formation.

We therefore performed simulations to test whether MWI “interface contractility” is sufficient for cyst formation by misspecified and wild-type cells. We increased apical line tension and lateral surface tension at the interface and found that clones deformed into cysts (Figure 4D). As these simulations only involved changes to the MWI, inverse cysts by wild-type clones were recapitulated as well (Figure 4E). We therefore conclude that contractility at the MWI is higher than in the rest of the tissue and is sufficient and necessary to account for all cyst configurations observed in our experiments.

### Cyst Formation Is Restricted to an Intermediate Range of Clone Sizes

**Figure 5:**
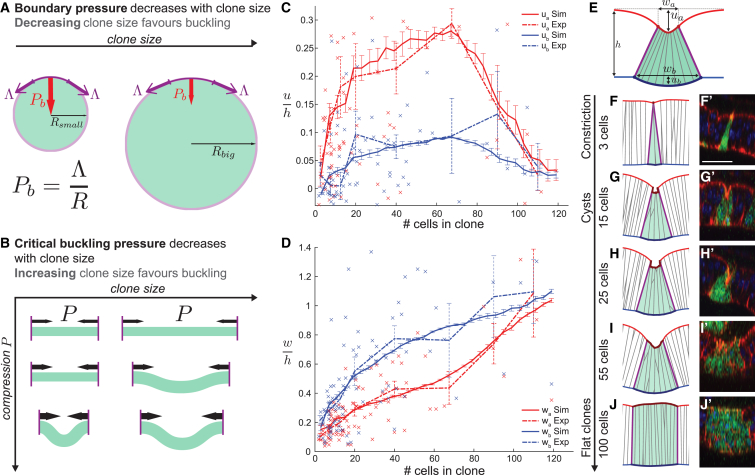
Final Clone Shape Depends on Clone Size (A) Laplace’s Law (P_b_ = Λ/R) predicts that the pressure P_b_ exerted by a contractile boundary with line tension Λ depends on the radius R of the enclosed material. Thus, large clones feel less pressure from a contractile boundary and are less likely to buckle. (B) The resistance to bending of an elastic disk depends on its radius. Smaller clones exhibit higher resistance to buckling than larger clones. (C and D) Experimental (dotted) and simulated (continuous line) deformations of apical (red) or basal (blue) cyst surfaces with respect to clone size. Parameters u_a,_ u_b_ (C) and w_a_ and w_b_ (D) are illustrated in (E). Error bars represent mean and SEM of 85 *fkh*-expressing clones 30 hr after induction and 15 simulations per data point. (E) Deformation parameters measured experimentally and fitted by simulations. w_a_, apical clone width; w_b_, basal clone width; u_a_, apical surface indentation; u_b_, basal surface deformation. (F–J) Simulated and experimental cross-sections of clones containing different cell numbers. Apical constriction, cyst formation, or minimal deformations correlate with clone size. Note that cross-section choice results in junctions not spanning apico-basal axis. Scale bars, 25 μm. See also Figures S4 and S5.

We noticed that interface simulations predicted a strong dependency of final clone shape on clone size. To better analyze this dependency, we turned to a continuum theory of tissue mechanics, which includes only a few key parameters allowing us to draw generic conclusions on tissue shape stability. On large spatial scales, the vertex model epithelium effectively behaves as a continuous elastic sheet (Figure S4H; see “Modeling Procedures” in the Supplemental Information). Elastic sheets buckle if compressed, potentially driving cyst formation in our vertex model simulations. The threshold of buckling is determined by two considerations: First, for a circular contractile boundary, the compression felt by the enclosed elastic sheet depends on the inverse radius of the boundary, as described by the law of Laplace (Figure 5A). Therefore, large clones feel less pressure from the boundary and are less likely to buckle. Second, the resistance of an elastic sheet to bending is higher on small length scales (Figure 5B). Small clones therefore have a higher resistance to buckling than larger clones. The combination of these two effects implies that very small and very large clones do not form cysts. Indeed, we find that a circular elastic sheet under tension, connected to an external extracellular matrix (ECM) and subjected to a contractile circular boundary only buckles for intermediate size ranges (Figures S4K and S4L).

To confirm this prediction experimentally, we carried out a quantitative analysis of *fkh*-expressing clone shapes as a function of clone size. We measured apical and basal clone width w_a_ and w_b_, as well as apical and basal deformation, u_a_ and u_b_ (Figure 5E). This analysis revealed that apical and basal deformations are maximal for intermediate clone sizes (N_c_ ∼70 cells), and minimal for either small or very large clones (Figures 5C and 5F′–5J′). Similarly, differences in apical and basal widths were maximal for intermediate clone sizes, corresponding to strongly wedge-shaped cysts (Figures 5D and 5F′–5J′). Very small and very large clones do not undergo strong deformations but still experience a decrease in MWI roughness (Figures 5F′, 5J′, and S3O). These quantitative results align with the predicted size-dependent impact of a contractile interface on final clone shape in simulations and further support the notion that a contractile MWI underlies cyst formation.

### A 3-Fold Simulated Increase in MWI-Tension Recapitulates Shape Parameters of Experimental Cysts

Having established experimentally for which clone sizes maximal deformations occur, we performed 3D vertex model simulations (Figures 5F–5J) to determine what range of physical forces could explain the observed relationship between clone shapes and clone size. We first established a set of mechanical parameters that reproduced experimentally measured cell aspect ratios in wild-type discs (Figure S4F; see “Modeling Procedures” in the Supplemental Information), including a parameter for ECM-induced compression [39, 40] set by measured aspect ratio changes after collagenase treatment (Figures S4D–S4G).

We then simulated cysts and measured shape parameters as for experimental clones (Figures 5C–5E). To match experimental measurements, we adjusted (1) apical, lateral, and basal surface tension of the tissue, (2) apical and basal line tension, (3) overall tissue compression, (4) stiffness of tissue-ECM attachment, and (5) apical line and lateral surface tensions at clone boundaries (Figure S5; see “Modeling Procedures” in the Supplemental Information). In doing so, we searched for the smallest increase in MWI contractility that could account for experimental measurements. We found that a 3-fold increase in apical line and lateral surface tensions at the MWI can recapitulate all features of the four experimental curves for w_a_, w_b_, u_a_, and u_b_ (Figures 5C and 5D). Importantly, an increase in apical line or lateral surface tensions alone did not recapitulate experimental observations (Figures S5E–S5G), emphasizing the functional significance of actomyosin enrichment at both adherens junctions and basolateral interfaces (Figures 2E and 2F). Furthermore, bulk contractility simulations resulted in clone shapes that did not correspond to those observed in experiments (Figure S5B), reinforcing our conclusion that bulk contractility cannot account for all phenotypes.

We then tested whether a buckling instability can indeed account for tissue deformations predicted by the 3D vertex model. We calculated estimates of the coarse-grained elastic modulus, surface tension, and bending modulus of a tissue represented by the vertex model, with line and surface tensions obtained by the fitting procedure described above (Figures S4H–S4J; see “Modeling Procedures” in the Supplemental Information). Using these parameters, we found that a buckling instability is predicted for clones below ∼106 cells (Figures S4K and S4L), in qualitative agreement with the size range of experimental and vertex model cysts (Figures 5C–5J). Therefore, a buckling transition captures the maximum clone size below which cyst formation occurs.

### Single Misspecified Cells Are Eliminated from Epithelia by MWI Contractility

In simulations and experiments, very small clones do not form cysts but display significantly reduced apical areas (Figure 5F). This resembles initial stages of cell extrusion events occurring during clearing of apoptotic or live cells [41]. It suggested that MWI contractility may specifically drive elimination of small misspecified cell clusters by promoting apical constriction and extrusion.

**Figure 6:**
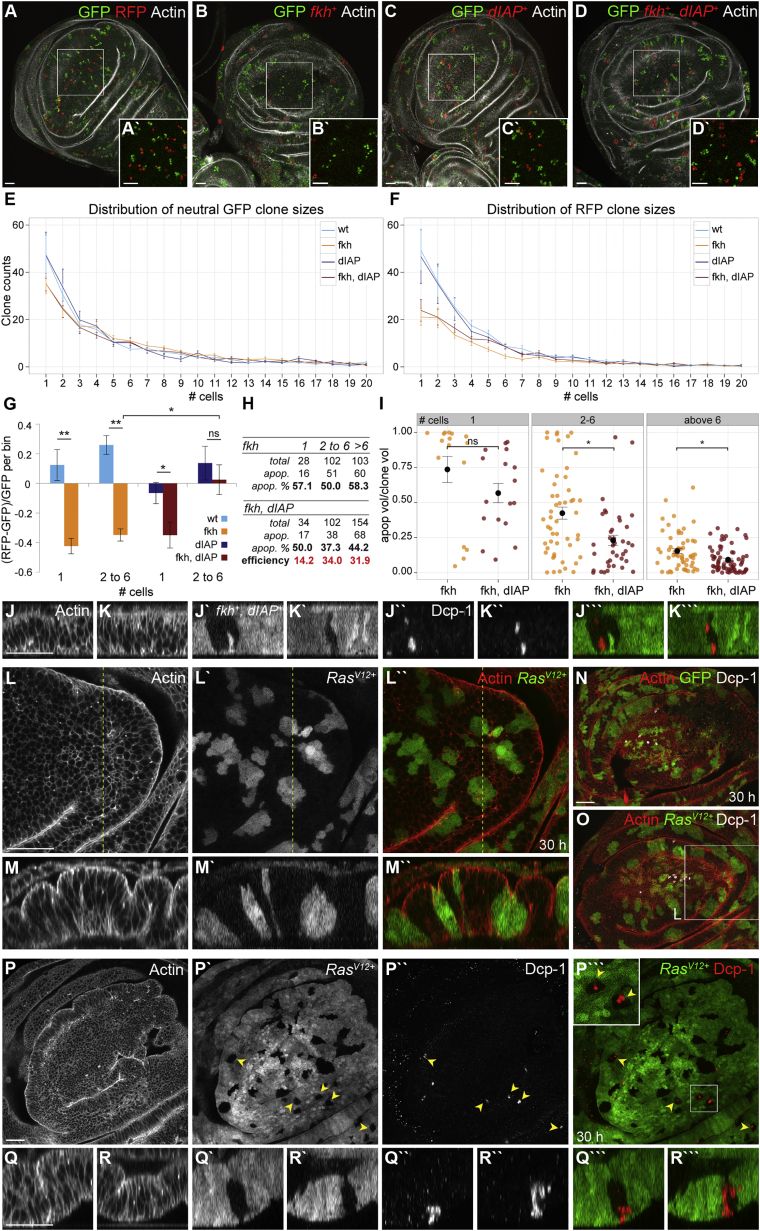
Small Misspecified Clusters Are Eliminated from Epithelia by MWI Contractility (A–D) Tie-Dye discs 30 hr after induction carrying neutral GFP-expressing (green) and RFP-expressing clones (A) or *fkh*^*+*^- (B), *dIAP1*^*+*^- (C), and *fkh,dIAP1*^*+*^-expressing clones (D) (red; Actin in gray). Boxes frame position of higher magnification insets. (E and F) Neutral GFP (E) and transgene-expressing RFP (F) clone size frequencies 30 hr after induction. RFP^*+*^ clones express either RFP alone or *fkh*^*+*^ and/or *dIAP1*^*+*^, as indicated. Histograms display clone counts for each clone size, binned into single-cell steps. (G) Relative loss of *fkh*^*+*^ and *fkh,dIAP1*^*+*^-expressing clones compared to wild-type or *dIAP1*^*+*^ control clones. For each disc, GFP^*+*^ clone counts were subtracted from RFP^*+*^ clone counts per size bin and normalized to GFP^*+*^ clone counts for the respective bin. (E–G) Mean and SEM of n = 8–10 discs for each genotype, analyzed by one-tailed WMW or Welch’s t tests are shown. ^∗^p < 0.01, ^∗∗^p < 0.001, ns, not significant. See Figures S6A and S6D for details. (H) Counts of *fkh*^*+*^- or *fkh,dIAP1*^*+*^-expressing apoptotic clones binned into three size categories: one cell, two to six cells, and above six cells. Total counts, apoptotic counts, and percentages of apoptotic clones per size bin are shown. Efficiency of inhibiting apoptosis by *dIAP1* expression was calculated as percentage of apoptosis in *fkh*^*+*^ (n = 3 discs, 233 clones) / percentage of apoptosis in *fkh,dIAP1*^*+*^ clones (n = 3 discs, 290 clones) per bin size. (I) Dot plot of Dcp-1-positive volume fractions in *fkh*^*+*^- and *fkh,dIAP1*^*+*^-expressing apoptotic clones binned into indicated size classes. Mean and SEM within bins analyzed by two-tailed WMW tests are shown. ^∗^p < 0.01; ns, not significant. See Figure S6E for details. (J–K′′′) Cross-sections of *fkh,dIAP1*^*+*^-expressing cells (J′ and K′; green, J′′′ and K′′′) 30 hr after induction. Actin (J and K), Dcp-1 (J′′ and K′′; red, J′′′ and K′′′). (L–M′′) *Ras*^*V12*^-expressing clones (L′ and M′; green, L′′ and M′′) 30 hr after induction, stained for Actin (L and M; red, L′′ and M′′). Line indicates position at which xz cross-section (M) was reconstructed. (N and O) GFP- (N) or *Ras*^*V12*^-expressing clones (O) (green) stained for Actin (red) and Dcp-1 (gray). (P–R′′′) xy sections (P) and xz cross-section (Q and R) of *Ras*^*V12*^-expressing cells (P′–R′; green, P′′′–R′′′) 30 hr after induction stained for Actin (P–R) and Dcp-1 (P′′–R′′; red, P′′′–R′′′). Box frames higher magnification inset. Arrowheads point to apoptotic wild-type clones. Scale bars, 25 μm. See also Figure S6.

To understand whether size-dependent elimination of misspecified clusters indeed occurred, we quantified the frequencies of misspecified clone sizes and compared them to those of wild-type clones. To control for experimental variability, we used the Tie-Dye technique [18] to generate misspecified cells marked by RFP and wild-type cells marked by GFP in the same disc (Figures 6B–6D). To normalize for differences in frequencies of GFP and RFP clone induction, we compared clone counts to those of neutral control discs (Figure 6A). When we analyzed Tie-Dye discs, we found that *fkh*-expressing clones of up to six cells were indeed dramatically underrepresented (Figures 6E–6G and S6A–S6D). Similarly, we observed dramatic loss of *ci*-expressing clones in the posterior compartment of Tie-Dye discs (Figures S6F–S6I).

We wanted to understand how these cells are eliminated. Because misspecified cells often underwent apoptosis (Figures S1I and S6O), we asked whether small *fkh*-expressing clones exhibited higher levels of apoptosis than larger clones. We quantified the volume occupied by apoptotic cells positive for cleaved Caspase Dcp-1 in *fkh*-expressing clones (Figure S6K). While the relative number of apoptotic clones did not change between small and large clone size bins (Figure 6H), we found that the number of apoptotic cells was significantly increased in clones containing up to six cells compared to clones larger than six cells (Figure 6I). This suggests that small *fkh*-expressing clones may be subject to stronger apoptotic stimuli than larger clones.

To understand whether apoptosis is necessary for elimination of small clones, we inhibited apoptosis by co-expressing *dIAP1* and analyzed *fkh,dIAP1*-expressing clone sizes (Figures 6E–6G and S6A–S6D) and Dcp-1 volumes (Figures 6H, 6I, S6E, S6J, and S6L). Expression of *dIAP1* rescued large *fkh*-expressing clone sizes back to wild-type sizes indicating that apoptosis in large clones is strongly reduced (Figures 6G and S6A–S6D). In contrast, *dIAP1* expression was not able to prevent loss of single *fkh*-expressing cells (Figures 6G and S6A–S6D). In addition, *dIAP1* expression reduced the number of large apoptotic *fkh*-expressing clones more efficiently than of single-cell clones (Figure 6H). Similarly, while dIAP1 reduced the number of apoptotic cells in large *fkh*-expressing clones, it did not alter relative apoptotic volumes in single cells (Figures 6I and S6E). Combined, these experiments suggest that specifically small *fkh*-expressing clones experience strong apoptotic stimuli, which cannot be counteracted by co-expression of rate-limiting levels of dIAP1, and ultimately drive elimination of small clones.

We hypothesized that if apoptosis in small clones is specific to MWI contractility, then apoptosis must also be induced in small wild-type clones encircled by MWI contractility. We thus examined wild-type clones after induction of large *fkh* or *ey*-expressing domains. We observed indeed frequent Dcp-1 activation in small wild-type cysts (Figures 6J, 6K, S6M, S6N, and S6P). Combined, our results strongly suggest that MWI contractility may drive cell elimination by inducing apoptosis specifically in small MWI-encircled cell clusters.

We next investigated a potential relevance of our observations to disruption of epithelial integrity in cancer. Specifically, we wanted to understand whether the occurrence of round clones in discs upon overexpression of oncogenic *Ras* (*Ras*^*V12*^) [16] is driven by MWI contractility. Indeed, when we visualized small *Ras*^*V12*^-expressing clones, we found that they formed cysts (Figures 6L, 6M, and 6O) [22]. Excitingly, we found that wild-type clones surrounded by *Ras*^*V12*^-expressing cells undergo interface smoothening and cyst formation (Figures 6P–6R). This suggests that oncogenic Ras induces MWI contractility, likely because Ras also specifies cell fate. While we rarely observed apoptosis in wild-type or *Ras*^*V12*^-transformed cells (Figures 6N and 6O), we found that apoptosis is frequently activated in small wild-type clones surrounded by *Ras*^*V12*^-expressing cells (Figures 6P–6R) where *Ras*^*V12*^-induced MWI-effects are strongest. Combined, these results reinforce our conclusion that MWI contractility is induced by apposition of cell populations with different fates and that MWI contractility drives cell elimination by activation of apoptosis in small, encircled cell clusters.

## Discussion

**Figure 7:**
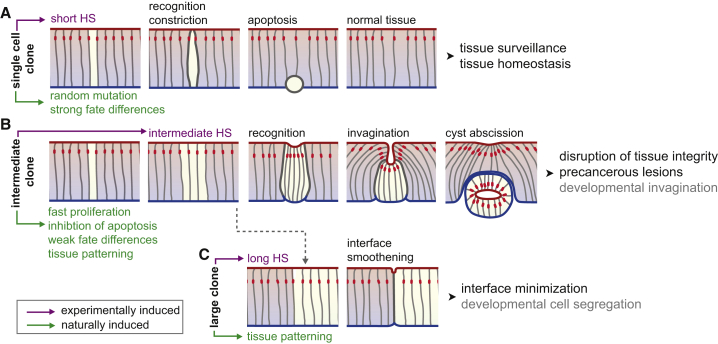
Morphogenetic Behaviors Induced by Interface Contractility Tissue with apical (red), basal (blue), lateral surfaces (gray), and adherens junctions (red). Magenta indicates experimentally induced conditions (HS, heat shock), green potentially natural scenarios creating differently fated clone sizes. Gray indicates speculation on a role of interface contractility in development. (A) Single misspecified cells are experimentally induced by a short heat shock (HS). Random mutations arise naturally in single cells and may cause fate differences. Interface contractility causes apical constriction and apoptosis to preserve tissue homeostasis. (B) Intermediate-sized clones are induced experimentally by intermediate HS. Misspecified cell clusters may arise naturally from single cells that proliferate before detection or escape apoptosis by potent onco- or tumor-suppressor-gene mutations. During development, intermediate clusters arise by patterning. Cysts compromise tissue integrity and potentially promote precancerous lesions. (C) Large clones are induced experimentally by long HS. During development, large lineage domains arise by patterning and tissue growth. Interface contractility leads to interface smoothening as observed at lineage boundaries.

We describe here the biological and mechanical effects of a cellular mechanism acting between differently fated epithelial cells (Figure 7). We find that actomyosin recruitment to an interface between different fates promotes extrusion of single cells (Figure 7A). In contrast, interface contractility around intermediate-sized cell clusters induces apical buckling into cysts (Figure 7B). In large clones, interface contractility solely decreases interface roughness (Figure 7C). We thus suggest that interface contractility acts as surveillance mechanism on single misspecified cells but also drives disease-promoting disruption of epithelial integrity by deforming cell clusters into cysts. We speculate that interface contractility may have broader functions, such as establishing compartment boundaries between different cell populations during development.

Our data suggest that MWI contractility causes cell elimination by triggering apoptosis. The signal that activates apoptosis in small clones may arise from strong apical constriction. Apical constriction may limit apical area and thus receptors available for survival signals or may regulate mechano-sensitive survival pathways such as Hippo/Yorkie [42]. Another interface-dependent process called cell competition [43], which kills metabolically unfit “loser” cells that touch fitter “winner” cells, is unlikely to play a role. Cell competition fails to elicit contractile changes at winner-loser cells interfaces and, importantly, acts unidirectional by eliminating loser cells, even if encircled by winner cells. We suggest that epithelia likely evolved multiple mechanisms to eliminate homeostatic perturbations in either metabolic activity (cell competition) or cell fate (MWI contractility).

Paradoxically, cysts are caused by a failure to eliminate intermediate-sized cell clusters. In tissues, intermediate-sized clusters may arise by proliferation of aberrant apoptosis-resistant cells. Prominently, MWI-contractility-inducing tumor suppressor mutations, such as *Psc/Su(z)2*, or transformation with oncogenic Ras^V12^ may confer apoptosis resistance. Strikingly, in mouse models of colon cancer, cysts have been observed upon deregulation of Wnt/β-catenin or TGF-β/SMAD signaling [5, 6, 44]. Our work demonstrates that disruption of many patterning fields causes cysts, emphasizing that cysts may be an early hallmark of epithelial cancers driven by mutagenic changes to cell fate. Because cysts survive abscission from tissues, their formation may promote displacement of cells into new microenvironments and may precede emergence of invasive behaviors.

In our study, many transcription factors induced MWI contractility. Previous publications have described cysts in mosaic analysis of fate specification in imaginal discs and even neuroepithelia [11, 12, 13, 14, 15, 16, 17, 18, 19, 20, 21, 22, 23, 24, 25]. We suggest that cells likely detect cell-surface cues to compare fates. Given the diversity of fates we investigated, cells must likely use multiple proteins to reference identity and regulate contractility at MWIs. Ephrin signaling [45], LRR transmembrane proteins like Capricious and Tartan [46], or Toll-receptor patterning [47] may be potential mediators of fate recognition.

Representing the tissue as an elastic sheet in a continuum theory allowed us to identify the mechanical principles driving cyst formation based on two simple physical effects that control tissue buckling instability: the law of Laplace and the resistance of tissues to bending. These findings were confirmed by a novel vertex model of epithelia, which allowed us to simulate detailed three-dimensional cellular structures. Previous studies highlighted the importance of line tension for interface morphology [4]. However, cellular forces associated with interface mechanics in three dimensions have not been explored. We show that in addition to adherens junctions, contractility at basolateral interfaces is extensively regulated. Taking this third dimension of cellular forces into account has crucial consequences for our understanding of 3D-tissue morphology, as it induces a repertoire of tissue deformation including cell extrusion, invagination, and interface smoothening. Our simulations suggest that a 3-fold increase in lateral surface tension and in apical line tension is required to account for these deformations. This increase is similar to the 2.5-fold increase in line tensions that has been estimated to act at the interface between developmentally specified compartments [48]. It will thus be crucial to investigate whether cellular mechanisms that regulate interface contractility at aberrantly specified cells and at developmental compartments are alike [4, 48]. Similarly, many developmental invagination processes are driven by cell-fate specification of intermediate-sized cell clusters [36, 49]. These processes therefore offer an opportunity to understand similarities and differences between morphogenetic behaviors driven by apposition of differently fated cells in development or disease.

## Experimental Procedures

### Fly Genetics

For detailed genotypes and experimental conditions, please refer to Table S1 and Supplemental Experimental Procedures. Briefly, FLP/FRT and “GAL4/UAS flip-out” crosses were raised at 25°C and flipase expression was induced 72 hr after egg lay (AEL) by a heat shock at 37°C. Tissues were analyzed at indicated time points after heat shock.

### Immunohistochemistry and Imaging Processing

Discs were dissected and fixed in 4% formaldehyde/PBS for 18 min. Washes were performed in PBS + 0.1 Triton X-100 (PBT) and blocking in PBT+5% normal goat serum. Discs were incubated with primary antibodies (Supplemental Experimental Procedures) overnight at 4°C. Secondary antibodies were incubated for 2 hr at room temperature. Samples were imaged using a Leica TCS-SP5 confocal microscope. Images were processed and analyzed using workflows established in Fiji (Supplemental Experimental Procedures).

## Author Contributions

C.B., S.A., G.S., and A.-K.C. designed the experiments; C.B., S.A., V.W., and H.H. performed the experiments; C.B., S.A., V.W., M.L.F., G.S., and A.-K.C. analyzed the data; S.A., F.J., and G.S. designed the 3D vertex model; and C.B., S.A., G.S., and A.-K.C. wrote the manuscript.

## Supplemental Information

Document S1. Supplemental Experimental Procedures, Modeling Procedures, Figures S1–S6, and Table S1

Movie S1. Cyst Abscission at Late Stages of Cyst Formation Wing imaginal disc containing *Psc-Su(z)2*^*XL26*^ clones at 102 hr after clone induction. Wild-type cells are marked by RFP (red) and cell outlines by phalloidin (green). A confocal xy stack was reconstructed into an xz cross-section movie.

Document S2. Article plus Supplemental Information
