# Supplementary material for: Interface Contractility between Differently Fated Cells Drives Cell Elimination and Cyst Formation
Source: Curr Biol. 2016 Mar 7;26(5):563–74. doi: 10.1016/j.cub.2015.12.063 (PMC5282066; doi:10.1016/j.cub.2015.12.063)
Supplement: Document S1. Supplemental Experimental Procedures, Modeling Procedures, Figures S1–S6, and Table S1 [file mmc1.pdf]

Current Biology, Volume *26*

Supplemental Information

Interface Contractility between Differently Fated Cells Drives Cell Elimination and Cyst Formation

Christina Bielmeier, Silvanus Alt, Vanessa Weichselberger, Marco La Fortezza, Hartmann Harz, Frank Jülicher, Guillaume Salbreux, and Anne-Kathrin Classen

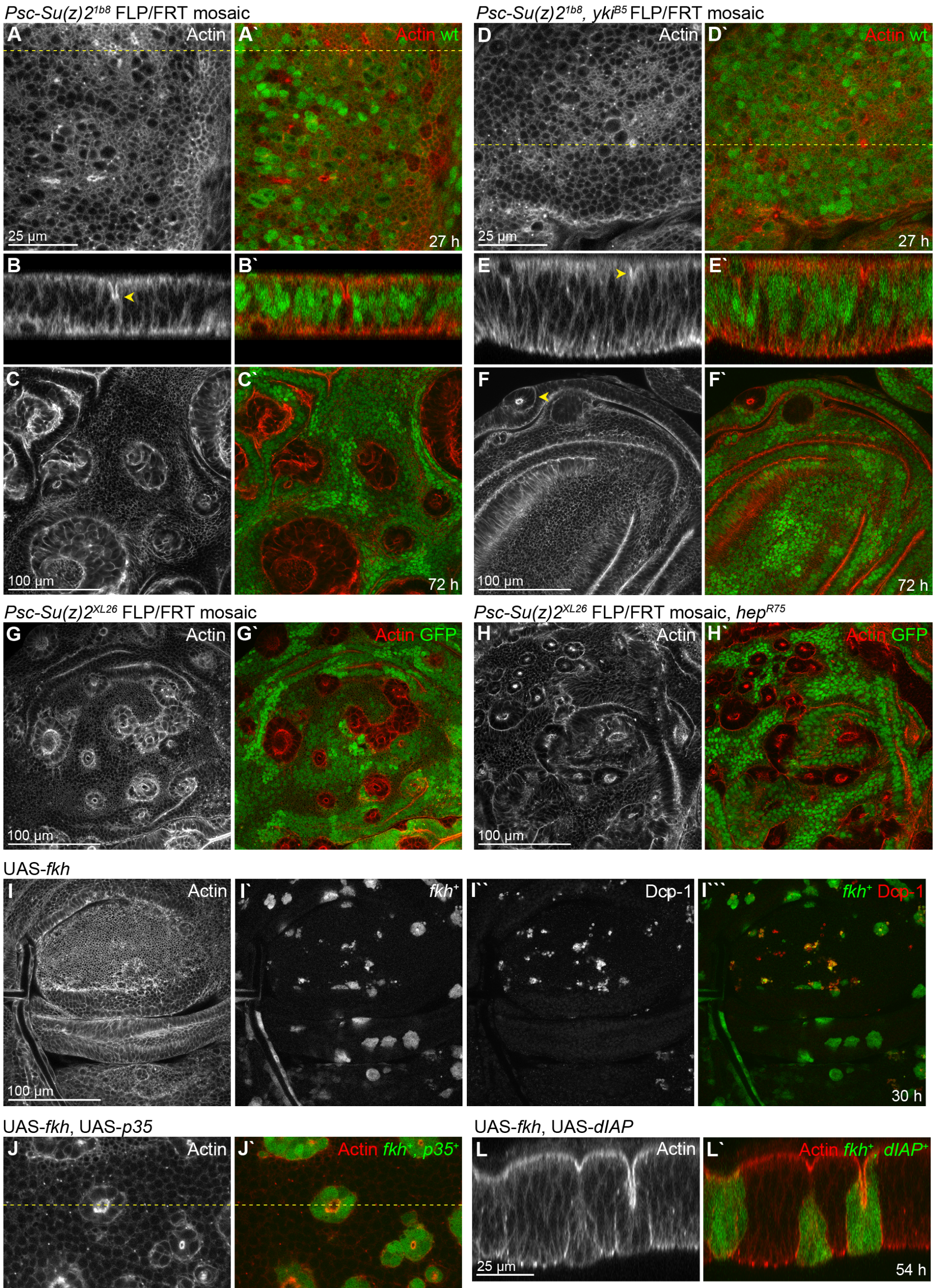

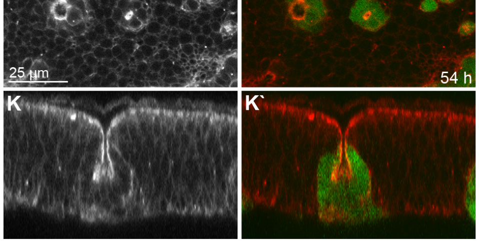

M RNA seq on *Psc-Su(z)2* mutant discs

id		foldChange	padj
FBgn0000015	Abd-B	444.96	1.4E-19
FBgn0002576	lz	123.74	1.1E-21
FBgn0000659	fkh	69.60	1.5E-11
FBgn0005558	ey	2.46	3.7E-01
FBgn0004859	ci	1.68	1.7E-03
FBgn0003944	Ubx	0.85	9.9E-01
FBgn0000117	arm	0.80	3.2E-01
FBgn0004864	hop	0.78	2.0E-01
FBgn0003716	tkv	0.27	1.1E-16

Figure S1

Overgrowth, JNK-signaling or induction of apoptosis cannot account for cyst formation by misspecified cells

Related to Figure 1

(A-F) Wing imaginal disc containing *Psc-Su(z)2*^{1b8} clones (A-C) or clones mutant for both *Psc-Su(z)2*^{1b8} and *yki*^{B5} (D-F). Wild type cells (wt) are marked by GFP (green in (A'-F')). Actin is shown in red (A'-F') or grey (A-F). Confocal xy-section at 30 h (A, D) and reconstructed xz cross-sections (B, E) at position indicated by dotted line in (A, D). Arrowheads are pointing to invaginating clones. (C, F) Confocal xy-section at 72 h after clone induction. *Psc-Su(z)2*^{1b8} mutant clones in (C) undergo extensive proliferation leading to big cysts at 72 h after clone induction. The *yki*^{B5} allele prevents proliferation and survival of *Psc-Su(z)2*^{1b8} mutant cells (F) but not invagination and cyst formation (arrowheads in E, F).

(G, H) *Psc-Su(z)2*^{XL26} clones in a wild type wing disc (G) and in a wing disc hemizygous for a *hep*^{R75} allele (H). Confocal xy-sections at 54 h after clone induction are shown. *Psc -Su(z)2*^{XL26} mutant cells are marked by lack of GFP (green) (G', H'). Actin is shown in red (G',H') or grey (G,H). *hep*^{R75} prevents activation of JNK signaling in imaginal discs but does not interfere with cyst formation.

(I) Pouch region containing *fkh*-expressing clones (grey in **I**', green in **I**''') at 30 h after clone induction showing extensive activation of apoptosis, as revealed by the presence of the cleaved effector Caspase, Death Caspase-1 (Dcp-1) (grey in **I**'', red in **I**'''). Actin is shown in (**I**).

(J-L) Pouch region containing *fkh*-expressing clones (green in J'-M') that also express the anti-apoptotic factors *p35* (J, K) or *dIAP1* (L) at 54 h after clone induction. Confocal xy-sections (J) and reconstructed xz cross-sections (K, L) at position indicated by dotted line in (J) are shown. Actin is shown in grey (J-L) or red (J'-L'). Cell-autonomous inhibition of apoptosis by *p35* or by *dIAP1* does not prevent cyst formation.

(M) RNA-Seq analysis of *Psc-Su(z)2*^{XL26} mutant wing discs [S1]. *Abd-B* and *lz* are strongly transcriptionally upregulated in *Psc-Su(z)2*^{XL26} mutant cells. *Ubx*, *ci*, *arm* and other signaling components driving cyst formation are not upregulated in *Psc-Su(z)2*^{XL26} mutant discs. RNA-Seq data was validated by real-time PCR experiments [S2] (not shown).

Length of scale bars is indicated in individual images. Related data sets are shown at equal magnifications.

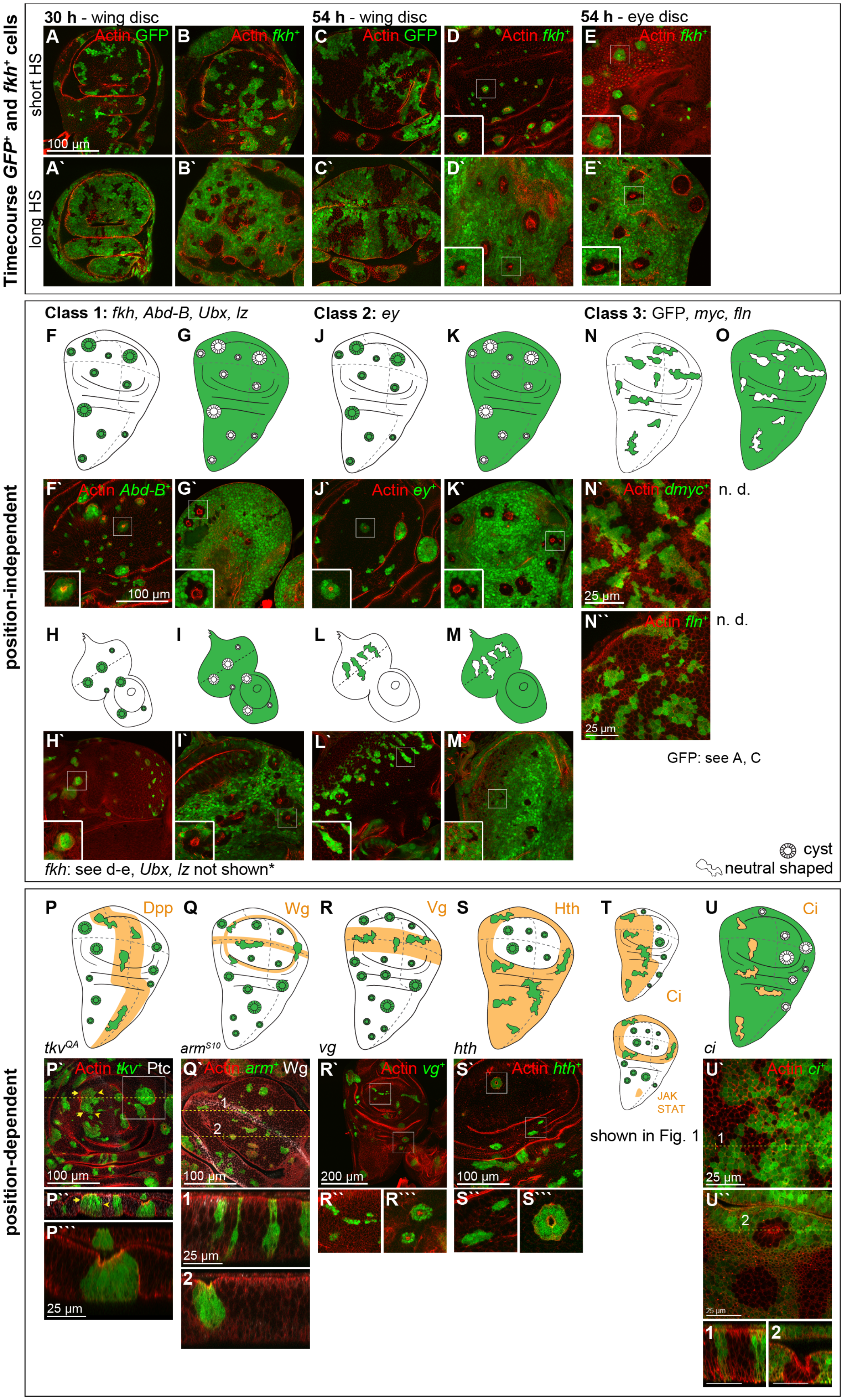
Timecourse GFP+ and fkh+ cells
30 h - wing disc
54 h - wing disc
54 h - eye disc
short HS
long HS
Actin GFP
Actin fkh+
100 µm
position-independent
Class 1: fkh, Abd-B, Ubx, lz
Class 2: ey
Class 3: GFP, myc, fln
Actin Abd-B+
Actin ey+
Actin dmyc+
Actin fln+
n. d.
25 µm
GFP: see A, C
fkh: see d-e, Ubx, lz not shown*
cyst
neutral shaped
position-dependent
Dpp
Wg
Vg
Hth
Ci
JAK STAT
tkvQA
armS10
vg
hth
ci
Actin tkv+ Ptc
Actin arm+ Wg
Actin vg+
Actin hth+
Actin ci+
200 µm
shown in Fig. 1

Figure S2

Ectopic expression of cell fate specifying transcription factors induces cyst formation

Related to Figures 1 and 2

(A-E) Confocal xy-sections of pouch region containing GFP-expressing (A, C) and *fkh*-expressing (B, D) clones as well as eye discs containing *fkh*-expressing clones (E) at 30 h (A, B) and 54 h (C-E) after clone induction using a short (A-E) and long heat-shock (A`-E`). Actin is shown in red, clones in green. White boxes in D, E frame examples of cyst-forming clones shown in small insets.

(F-I) Example of transcription factors that form cysts upon overexpression in wing and eye imaginal disc (*fkh*, *Abd-B*, *Ubx* and *lz*; **lz* only tested in the wing disc). F-I show schematic representation of position-independent cyst formation in the wing (F, G) and eye (H, I) where cysts are formed by misspecified cells (green; F, H) or wild type cells (white; G, I). F`-I` show confocal xy-sections of pouch region (F`, G`) and eye discs (H`, I`) containing *Abd-B*-expressing clones (green) 54 h after clone induction using a short (F`, H`) and long (G`, I`) heat-shock. Actin is shown in red.

(J-M) Overexpression of *ey* leads to cysts in the wing discs but rarely in the eye disc. Schematic representation is shown for wing disc (J, K) and eye discs (L, M). J`-M` show confocal xy-section of pouch region (J`, K`) and eye discs (L`, M`) containing *ey*-expressing clones (green) at 54 h after clone induction using a short (J`, L`) and long (K`, M`) heat-shock. Actin is shown in red.

(N-O) Overexpression of GFP, *myc* and *fln* does not lead to cyst formation. Schematic wing imaginal discs are shown in N, O. Confocal xy-sections of pouch region containing *dMyc*-expressing clones (N`) and *flightin* (*fln*)-expressing clones (N``)(green) at 54 h after clone induction. Actin is shown in red.

(P-U) Examples of position-dependent cyst formation by activation of transcription factors that show a spatially defined expression pattern in the wing imaginal disc. P-U show a schematic representation of wing imaginal discs indicating individual endogenous expression patterns (orange) and regions of cyst formation.

(P`- P```) Confocal xy-section (P`) and xz cross-section (P``, P```) of pouch region containing clones that express a dominant-active version of the *thickveins* (*tkv*) receptor 54 h after clone induction. Actin is shown in red; Ptc staining visualizes the A/P boundary (grey). Dashed line in P` indicates position of cross-section shown in P``. White box frames region of xz cross-section in P```. Note the rough clone boundaries in high Dpp-signaling regions (arrowhead in P`). In contrast, boundaries from the same clone dramatically smoothen (arrow) at basal contacts when they locate outside of Dpp-signaling domains.

**(Q`)** Confocal xy-section (Q`) and xz-cross section (1, 2) of pouch region containing clones (green) that express a dominant-active *armadillo* (*arm*) at 54 h after clone induction. Actin is shown in red, Wg staining in white. Dashed yellow lines indicate position of cross-sections shown in 1 and 2. Note that cysts form in regions of low endogenous Wg signaling (G, H) whereas clones maintain neutral shapes in regions of high Wg signaling.

(R`- R```, S`-S```) Confocal xy-section of pouch region containing clones (green) that express the transcription factor *vestigial* (*vg*, R`-R```) or *homothorax* (*hth*, S`-S```) at 54 h after clone induction. Actin is shown in red. White boxes frame regions, which are shown at higher magnification below. Note the difference in clone smoothening.

**(U`- U``)** Position-dependent cyst formation by wild type cells is illustrated by clonal overexpression of *ci*. Confocal xy-sections (U`, U``) and xz cross-sections (1, 2) of *ci*-expressing clones (green) in the posterior (U`) and anterior (U``) compartment at 54 h after clone induction using a long heat-shock. *ci*-expressing cells are marked by GFP (green), Actin is shown in red. Dashed yellow lines indicate position of cross-sections shown in 1 and 2.

Length of scale bars is indicated in individual images. Related data sets are shown at equal magnifications.

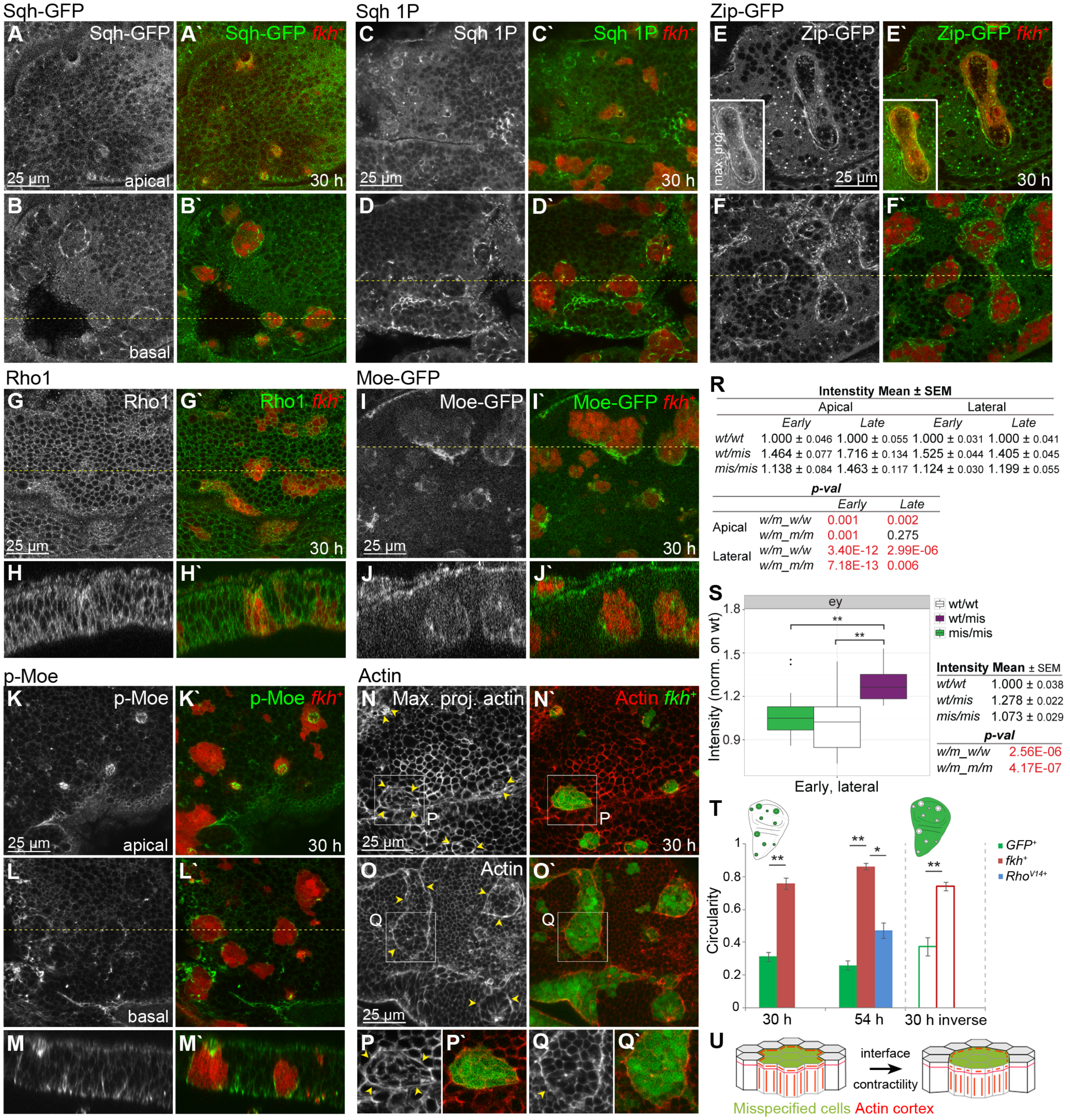

Figure S3

The contractile actomyosin machinery is recruited to the MWI leading to minimization of MWI contact area

Related to Figure 2

(A-F) Single xy-sections through apical (A, C, E) and basolateral (B, D, F) levels of a wing disc pouch containing *fkh*-expressing clones marked by GFP (red in A'-F') at 30 h after clone induction. Additionally, the disc expresses a non-muscle myosin II regulatory light chain GFP-fusion protein (Sqh-GFP, grey in A, B, green in A', B'), stained for the activated phosphorylated form of Sqh (Sqh 1P, grey in C, D, green in C', D') or expressing a non-muscle myosin II heavy chain GFP-fusion protein (Zip-GFP, grey in E, F, green in E',

F'). The dotted lines in the basolateral xy-sections (B, D, F) indicate positions of xz cross-sections shown in Figure 2G-I. Insets in (E) are maximum projections of all apical sections. Note the additional strong enrichment of actomyosin components at basolateral MWI's (B, D, F).

(G-J) Single xy-sections (G, I) and reconstructed xz cross-sections (H, J) of a wing imaginal disc containing *fkh*-expressing clones marked by GFP (red in G'-J') at 30 h after clone induction. Additionally, the disc is stained for the GTPase Rho1 (grey in G, H and green in G', H') or expresses a Moesin-GFP-fusion protein (Moe-GFP) (grey in I, J and green in I', J'). The dotted lines in (G, I) indicate positions of xz cross-sections (H, J). Whereas Moe-GFP enriches at lateral MWI's, Rho1 does not specifically enrich there.

(K-Q) Single xy-sections through apical (K, N, P) and basolateral (L, O, Q) levels of a wing disc pouch containing *fkh*-expressing clones marked by GFP (red in K'-M', green in N'-Q') at 30 h after clone induction. Additionally, the disc was stained for the phosphorylated form of Moesin (p-Moe, green in K'-M', grey in K-M) or stained with phalloidin (red in N'-Q' and grey in N-Q) to visualize accumulation of actin at apical adherens junctions (N, P) and basal integrin junctions (O, Q) at the MWI (arrowheads).

The dotted line in (L) indicates positions of xz cross-sections (M). Boxes in (N, O) frame regions shown at higher magnification in (P, Q).

(R) Normalized mean intensities and SEM of actin intensity measurements at apical and lateral domains in discs containing *fkh*-expressing cells (top table corresponds to data visualized as box plots in Fig. 2E, F). Data of experimental conditions where *fkh*-expressing clones (10 min heat-shock) and wild type cells (25 min heat-shock) would form cysts were pooled to reflect the mirror symmetry of the cyst-forming conditions. Actin intensities at interfaces between wild type cell (wt/wt), between misspecified *fkh*-expressing cells (mis/mis) and at the interface between wild type and *fkh*-expressing cell (wt/mis) are shown for early stages (30 h) and late stages (54 h) after induction of *fkh*-expressing cells.

Significance of differences between data sets (table below) was tested by applying a two-tailed WSR-test. Calculated p-values are shown. Red = p-val < 0.01. Number of clones analyzed for basolateral actin intensity: 10min heat-shock, 30 h, n=49 clones; 10min heat-shock, 54 h, n=17 clones; 25 min heat-shock, 30h, n=38 clones; 25 min heat-shock, 54 h, n=14 clones. Number of clones analyzed for apical actin intensity: 10min heat-shock, 30h, n=15 clones; 10min heat-shock, 54 h, n=7 clones; 25 min heat-shock, 30 h, n=10 clones; 25 min heat-shock, 54 h, n=3 clones. Lack of statistically significant differences for apical actin intensities at late stages are due to mild cell-autonomous enrichment of actin in *fkh*-expressing cells.

(S) Actin intensity at basolateral interfaces between misspecified *ey*-expressing cells (mis/mis), wild type cells (wt/wt), and between wild type and misspecified *ey*-expressing cells (wt/mis) 30 h after clone induction. Data of experimental conditions, where *ey*-expressing clones (10min heat-shock, n=22 clones) and wild type clones (25 min heat-shock, n=10 clones) were pooled to reflect the mirror symmetry of cyst-forming conditions. A box plot and a table displaying mean and SEM of the data are shown. Significance of differences between data sets was tested by applying a two-tailed WSR test. ** = p-val < 0.001. Calculated p-values are shown in the table. Red = p-val < 0.01.

(T) Clone circularity represented in a bar graph and schematic representation of conditions for which clone circularity was determined. Solid colored bars represent circularity for neutral wild type clones expressing GFP (green), and *fkh*-expressing clones (red) or Rho^{V14}, *p35*-expressing clones (blue) giving rise to cysts, early (30 h) and late (54 h) after clone induction. White bars represent circularity for wild type clones if surrounded by neutral GFP-expressing cells (green outline) or by *fkh*-expressing cells (red outline) at early stages after induction (30 h). Number of clones analyzed: GFP 30 h (n=10), *fkh* 30 h (n=10), GFP 54 h (n=6), *fkh* 54 h (n=11), *Rho*^{V14} 54 h (n=11), GFP inv 30 h (n=9), *fkh* inv 30 h (n=9). Inv (inverse) describes

conditions where wild type cells were surrounded by either GFP or *fkh*-expressing cells. Graph represents the mean and SEM of the data. Significance of differences between data sets was tested by applying a two-tailed WMW test. * = p-val < 0.01, ** = p-val < 0.001

(U) Schematic representation of the effect of actin recruitment to the MWI (red) leading to increased apical and basolateral MWI contractility, as well as MWI minimization and decreased roughness.

Length of scale bars is indicated in individual images. Related data sets are shown at equal magnifications.

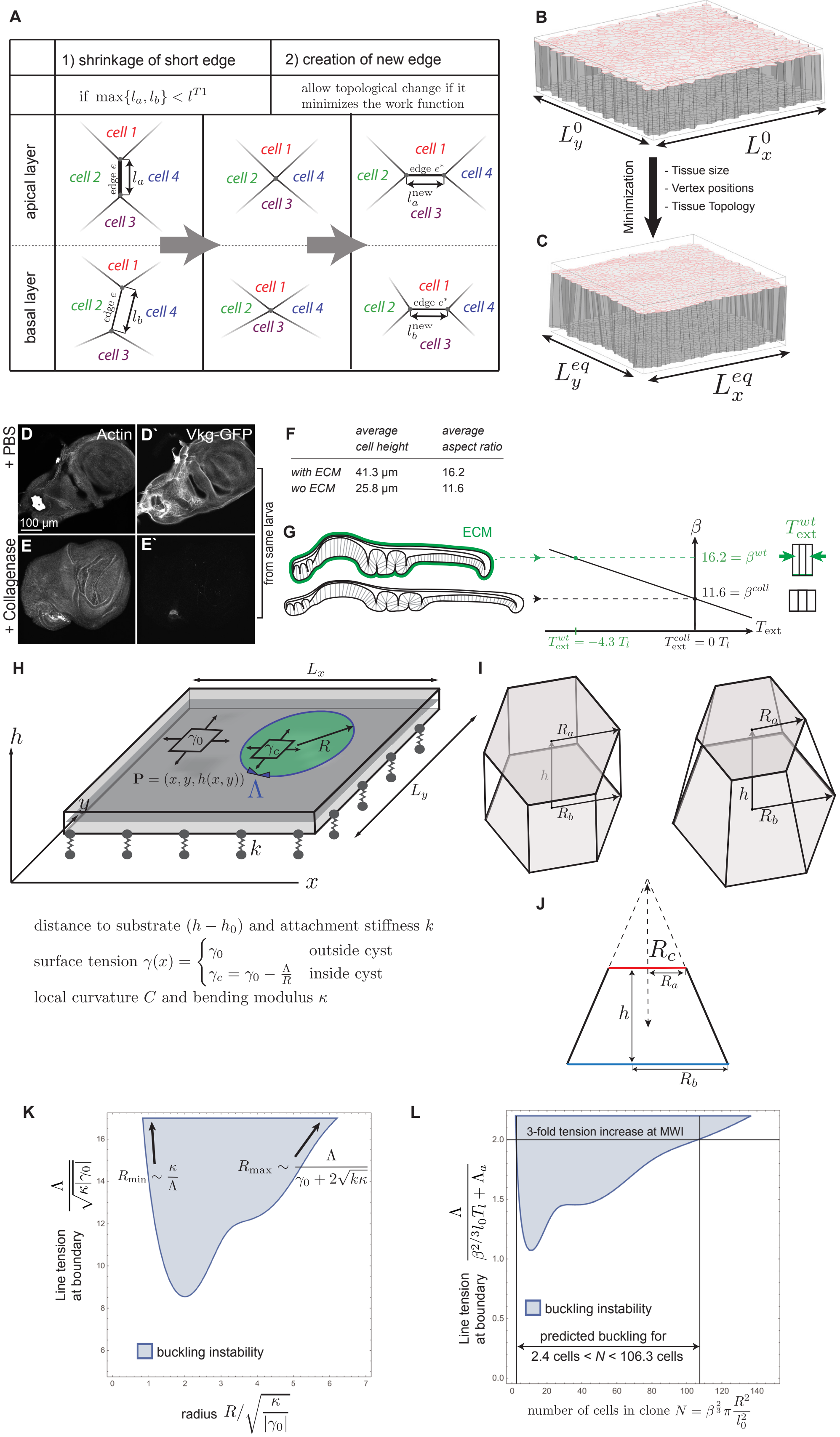
A
1) shrinkage of short edge
2) creation of new edge
if max{l_a, l_b} < l^{T1}
allow topological change if it minimizes the work function
apical layer
basal layer
cell 1
cell 2
cell 3
cell 4
edge e
edge e*
B
C
Minimization
- Tissue size
- Vertex positions
- Tissue Topology
D
Actin
D`
Vkg-GFP
E
E`
+ PBS
+ Collagenase
100 µm
from same larva
F
average cell height
average aspect ratio
with ECM 41.3 µm 16.2
wo ECM 25.8 µm 11.6
G
ECM
16.2 = β^{wt}
11.6 = β^{coll}
$T^{wt}_{ext} = -4.3\ T_l$
$T^{coll}_{ext} = 0\ T_l$
H
$\mathbf{P} = (x, y, h(x, y))$
I
J
distance to substrate $(h - h_0)$ and attachment stiffness k
surface tension $\gamma(x) = \begin{cases} \gamma_0 & \text{outside cyst} \\ \gamma_c = \gamma_0 - \frac{\Lambda}{R} & \text{inside cyst} \end{cases}$
local curvature C and bending modulus κ
K
Line tension at boundary $\frac{\Lambda}{\sqrt{\kappa|\gamma_0|}}$
$R_{\min} \sim \frac{\kappa}{\Lambda}$
$R_{\max} \sim \frac{\Lambda}{\gamma_0 + 2\sqrt{k\kappa}}$
buckling instability
radius $R/\sqrt{\frac{\kappa}{|\gamma_0|}}$
L
3-fold tension increase at MWI
Line tension at boundary $\frac{\Lambda}{\beta^{2/3} l_0 T_l + \Lambda_a}$
buckling instability
predicted buckling for
2.4 cells < N < 106.3 cells
number of cells in clone $N = \beta^{\frac{2}{3}} \pi \frac{R^2}{l_0^2}$

Figure S4

3D Vertex Model implementation and continuum model description of cyst formation

Related to Figure 3 and 4

(A) A T_1 transition in the 3D Vertex Model represents an exchange of cell neighbors, where topological transitions take place both on apical and basal networks.

1) If an edge's (e) apical and basal lengths (l_a, l_b) fall beyond a predefined threshold (l_{T1}), the edge is replaced by a vertex.

2) A new edge replacing the vertex is then chosen as described in the Suppl. Information (e^*). The initial apical and basal lengths of e^* of the new edge are set to l_a^{new} and l_b^{new} respectively.

(B, C) To generate randomized wild type tissues, a 2D Voronoi tesselation for randomly distributed points inside a periodic box with sizes L_x^0 and L_y^0 was created (B). This initial tissue is relaxed with respect to the size of the periodic box (L_x^{eq}, L_y^{eq}), to the positions of the vertices inside the box and with respect to topological changes (C).

(D, E) Wing imaginal discs siblings expressing Vkg-GFP (grey in D`, E`) and stained for Actin (grey in D, E). One sibling disc was treated for 12 min with collagenase (E), the other with PBS only (D). Note absence of Vkg-GFP signal after collagenase treatment (E`) and increase in disc area (compare E with D).

(F) The aspect ratio β of a cell is defined to be the ratio between its height and the square root of its apical surface area. After collagenase treatment pouch cells take on a more cuboidal shape and their average aspect ratio decreases from 16.2 to 11.6.

(G) Tissue aspect ratio as a function of the external tension applied on the tissue, if all mechanical parameters other than T_{ext} are chosen as in Table 1. If the wing disc is under external compression due to the ECM, the compression is removed by collagenase treatment. The aspect ratio of uncompressed cells relates to the relative strength of the lateral surface tension. The aspect ratio of compressed cells can then be used to estimate the extent of external compression.

(H) In a continuum description, the tissue is described as a thin membrane, assuming that the height of the tissue is small compared to the length scale of deformations. The position of points in the tissue mid-plane are given in the Monge gauge by their heights $h(x, y)$. The membrane representing the tissue has a bending rigidity κ penalizing deviations of the local mean curvature C from the spontaneous curvature C_0, is subjected to an external surface tension γ_0, and is attached by elastic linkers with elastic modulus k to the underlying ECM, represented as a flat surface at $z = 0$. The misspecified clone with radius R is surrounded by the MWI, which exerts an effective line tension Λ, inducing a size-dependent compression γ_c acting on the clone. The resulting work function is given in Eq. 24.

(I) The continuous model parameters are related to the 3D vertex model parameters by considering a packing of identical hexagonal cells with apical and basal radii R_a and R_b and height h.

(J) For a 3D packing of identical cells, the local radius of curvature of the tissue mid-plane R_c can be related to the cell's apical and basal radii R_a and R_b.

(K) Stability diagram of a circular tissue region clamped at its boundary, under negative surface tension γ_0 and subjected to an additional line tension acting along its boundary. The blue region depicts the regime of unstable tissue sizes for normalized strength of the boundary effect $\Lambda/\sqrt{\kappa|\gamma_0|}$, and fixed attachment stiffness $k/(\gamma_0^2/\kappa) = 3$.

(L) Stability diagram of a circular tissue region clamped at its boundary and subjected to a line tension along its boundary, with parameters of the continuum theory related to vertex model parameters (section 4.5 of Suppl. Information). Parameters ($\kappa/(T_l l_0^2) \simeq 0.34$, $\gamma_0/T_l \simeq -4.2$, $k/(T_l/l_0^2) \simeq 64$) are obtained by taking

vertex model parameters of Table 1 in the Suppl. Information and estimating the corresponding continuum theory parameters. The black line indicates a 3-fold increase in apical line and lateral surface tension at the MWI.

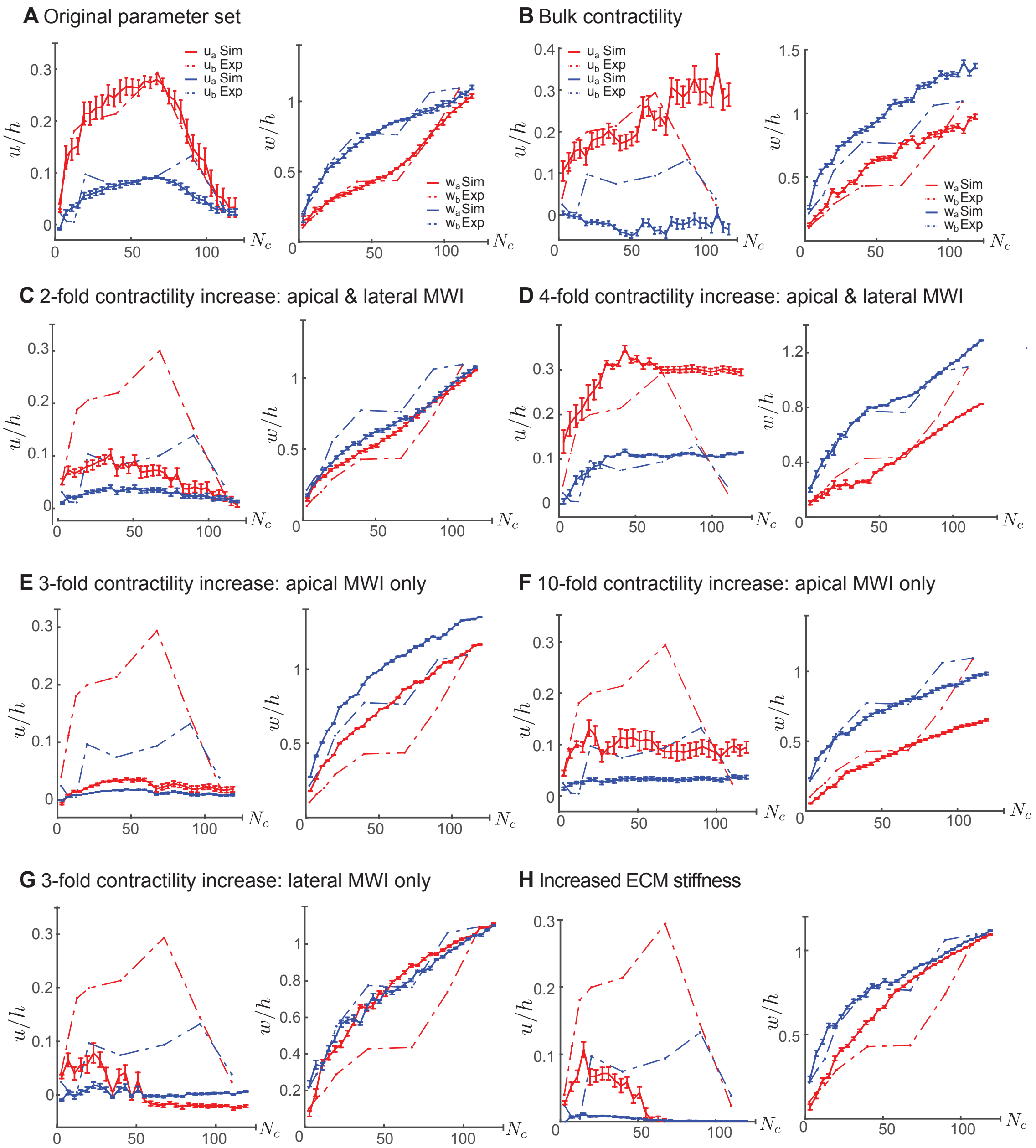

Figure S5

Parameter variation for cyst simulations in the 3D vertex model

Related to Figure 5

Variations of mechanical parameters around parameters fitted to experimental observations. Mechanical parameters for each set of simulations are the same as in Figure 4, except for indicated parameter changes and values marked by an asterisk in equations below. Graphs show mean and SEM of 15 simulations. Graphs illustrate experimental (dotted line) and simulated (continuous line) deformation of apical (red) or basal (blue) cyst surfaces with respect to clone size. Parameters u_a, u_b, w_a and w_b are illustrated in a schematic in Fig. 5E, and are normalised with the height h of the surrounding tissue.

(A) Original parameter set as given in Table 1 in the Supplementary Modeling Procedures with a 3-fold

increase in apical line and lateral surface tension at the MWI.

(B) Simulations with 3-fold increased lateral surface tension in misspecified cells only. Large clones are strongly deformed by such a perturbation, in disagreement with experimental observations.
($T_l^{(mis/mis,*)} = 3T_l^{(wt/wt)}$, $T_l^{(wt/mis,*)} = 2T_l^{(wt/wt)}$)

(C) Simulations with increased apical line and lateral surface tensions at the MWI by a factor of 2. The clone deformation is weaker than observed in experiments.
($T_l^{(wt/mis,*)} = 2T_l^{(wt/wt)}$, $\Lambda_a^{(wt/mis,*)} = 2\Lambda_a^{(wt/wt)}$)

(D) Simulations with increased apical line and lateral surface tensions at the MWI by a factor of 4. The clone deformation is larger than observed in experiments.
($T_l^{(wt/mis,*)} = 4T_l^{(wt/wt)}$, $\Lambda_a^{(wt/mis,*)} = 4\Lambda_a^{(wt/wt)}$)

(E-F) Simulations with 3 and 10 fold increased apical line tension alone at the MWI, and no increase in lateral surface tension. Experimentally observed apical indentation is not recapitulated in these conditions. This suggests that the contribution of contractility at basolateral interfaces is necessary to describe the observed clone shapes.
(E: $T_l^{(wt/mis,*)} = T_l^{(wt/wt)}$, $\Lambda_a^{(wt/mis,*)} = 3\Lambda_l^{(wt/wt)}$;
$F : T_l^{(wt/mis,*)} = T_l^{(wt/wt)}$, $\Lambda_a^{(wt/mis,*)} = 10\Lambda_a^{(wt/wt)}$)

(G) Simulations with 3-fold increase in lateral surface tension at the MWI, and no increase in apical line tension. Experimentally observed apical indentation and apicobasal asymmetry is not recapitulated in these conditions.This indicates that the additional symmetry breaking by the increase in apical line tension at the MWI is required to explain the observed deformations.
($T_l^{(wt/mis,*)} = 3T_l^{(wt/wt)}$, $\Lambda_a^{(wt/mis,*)} = \Lambda_a^{(wt/wt)}$)

(H) Simulations with increased stiffness of attachment to the ECM. With increasing attachment stiffness the apical and basal surfaces deform less.
($k^* = 2k$)

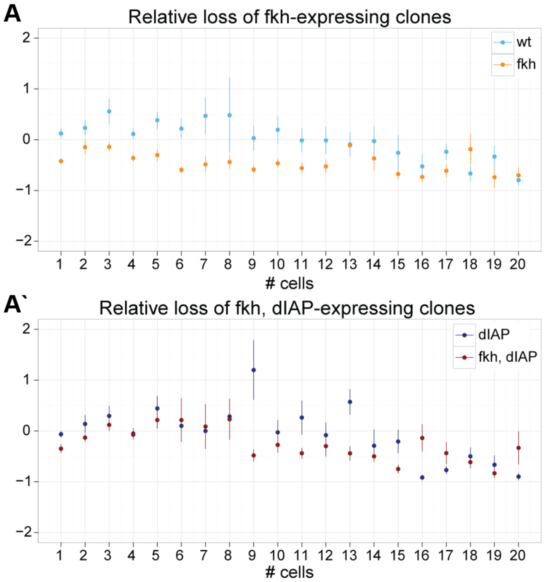

B

# cells in clone	*p-val* wt to fkh	*p-val* dIAP to fkh, dIAP
1	0.0003	0.0075
2	0.0481	0.1305
3	0.0014	0.3519
4	0.0023	0.4396
5	0.0026	0.3804
6	0.0004	0.2591
7	0.0205	0.3240
8	0.0698	0.1805
9	0.0055	0.0213
10	0.0315	0.1700
11	0.0221	0.0385
12	0.1042	0.2336
13	0.5000	0.0071
14	0.2138	0.5000
15	0.4595	0.0404
16	0.5330	0.9969
17	0.0777	0.8200
18	0.8208	0.2214
19	0.0649	0.1970
20	0.5790	0.7686

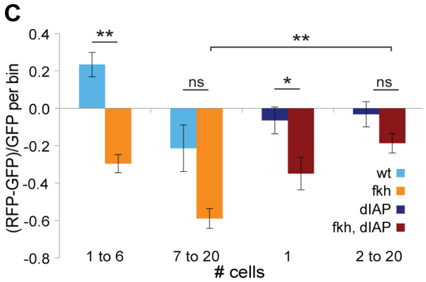

D

# cells	*p-val* fkh	fkh, dIAP	fkh_fkh, dIAP
1	2.89E-04	0.0075	--
1 to 6	1.42E-04	--	--
2 to 6	5.41E-06	0.3283	0.0059
2 to 20	--	0.0864	--
7 to 20	1.16E-02	0.0092	0.0273

E

	p-val fkh_fkh, dIAP
1 cell	0.0277
2 to 6	0.0021
>6	0.0026

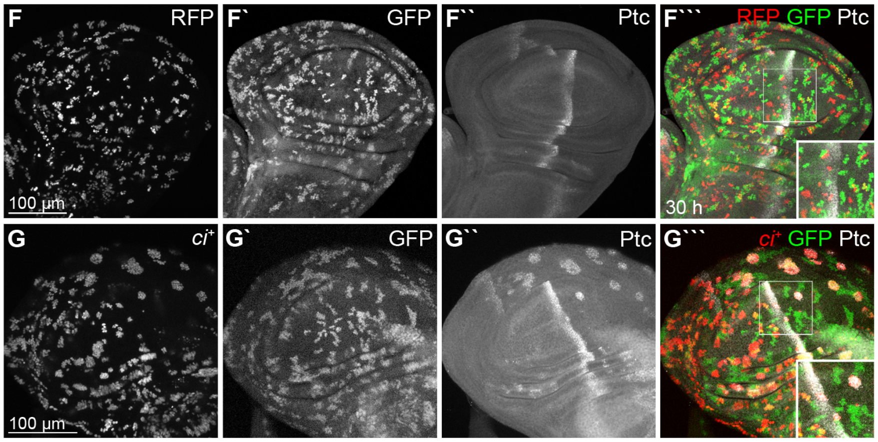

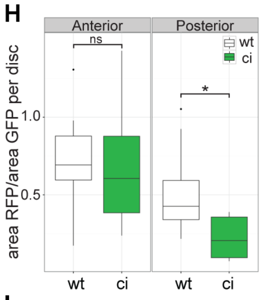

I

Mean ± SEM	Anterior	Posterior
wt	0.73 ± 0.09	0.51 ± 0.08
ci	0.68 ± 0.14	0.22 ± 0.04

p-val	Anterior	Posterior
wt_ci	0.228	0.001572

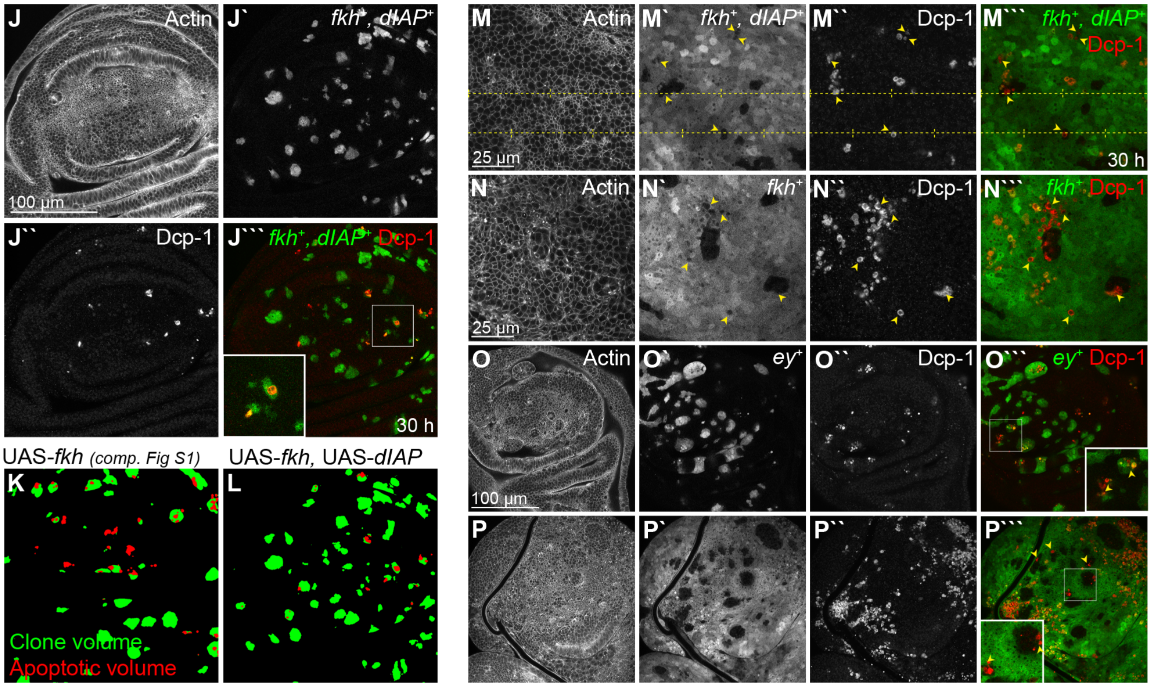

Figure S6

Misspecified cells are eliminated from imaginal discs by MWI contractility

Related to Figure 6

(A) Relative loss of (A) *fkh* and (A') *fkh*, *dIAP1*-expressing RFP-positive clones compared to control RFP-positive clones ((A) wt and (A') *dIAP1*-expressing clones, respectively) in Tie-Dye wing discs. RFP clones expressed either RFP alone (light blue), *fkh* (light red), *dIAP1* (dark blue) or *fkh*, *dIAP1* (dark red). For each disc, the number of control GFP-expressing clones was subtracted from the number of RFP-clones and normalized to the number of GFP-clones for the respective clone size bin. This analysis utilizes the strength of the Tie-Dye system as the data is normalized to intrinsic GFP-positive control clones for each clone size bin and genotype.

Graphs represent the mean and SEM of the data (n=10 discs for wt, *fkh*, *fkh*, *dIAP1* each; n=8 discs for *dIAP1*). Number of GFP clones analyzed: GFP clones: wt (n=1738), *fkh* (n=1714), *dIAP1* (n=1431), *fkh*, *dIAP1* (n=1572). RFP clones: wt (n=1861), *fkh* (n=997), *dIAP1* (n=1381), *fkh*, *dIAP1* (n=1221)

(B) Table listing p-values calculated to test for statistical significant difference in relative loss of RFP clones (A, A') per clone size bin utilizing a one-tailed WMW-test. Red = p-val < 0.01. The analysis suggests that *fkh*-expressing clones of less than 6 cells and *fkh*, *dIAP1*-expressing clones of 1 cell are significantly under-represented in the data set. We thus chose these thresholds for further analysis.

(C) Analysis of relative loss of *fkh* and *fkh*, *dIAP1*-expressing RFP-positive clones compared to relevant control RFP-positive clones binned into two statistically significant clone size categories (*fkh* 1-6 cells and above 6 cells, *fkh*, *dIAP1* 1 cell clones and above 1 cell clones). Color code, genotype pairing and calculations are the same as in Fig. 6G and S6A. All graphs report mean and SEM. Significant differences between non-parametrically distributed data sets was tested by applying a one-tailed WMW test. A one-tailed Welch's t test was applied to normally distributed data sets with unequal variances. * = p-val < 0.01, ** = p-val < 0.001, ns= not significant. See Fig. S6D for detailed p-values. 10 discs were analyzed for wt, *fkh* and *fkh*, *dIAP1* genotypes each, 8 discs for *dIAP1* controls.

(D) Table listing p-values calculated to test for statistical significant difference in relative loss of RFP clones (data shown in Fig. 6G and Fig. S6C) per clone size bin as indicated. Significant differences between non-parametrically distributed data sets were tested by applying a one-tailed WMW test. A one-tailed Welch's t test was applied to normally distributed data sets with unequal variances. Red = p-val < 0.01.

(E) Table listing p-values calculated to test for statistical significant difference in relative apoptotic volume ratios between *fkh* and *fkh*, *dIAP1*-expressing clones (data shown in Fig. 6I) per clone size bin as indicated. Significant differences between data sets were tested by applying two-tailed WMW test. Red = p-val < 0.01.

(F, G) Tie-Dye imaginal discs 30 h after clone induction carrying neutral RFP-expressing clones (grey in F, red in F''') or *ci*, RFP-expressing clones (grey in G, red in G''') and neutral GFP-expressing clones (grey in F', G', green in F''', G'''). Discs were stained for Patched (Ptc) (grey in F'', G'', F''', G''') to visualize the A/P boundary. White boxes frame higher magnification insets for overlays shown in F''' and G'''. Note general loss of *ci*-expressing clones and persistence of larger clones in the posterior compartment.

(H, I) Boxplot (H) and mean relative area (I) occupied by neutral RFP-expressing or *ci*, RFP-expressing clones normalized to neutral GFP clone area in the anterior and posterior compartment. Absolute clone areas were quantified in 9 wt control and 11 *ci*-expressing Tie-Dye discs at 30 h after clone induction. Significance of differences between data sets was tested by applying a two-tailed WMW test. (H) * = p-val < 0.01, ns= not significant. (I) Red = p-val < 0.01. **(J)** Single basolateral xy-sections of a wing pouch containing *fkh*, *dIAP1*-expressing clones at 30 h after clone induction. Clones are marked by expression of GFP (J',

green in J'''). The disc was stained for phalloidin (J) and the apoptotic marker Dcp-1 (J'', red in J'''). White box frames higher magnification inset shown in J'''.

(K, L) Examples of Weka segmented masks (Fiji) used for quantitative analysis in Fig. 6 H, I derived from images in Fig. S1I and Fig. S6J. Apoptotic volume (red) was tracked for each clone volume (green) in 3D. A single xy-section is shown.

(M) Single basal xy-section of a wing pouch containing *fkh*, *dIAP1*-expressing cells (M', green in M''') at 30 h after clone induction. The disc was stained for phalloidin (M) and the apoptotic marker Dcp-1 (M'', red in M'''). Much of the apoptotic signal derives from wild type cells (examples highlighted by yellow arrowheads). Dotted lines indicate positions at which xz cross-sections shown in Fig. 6J, K were reconstructed.

(N) Single basal xy-sections of a wing pouch containing *fkh*-expressing cells (N', green in N''') at 30 h after clone induction. The disc was stained for phalloidin (N) and the apoptotic marker Dcp-1 (N'', red in N'''). Much of the apoptotic signal derives from wild type cells (examples highlighted by yellow arrowheads).

(O, P) Single xy-sections of a wing discs containing *ey*-expressing cells (O', P', and green in O''', P''') induced by a short 8 min heat-shock (O) and a long 25 min heat-shock (P), imaged 30 h after clone induction. The disc was stained for phalloidin (O, P) and the apoptotic marker Dcp-1 (O'', P'', red in O''', P'''). Whereas apoptosis is mainly observed in small *ey*-expressing clones (O), much of the apoptotic signal derives from wild type cells if surrounded by large domains of *ey*-expressing cells (P).

Table S1

Genotypes and experimental conditions

Detailed genotypes and experimental conditions (time-point of dissection, heat-shock length, egg collection/larval staging and use of spacer between coverslip and slide for microscopy) of data represented in individual figures

Figure	Genotype	Time points	Heat-shock	Egg-coll.	spacer
	Figure 1				
1 A	*hsflp [122] /+; FRT42D ubi-eGFP / FRT42D Psc-Su(z)2[XL26]; +/+*	54 h	40 min	no	no
1 C	*hsflp [122] /+; FRT42D ubi-mRFP / FRT42D Psc-Su(z)2[XL26]; +/+*	0 h	45 min	no	yes
1 D	*hsflp [122] /+; FRT42D ubi-mRFP / FRT42D Psc-Su(z)2[XL26]; +/+*	30 h	45 min	no	yes
1 E, F	*hsflp [122] /+; FRT42D ubi-mRFP / FRT42D Psc-Su(z)2[XL26]; +/+*	54 h	45 min	no	yes
1 G	*UAS-fkh-3xHA / hsflp [122]; + / +; act > y[+] > GAL4, UAS-GFP/+*	0h	10 min	no	yes
1 H	*UAS-fkh-3xHA / hsflp [122]; + / +; act > y[+] > GAL4, UAS-GFP/+*	30 h	10 min	yes	yes
1 I	*UAS-fkh-3xHA / hsflp [122]; + / +; act > y[+] > GAL4, UAS-GFP/+*	54 h	10 min	yes	yes
1 J-M	*hsflp [122] / +; + / +; act > y[+] > GAL4, UAS GFP / UAS-ci.HA.wt*	54h	10 min	no	yes
1 N-Q	*hsflp [122] / +; + / UAS-hop[TumL]; act > y[+] > GAL4, UAS GFP / +*	54 h	8 min	no	yes
	Figure 2				
2 A	*UAS-fkh-3xHA / hsflp [122]; + / +; act > y[+] > GAL4, UAS-GFP/+*	30 h	25 min	yes	yes
2 B	*hsflp [122] / +; + / UAS-ey ; act > y[+] > GAL4, UAS GFP/ +*	30 h	25 min	yes	yes
2 D	*UAS-fkh-3xHA / hsflp [122]; + / +; act > y[+] > GAL4, UAS-GFP/+*	30 h	10 min	yes	yes
2 G	*hsflp [122] / UAS-fkh-3xHA; + / sqh-GFP; Act5C.GAL4 (FRT.CD2), UAS-RFP / +*	30 h	10 min	no	yes
2 H	*UAS-fkh-3xHA / hsflp [122]; + / +; act > y[+] > GAL4, UAS-GFP/+*	30 h	10 min	no	yes
2 I	*hsflp [122] / UAS-fkh-3xHA; + / zip>GFP; Act5C.GAL4 (FRT.CD2), UAS-RFP /+*	30 h	10 min	no	yes
2 J-K	*hsflp [122] / +; FRT42D ubi-mRFP / FRT42D Psc-Su(z)2[XL26], vkg>GFP; +/+*	54 h	40 min	no	yes
2 M, N	*hsflp [122]/ +; + / +; act > y[+] > GAL4, UAS-GFP/ +*	54 h	10 min	yes	yes
2 L, O	*UAS-fkh-3xHA / hsflp [122]; + / +; act > y[+] > GAL4, UAS-GFP/+*	54 h	10 min	yes	yes
	Figure 4				
3 F, G	*hsflp [122] / +; + / UAS-p35; Act5C.GAL4 (FRT.CD2), UAS-RFP / UAS-RhoV14*	54 h	10 min	no	no
3 H	*hsflp [122] / +; + / UAS-p35; Act5C.GAL4 (FRT.CD2), UAS-RFP / UAS-RhoV14*	54 h	10 min	no	yes
3 I-J	*hsflp [122] / + ; + / UAS-p35; act > y[+] > GAL4, UAS-GFP / UAS-RhoV14*	54 h	20 min	no	yes

	Figure 5				
4 F-J	*UAS-fkh-3xHA / hsflp $^{[122]}$; + / +; act > y$^{[+]}$ > GAL4, UAS-GFP/+*	30 h	10 min	yes	yes
	Figure 6				
5 A	*hsflp$^{[122]}$/+; ubi <stop <GFP^{nls}, act5C <stop <lacZ^{nls}/+; Act5C <stop <GAL4, UAS-his2A::RFP/+*	30h	7 min	24h	no
5 B	*UAS-fkh-3xHA/ hsflp$^{[122]}$; ubi <stop <GFP^{nls}, act5C <stop <lacZ^{nls}/+; Act5C <stop <GAL4, UAS-his2A::RFP/+*	30h	7 min	24h	no
5 C	*hsflp$^{[122]}$/ Y; ubi <stop <GFP^{nls}, act5C <stop <lacZ^{nls}/Sp or +; Act5C <stop <GAL4, UAS-his2A::RFP/UAS-dIAP*	30h	7 min	24h	no
5 D	*UAS-fkh-3xHA/ hsflp$^{[122]}$; ubi <stop <GFP^{nls}, act5C <stop <lacZ^{nls}/Sp or +; Act5C <stop <GAL4, UAS-his2A::RFP/UAS-dIAP*	30h	7 min	24h	no
5 J-K	*UAS-fkh-3xHA / hsflp $^{[122]}$; + / +; act > y$^{[+]}$ > GAL4, UAS-GFP/UAS-dIAP*	30 h	30 min	no	no
5 L,M,O	*hsflp $^{[122]}$/ Y or +; UAS-Ras^{V12} / + ; act > y$^{[+]}$ > GAL4, UAS-GFP/+*	30h	10 min	no	no
5 P,Q,R	*hsflp $^{[122]}$/ Y or +; UAS-Ras^{V12} / + ; act > y$^{[+]}$ > GAL4, UAS-GFP/+*	30h	27 min	no	no
5 N	*hsflp $^{[122]}$/ Y or +; Sp / CyO ; act > y$^{[+]}$ > GAL4, UAS-GFP/+*	30 h	10 min	no	no
	Figure S1				
S1 A-B	*hsflp $^{[122]}$/+; FRT42D ubi-eGFP / FRT42D Psc-Su(z)2$^{[1b8]recA}$; +/+*	27 h	60 min	no	no
S1 C	*hsflp $^{[122]}$/+; FRT42D ubi-eGFP / FRT42D Psc-Su(z)2$^{[1b8]recA}$; +/+*	72 h	60 min	no	no
S1 D-E	*hsflp $^{[122]}$/+; FRT42D ubi-eGFP / FRT42D Psc-Su(z)2$^{[1b8]recA}$, yki$^{[B5]}$; +/+*	27 h	60 min	no	no
S1 F	*hsflp $^{[122]}$/+; FRT42D ubi-eGFP / FRT42D Psc-Su(z)2$^{[1b8]recA}$, yki$^{[B5]}$; +/+*	72 h	60 min	no	no
S1 G	*hsflp $^{[122]}$/+; FRT42D ubi-eGFP / FRT42D Psc-Su(z)2$^{[XL26]}$; +/+*	54 h	40 min	no	no
S1 H	*hep$^{[r75]}$/Y; FRT42D ubi-eGFP / FRT42D Psc-Su(z)2$^{[XL26]}$; T155 Gal4 UAS-flp/+*	/	/	no	no
S1 I	*UAS-fkh-3xHA / hsflp $^{[122]}$; + / +; act > y$^{[+]}$ > GAL4, UAS-GFP/+*	30 h	7 min	no	no
S1 J-K	*UAS-fkh-3xHA / hsflp $^{[122]}$; + / UAS-p35; act > y$^{[+]}$ > GAL4, UAS-GFP/ +*	54 h	10 min	no	yes
S1 L	*UAS-fkh-3xHA / hsflp $^{[122]}$; + / +; act > y$^{[+]}$ > GAL4, UAS-GFP/ UAS-dIAP*	54 h	10 min	no	yes
	Figure S2				
S2 A	*hsflp $^{[122]}$/ +; + / +; act > y$^{[+]}$ > GAL4, UAS-GFP/ +*	30 h	10 min	yes	yes
S2 A\`	*hsflp $^{[122]}$/ +; + / +; act > y$^{[+]}$ > GAL4, UAS-GFP/ +*	30 h	25 min	yes	yes
S2 B	*UAS-fkh-3xHA / hsflp $^{[122]}$; + / +; act > y$^{[+]}$ > GAL4, UAS-GFP/+*	30 h	10 min	yes	yes
S2 B\`	*UAS-fkh-3xHA / hsflp $^{[122]}$; + / +; act > y$^{[+]}$ > GAL4, UAS-GFP/+*	30 h	25 min	yes	yes
S2 C	*hsflp $^{[122]}$/ +; + / +; act > y$^{[+]}$ > GAL4, UAS-GFP/ +*	54 h	10 min	yes	yes

S2 C`	*hsflp* $^{[122]}$*/ +; + / +; act > y*$^{[+]}$ *> GAL4, UAS-GFP/ +*	54 h	25 min	yes	yes
S2 D, E	*UAS-fkh-3xHA / +, act > CD2 > GAL4, UAS GFP/ +; MKRS hsflp / +*	54 h	60 min	no	no
S2 D`, E`	*UAS-fkh-3xHA / hsflp* $^{[122]}$*; + / +; act > y*$^{[+]}$ *> GAL4, UAS-GFP/+*	54 h	40 min	no	no
S2 H`	*act > CD2 > GAL4, UAS GFP/ UAS-AbdB; MKRS hsflp / +*	54 h	60 min	no	no
S2 F`	*hsflp* $^{[122]}$ */ +; + / UAS-AbdB ; act > y*$^{[+]}$ *> GAL4, UAS GFP/ +*	54 h	10 min	no	no
S2 G`, I`	*hsflp* $^{[122]}$ */ +; + / UAS-AbdB ; act > y*$^{[+]}$ *> GAL4, UAS GFP/ +*	54 h	40 min	no	no
S2 L`	*act > CD2 > GAL4, UAS GFP/ UAS-ey; MKRS hsflp / +*	54 h	60 min	no	no
S2 J`	*hsflp* $^{[122]}$ */ +; + / UAS-ey ; act > y*$^{[+]}$ *> GAL4, UAS GFP/ +*	54 h	10 min	no	no
S2 K`, M`	*hsflp* $^{[122]}$ */ +; + / UAS-ey ; act > y*$^{[+]}$ *> GAL4, UAS GFP/ +*	54 h	40 min	no	no
S2 N``	*hsflp* $^{[122]}$ */ +; + / + ; act > y[+] > GAL4, UAS GFP/ UAS-fln-HA3*	54 h	10 min	no	no
S2 N`	*hsflp* $^{[122]}$ */ +; +; act > y[+] > GAL4, UAS GFP/ UAS-dmyc*	54 h	10 min	no	yes
S2 P`-P```	*hsflp* $^{[122]}$ */ +; +/ +; act > y*$^{[+]}$ *> GAL4, UAS GFP / UAS-tkv.CA*	54 h	10 min	no	no
S2 Q`-Q```	*hsflp* $^{[122]}$ */ UAS-armS10; + / +; act > y*$^{[+]}$ *> GAL4, UAS GFP / +*	54 h	10 min	no	no
S2 R`-R```	*hsflp* $^{[122]}$ */ +; + / +; Act5C.GAL4 (FRT.CD2), UAS-RFP / UAS-vg*	54 h	10 min	no	no
S2 S`-S```	*hsflp* $^{[122]}$ */ +; + / +; act > y*$^{[+]}$ *> GAL4, UAS GFP / UAS-hth*	54 h	10 min	no	no
S2 U`-U``	*hsflp* $^{[122]}$ */ +; + / +; act > y*$^{[+]}$ *> GAL4, UAS GFP / UAS-ci.HA.wt*	54 h	30 min	no	yes
	Figure S3				
S4 A-B	*hsflp* $^{[122]}$ */ UAS-fkh-3xHA; + / sqh-GFP; Act5C.GAL4 (FRT.CD2), UAS-RFP / +*	30 h	10 min	no	yes
S4 C-D	*UAS-fkh-3xHA / hsflp* $^{[122]}$*; + / +; act > y*$^{[+]}$ *> GAL4, UAS-GFP/+*	30 h	10 min	no	yes
S4 E-F	*hsflp* $^{[122]}$ */ UAS-fkh-3xHA; + / zip>GFP; Act5C.GAL4 (FRT.CD2), UAS-RFP /+*	30 h	10 min	no	yes
S4 G-H	*UAS-fkh-3xHA / hsflp* $^{[122]}$*; + / +; act > y*$^{[+]}$ *> GAL4, UAS-GFP/+*	30 h	10 min	no	yes
S4 I-J	*hsflp* $^{[122]}$ */ UAS-fkh-3xHA; + / +; Act5C.GAL4 (FRT.CD2), UAS-RFP / sqh>MoeGFP*	30 h	15 min	no	yes
S4 K-M	*UAS-fkh-3xHA / hsflp* $^{[122]}$*; +/ +; act > y*$^{[+]}$ *> GAL4, UAS-GFP/+*	30 h	10 min	no	yes
S4 N, P	*UAS-fkh-3xHA / hsflp* $^{[122]}$*; + / +; act > y*$^{[+]}$ *> GAL4, UAS-GFP/+*	30 h	10 min	yes	yes
S4 O, Q	*UAS-fkh-3xHA / hsflp* $^{[122]}$*; + / +; act > y*$^{[+]}$ *> GAL4, UAS-GFP/+*	30 h	15 min	yes	yes
	Figure S6				
S6 F	*hsflp*$^{[122]}$*/+ or Y; ubi <stop <GFP*^{nls}*, act5C <stop <lacZ*^{nls}*/Sp; Act5C <stop <GAL4, UAS-his2A::RFP/Dr*	30 h	7 min	24 h	no
S6 G	*hsflp*$^{[122]}$*/+ or Y; ubi <stop <GFP*^{nls}*, act5C <stop <lacZ*^{nls}*/Sp; Act5C <stop <GAL4, UAS-his2A::RFP/UAS-ci.HA.wt*	30 h	7 min	24 h	no

S6 J	*UAS-fkh-3xHA / hsflp* [122]*; + / +; act > y*[+] *> GAL4, UAS-GFP/UAS-dIAP*	30 h	7 min	no	no
S6 K	*UAS-fkh-3xHA / hsflp* [122]*; + / +; act > y*[+] *> GAL4, UAS-GFP/+*	30 h	7 min	no	no
S6 L	*UAS-fkh-3xHA / hsflp* [122]*; + / +; act > y*[+] *> GAL4, UAS-GFP/UAS-dIAP*	30 h	7 min	no	no
S6 M	*UAS-fkh-3xHA / hsflp* [122]*; + / +; act > y*[+] *> GAL4, UAS-GFP/UAS-dIAP*	30 h	30 min	no	no
S6 N	*UAS-fkh-3xHA / hsflp* [122]*; + / +; act > y*[+] *> GAL4, UAS-GFP/+*	30 h	30 min	no	no
S6 O	*hsflp* [122] */ + or Y; + / UAS-ey; act > y*[+] *> GAL4, UAS-GFP/ +*	30h	7 min	no	no
S6 P	*hsflp* [122] */ + or Y; + / UAS-ey; act > y*[+] *> GAL4, UAS-GFP/ +*	30h	30 min	no	no
	Movie S1				
	hsflp [122] */+; FRT42D ubi-mRFP / FRT42D Psc-Su(z)2*[XL26]*; +/+*	102 h	45 min	no	yes

Supplemental experimental procedures

Drosophila genetics

For detailed fly genotypes and heat-shock induction conditions please refer to Table S1. Briefly, all crosses were kept on standard media at 25°C. FLP/FRT and 'GAL4/UAS flip-out' overexpression experiments utilized heat-shock-driven expression of a flipase. The respective crosses were allowed to lay eggs for 72 h at 25°C followed by a heat-shock at 37°C for 40-60 min (FLP/FRT) or 5-25 min ('GAL4/UAS flip-out'). Larvae were dissected at wandering 3rd instar stage or as indicated (0 h, 30 h, 54 h or 102 h after heat-shock). To generate tightly staged larval populations for clone shape and actin intensity quantification experiments (Fig. 2 & 5), crosses were allowed to lay eggs for 7 h on grape plates. 1st instar larvae were transferred to standard media 24 h later (50 larvae per vial) and heat-shocked 72 h AEL at 37°C. For Tie-Dye experiments (Fig. 6), crosses were allowed to lay eggs on standard media for 24 h at 18°C to prevent early induction of flipase expression. 34 h after start of egg lay, vials were transferred to 25°C and 72 h after egg lay, larvae were subjected to a heat-shock at 37°C for 7 min. Larvae were dissected 30 h after heat-shock and analyzed as described below.

Immunohistochemistry and imaging

Imaginal discs were dissected and fixed in 4% formaldehyde/PBS for 18 min at room temperature. Washes and blocking were performed in PBS + 0.1 Triton X-100 (PBT) and PBT+5% normal goat serum, respectively. Discs were incubated with primary antibodies overnight at 4°C: guinea pig anti-Spaghetti-squash 1P (1:400, gift from Robert Ward), mouse anti-βPS-Integrin (1:10, DSHB, CF.6G11), rabbit anti-phospho-Ezrin/Radixin/Moesin (1:500, Cell Signaling, #3149), mouse-anti Rho1 (1:100, DSHB, p1D9), mouse anti-wingless (1:100, DSHB, 4D4), mouse anti-patched (1:20, DSHB, Apa1), rabbit anti-Cleaved Drosophila Dcp-1 (1:250) (Cell Signaling, #9578). Discs were counterstained with DAPI (0.25 ng/μl, Sigma), Phalloidin (Alexa Fluor 488 and Alexa Fluor 647 phalloidin,1:100, Molecular Probes, or Phalloidin-TRITC, 1:400, Sigma). Secondary antibodies (coupled to Alexa Fluorophores, Molecular Probes) were incubated for 2 h at room temperature. Discs were mounted using Molecular Probes Antifade Reagents (#S2828). To prevent squeezing of samples by coverslips, two stripes of double-sided tape (Tesa, #05338) were placed on the slide. Samples were imaged using a Leica TCS SP5 confocal microscope equipped with HCX PL APO Lambda Blue 20x (NA 0.7) and HCX PL APO Lambda Blue 63x (NA 1.4) lenses. Images were processed and analyzed using Fiji (ImageJ 1.48b).

Collagenase treatment

Wing imaginal discs were incubated for 12 min in 200 μl - 400 μl of Collagenase (CLSPA; Worthington Biochemical Corp, 1000 u/ml + 2 mM $CaCl_2$ and 2mM $MgCl_2$). Discs were immediately fixed for 18 min in 4% PFA/PBS and processed as described above.

Image Analysis and Quantification using Fiji

General information on imaging conditions

For all clone shape quantifications at least 3 imaginal discs were analyzed. To obtain an overview of the pouch, a stack was imaged using a 63x objective (zoom 1, xy 0.24 μm, z 1.51 - 2.98 μm). Subsequently, selected regions were imaged at higher magnification (zoom 3, xy 0.08 μm, zoom 2.5 xy 0.096 μm, z 0.42 - 1.0 μm). Channels were merged in FIJI and cropped stacks representing individual clones were used for nuclei counting, clone volume and circularity measurements. For Tie-Dye experiments, image stacks of the pouch and hinge region (20x objective, xy 0.45 - 0.54 μm, z 2.01 - 2.18 μm) were generated and analyzed. For Dcp-1 quantifications, image stack using a 63x objective (zoom 1, xy 0.24 μm, z 2.01 - 3.48 μm) were analyzed.

General information on statistical analysis of imaging data

Every data set was checked for normality of distribution and homogeneity of variances by applying Shapiro's and Bartlett's test, respectively. The α value for each analysis was set to 0.01 ($\alpha = 0.01$). Wilcoxon Signed-Rank test (WSR), Wilcoxon-Mann-Whitney test (WMW) and Welch's t-test were applied as indicated in figure legends.

Quantification of actin intensities (Fig. 2)

Actin intensities were measured on reconstructed xz-cross-section (reslice tool, 1 μm spacing, top and left reslice) from zoom 1 image stacks. In overlay stacks of phalloidin and clonal marker, the section at a vertical position through the center of radially symmetric clones was identified. Apical junctions and lateral surfaces were selected and average fluorescence intensity measured using the 'measure' tool. For each clone, the two lateral MWI interfaces, up to 8 cell surfaces inside the clone and up to 15 cell surfaces outside the clone, near the MWI, were measured. Data points were averaged to obtain 1 value for wt/wt, wt/mis and mis/mis interfaces for each clone. These values were then normalized to the average mean intensity of actin staining on wt/wt interfaces within an experimental series.

Quantification of clone volumes (Fig. 5)

To determine volumes of individual clones, the GFP-signal defining the clone volume was used to generate a quantification mask employing the 'Threshold' (settings: default, stack histogram, dark background, between 5-30) and 'Remove outlier' (settings: black and white pixel removal with radii 0.5-2 at a threshold of 50) function of Fiji. The resulting binary mask was used to measure clone area in each section using the 'Analyze particles' function (settings: size (micron2̂): usually 10-Infinity for zoom 3 images and 5-Infinity for zoom 1 images, Circularity 0.00-1.00). To control if the clone area was well defined, the stack displaying the outlines of measured particles was merged with the original GFP stack and manually validated. Particle area measurements in all sections for each clone were summed up and multiplied with z-stack step size to obtain clone volumes.

Quantification of cell number and average cell volumes (Fig. 5)

Nuclei counts were obtained using reslices of zoom 3 image stacks. In an overlay stack between DAPI and clonal marker, nuclei were tracked through each slice and counted with the help of the text tool of Fiji. An average cell volume for each experimental condition was calculated by dividing the clone volume obtained above by nuclear counts for each individual clone. Average cell volumes obtained in zoom 3 images were used to extrapolate cell numbers from the GFP-volume measured for additional clones imaged at zoom 1 for the same experimental conditions.

Circularity measurements (Fig. 3)

To calculate circularity of *fkh*-overexpressing and neutral wild type clones, we used image stacks of discs that were heat-shocked for 10min and analyzed 30 h and 54 h later, as well as disc heat-shock for 25 min and analyzed 30 h after clone induction. A section at exactly a third (for *fkh*-expressing clones) and a fifth (for *Rho*^{V14}-expressing clones) of the disc height from the most basal section was identified and used for tracing a clone outline with the help of the polygon tool in Fiji. Area and perimeter of this region were measured and circularity was defined using the following equation:

$$C = 4\pi \frac{\text{Area}}{\text{Perimeter}^2}. \tag{1}$$

Quantification of wild type cell volumes

To quantify cell volumes and aspect ratios of wild type cells we analyzed stage-matched wild type imaginal discs derived from a neutral control cross or wild type cells next to *fkh*-overexpressing cells. Based on a phalloidin counterstain, 2 regions within the same stack were chosen for analysis: 15-25 wild type cells with (1) small apical cell areas and (2) big apical areas. This approach was chosen to reflect the endogenous diversity of cell shapes in imaginal discs. The height of cells was defined in a reslice and multiplied by the apical area of all selected cells. An average wild type cell volume was obtained by dividing the resulting volume through the number of selected cells.

Experimental clone shape analysis (Fig. 5)

To quantify experimental clone shapes and to compare them to computational simulations, we extracted characteristic shape coordinates for each clone. Precision of coordinate selection was verified between zoom 3 and zoom 1 image stacks. Zoom 1 image data was then used for further analysis because more clones could be sampled. Coordinates were defined using the Fiji Point picker tool. From each coordinate set, 4 characteristic quantities were extracted using MATLAB. First the apical surface of the wild type tissue was identified by the least-square fit of a linear function through all apical wild type points. Tissue height h was defined as the average distance of all basal wild type points to the apical surface. The absolute apical indentation u_a for each clone was defined as distance of the apical midpoint of the cyst from the apical wild type surface. Analogously, the basal deformation u_b was defined as the distance of the basal clone midpoint to a straight line fitted through basal wild type points. The apical and the basal width w_a and w_b are defined as the distance between the left and right clone interfaces apically and basally, respectively. As final step the measurements were normalized with the height h of the surrounding tissue, to obtain relative tissue deformations. For each clone, these normalised quantities were obtained in two perpendicular cross

sections and then averaged, to account for small deviations from a rotationally symmetric clone shape. These relative deformations u_a/h, u_b/h, w_a/h and w_b/h are plotted as a function of the clone size in Fig. 4 for the optimal parameter set and in Fig. S5 for variations around this set.

Tie-Dye clone size frequency - *fkh* and *fkh*, *dIAP* (Fig. 6)

Imaginal disc of all Tie-Dye genotypes were counterstained with DAPI and phalloidin and were processed in Fiji as follows: Peripodial membrane clones were excluded from the analysis and deleted in both GFP and RFP channels manually using the polygon and clear function. Since a majority of the Tie-Dye signal in the notum derives from adepithelial cells, the ROI set for subsequent analysis only considered the pouch and hinge regions of the disc. To identify the optimal fluorescence threshold for RFP and GFP masks, different thresholds were tested. This was done by choosing a threshold, removing outliers and generating outlines of the resulting mask. Mask outlines were merged with original images and checked for visual match with GFP and RFP borders. GFP and RFP thresholds were determined separately for each disc, but were validated by optimal fit and similar volume measurements for clones that express both GFP and RFP.

We decided to exclude GFP/RFP double-labeled clones from our final analysis because small differences in GFP and RFP masks would have caused us to systematically underestimate their size. We therefore only selected GFP or RFP positive clones by subtracting the mask of GFP from that of RFP and vice versa using the image calculator tool. In the resulting masks (GFP only, RFP only) outliers were removed by a series of 'Remove outlier' commands (settings: black and white pixel removal with radii 0.5-3 at a threshold of 50). Mask holes were filled using the 'Fill holes' tool.

The resulting GFP only and RFP only masks were analyzed using the 3D object counter (Version V2.0, Threshold 128, No exclusion of size or edges, Maps to show: Objects, Results table to show: Summary, Statistics). Volume measurements were transferred to Excel. 3-4 small clones per disc where selected from the object map, identified in original image and nuclei were counted for each clone. The clone volume as determined by the 3D analysis was divided by the number of nuclei to obtain an average nuclei volume for each genotype.

GFP and RFP volumes below an empirically determined minimum cell volume for each genotype were excluded from the analysis. All remaining data points were binned into multiples of empirically determined average cell volumes for each genotype. Relative loss of RFP clones was calculated for each chosen bin: (RFP clone count - GFP clone count) / GFP clone count within the analyzed bin.

Clone size quantification in *ci*-expressing Tie-Dye discs (Fig. S6)

ci-expressing and control Tie-Dye wing imaginal discs were counterstained with DAPI and Patched (Ptc) to mark the anterior/posterior compartment boundary. Image stacks were processed and analyzed in Fiji as follows: ROIs for the total pouch, the anterior and posterior compartment were generated on maximum projections of the Ptc staining. Maximum projections of GFP and RFP image substacks that included all clones in the pouch were created and segmentation masks using the threshold function followed by a series of 'Remove outlier' commands were established. GFP positive clones were selected by subtracting the RFP mask from the GFP mask using the image calculator tool. GFP and RFP clone areas in the whole pouch, the posterior and the anterior compartment were measured using the 'Analyze particle' function within previously defined ROIs.

Dcp-1 volume quantification (Fig. 6)

Imaginal disc carrying regular GAL4/UAS flip-out clones expressing *fkh* or *fkh*, *dIAP* were stained for Dcp-1, DAPI and phalloidin. Images were processed in Fiji as follows: Peripodial membrane clones were excluded from the analysis and manually deleted (GFP channel) using the polygon and clear function. Each image plane was classified in background, clone and Dcp-1 signal using the trainable Weka segmentation tool and segmentation holes were filled using the 'Fill Hole' function. The quality of the segmentation was verified visually. Dcp-1 signal that was not part of a flip-out clone was removed by combining Dcp-1 and GFP masks with a logical AND operation. Segmented image planes were used to reconstruct 3D objects of clonal and Dcp-1 stainings. For each clone, the total volume, the volume of all included Dcp-1 sites was determined. All 3D operations were performed with custom macros using the API of the 3D ImageJ Suite. For defining the average cell volume, 3-4 clones were selected per disc, nuclei within the clones were counted and divided by clone volume. GFP volumes below an empirically determined minimum cell volume for each genotype were excluded from the analysis. All remaining data points were binned into multiples of empirically determined average cell volumes for each genotype.

Modeling procedures

A 3D Vertex Model for simple epithelia

In this section we introduce and motivate a 3D Vertex Model for epithelial mechanics, which is then applied to quantitatively understand the formation of cysts in the *Drosophila* imaginal wing disc as a function of the distribution of forces generated inside the disc. Our 3D Vertex Model can be seen as a generalisation of the 2D vertex model [S3], where both apical and basal cell surfaces are taken into account, and the epithelium can deform in 3 dimensions.
We start by describing in detail how the epithelium is represented by a set of vertices, after which we show how internal and external forces are included in the vertex model description. In the last parts of the introduction we describe how topological transitions are implemented in our 3D Vertex Model and we provide details on how the simulations were carried out.

Geometry of the 3D Vertex Model

In the 3D Vertex Model the tissue is represented by a set of vertices. We distinguish a set of apical and basal vertices with positions $\mathbf{x}_a$ and $\mathbf{x}_b$, associated with the epithelium's apical and basal surfaces. Vertices are connected by a network of apical, basal and lateral bonds which represent the cell outlines. The apical and basal vertices and bond networks are taken to have the same topology, but can have different positions (Fig.3). Lateral bonds therefore simply connect each apical vertex to its basal counterpart.
Each cell α is represented by a set of M apical and basal vertices $\mathbf{x}_a^{\alpha,i}$ and $\mathbf{x}_b^{\alpha,i}(i = 1...M)$ and their associated bonds. Because vertices constituting a surface are not necessarily coplanar, the surface enclosing the cell has to be defined. Each interface k is defined by a subset of vertices $\mathbf{x}_1^k, \mathbf{x}_2^k, ..., \mathbf{x}_N^k$. The contour line C of the surface is then given by the path ($\mathbf{x}_1^k - \mathbf{x}_2^k - ... - \mathbf{x}_N^k - \mathbf{x}_1^k$) and the associated centre of mass $\mathbf{c}^k$ is defined as:

$$\mathbf{c}^k = \frac{1}{L}\int_C \mathbf{r}(s)ds \tag{2}$$

$$= \frac{1}{\sum_i |\mathbf{x}_i^k - \mathbf{x}_{i+1}^k|}\sum_i |\mathbf{x}_i^k - \mathbf{x}_{i+1}^k|\frac{\mathbf{x}_i^k + \mathbf{x}_{i+1}^k}{2}, \tag{3}$$

where $\mathbf{r}(s)$ is a vector pointing on the contour C, s is an Euclidean coordinate going along the contour C, and the sum over vertices i, j is performed on vertices belonging to the interface k. We then use the center of mass point $\mathbf{c}^k$ to generate a triangulation of the interface k, by joining the center of mass $\mathbf{c}^k$ with all vertices belonging to the interface. Each cell is therefore enclosed by a set of planar triangles (Fig.3C) and the triangulation is used to compute cell volume as well as the apical, basal and lateral surface areas.

Mechanical forces in the 3D Vertex Model

In the vertex model forces act to displace vertices that describe the epithelium. These forces are derived from a virtual work, based on the tensions generated inside the epithelium and external mechanical constraints. To emphasise the different contributions, the virtual work δW is written as the sum of the work exerted within

the epithelium δW_i and outside the epithelium δW_e:

$$\delta W = \delta W_i + \delta W_e. \tag{4}$$

The details of these contributions are given in the following sections.

Internal forces

The internal virtual work is written

$$\delta W_i = \sum_k T_k \delta A_k + \sum_{i,j} \Lambda_{ij} \delta l_{ij} - \sum_\alpha P_\alpha \delta V_\alpha, \tag{5}$$

where the indices i, j label vertices, the index k labels interfaces, and the index α labels cells. The volume of cell α, the surface area of interface k and the length of the bond joining the vertices i and j are denoted V_α, A_k, and l_{ij} respectively. The pressure acting in cell α is denoted P_α, the surface tension on interface k, T_k, and the line tension acting on the bond $\langle i, j \rangle$, Λ_{ij}. Surface tensions T_k and line tensions Λ_{ij} are thought to arise from the actomyosin cortical cytoskeleton. The contractility of myosin molecular motors in the surface actin network leads to the establishment of a surface tension [S4]. In general the tensions generated in surfaces and along lines can depend on the area of the surface or the length of the actin cable (i.e. $T_k = T_k(A_k)$, $\Lambda_{ij} = \Lambda_{ij}(l_{ij})$). For cyst simulations, we assume that all surfaces have a constant tension if their area is larger than a preset threshold, but if further constricted they exhibit a linearly elastic behaviour towards a preferred area value:

$$T_k(A_k) = \begin{cases} T_k^0 & \text{for } A_k > A_k^0 \\ T_k^* + \frac{A_k}{A_k^0}(T_k^0 - T_k^*) & \text{for } 0 \leq A_k < A_k^0. \end{cases} \tag{6}$$

This choice is motivated by the experimental observation that apical surfaces vanish rarely completely during cyst formation. Only apical cell surfaces were assumed to experience this elasticity and for them we chose in simulations $A_k^0 = \bar{A}/2$ and $T_k^* = -T_k^0$, with $\bar{A}$ being the average apical surface area of wildtype cells. Line tensions are assumed to be constant and independent of the length of the bond.
Cells are assumed to be able to maintain a preferred volume. The cell pressure P_α is taken to be linear in the deviation of the volume from the preferred volume

$$P_\alpha = -K_\alpha(V_\alpha - V_\alpha^0), \tag{7}$$

where V_α^0 is the preferred cell volume of cell α and the proportionality constant K_α is its bulk elastic modulus. In this study, K_α is chosen large enough that cells are close to incompressibility, with a relative deviation of the cells' volume from their preferred volume smaller than 0.001.

External forces acting on the epithelium

We take into account two additional forces: an external in-plane surface tension constraining the area of the tissue, and a term representing tissue attachment to the extracellular matrix (ECM). The ECM is considered

to be a flat surface located at $z = 0$. The external virtual work then reads

$$\delta W_e = \sum_v k_v x_v \delta x_v - T_{\text{ext}} \delta A^{tot}, \tag{8}$$

where the distance of a vertex v to the closest ECM point is denoted x_v, and the total tissue surface area is denoted A^{tot}. The spring modulus k_v represents elastic bonds attaching the tissue to the extracellular matrix, and it is non-zero only for basal vertices as the apical tissue surface is not connected to the ECM. In the wing disc, experimental evidence indicates that the tissue is under compression, $T_{\text{ext}} < 0$. The compression forces possibly arise from global constraints imposed by the ECM, which surrounds the wing disc. Indeed, removal of the ECM leads to area expansion of the tissue (Fig.S4D-G).

Forces acting on vertices

The force $\mathbf{F}_i$ that acts on vertex $\mathbf{x}_i$ can then be obtained by taking the derivative of the virtual work with respect to the vertex position (Fig. 3A):

$$\mathbf{F}_i = -\frac{\partial W}{\partial \mathbf{x}_i}. \tag{9}$$

Note that the centers of mass of the surfaces are not taken as degrees of freedom (cf. Eq. 3), but have to be considered in the calculation of the forces acting on the single vertices. To clarify this, we note that the virtual work introduced in Eq. 4 is in general a function of the positions of the vertices and centers of mass, $\delta\bar{W}(\mathbf{x}_i, \mathbf{c}_i)$. As the centers of mass depend on vertices positions through Eq. 3, the virtual work in Eq. 9 can be obtained from

$$\delta W(\mathbf{x}_i) = \delta\bar{W}(\mathbf{x}_i, \mathbf{c}_k(\mathbf{x}_i)), \tag{10}$$

such that the force on any vertex i can be obtained as follows:

$$\mathbf{F}_i = -\frac{\partial \bar{W}}{\partial \mathbf{x}_i} - \sum_{k=1}^{M} \frac{\partial \bar{W}}{\partial \mathbf{c}^k} \frac{\partial \mathbf{c}^k}{\partial \mathbf{x}_i}. \tag{11}$$

The tissue is in equilibrium if the forces on all vertices i vanish:

$$\mathbf{F}_i = \mathbf{0}. \tag{12}$$

Topological transitions

In addition to changes in the position of vertices the tissue can undergo T_1 topological transitions, in the course of which cells exchange neighbours. Transitions occur through the shrinkage of the interface in between two cells and the expansion of a new interface between two cells which were previously not in contact.

We impose here that T_1 topological transitions must occur both on the apical and the basal side, such that both networks maintain the same topology. In the implementation of the 3D Vertex Model, a T_1 transition occurs if the apical and the basal length of an edge e fall under the threshold length l^{T1} and the forces acting on the vertices of the edge tend to further reduce the apical and basal length of the edge (Fig. S4A). In the exemplary illustration the edge e, common to cells 2 and 4, is then replaced by a single fourfold vertex. This

new vertex can be replaced by a new bond, connecting the cells 2 and 4 which were previously unconnected. To decide if this topological transition is performed, a hypothetic new bond $e^{1,3}$ is introduced between the two cells 1 and 3, with the direction of the apical/basal edge obtained by connecting the apical/basal cell centres of the other two cells. The apical and basal cell centres of cell α are denoted $\mathbf{M}_a^\alpha$ and $\mathbf{M}_b^\alpha$ respectively. We denote $\mathbf{F}_a^2$ and $\mathbf{F}_a^4$ the forces acting on the apical vertices of the hypothetic edge e which are part of cell 2 and 4, and $\mathbf{F}_b^2$ and $\mathbf{F}_b^4$ the respective forces on the basal vertices. The separating forces for the hypothetic edge $e^{1,3}$ are given by the projection of the force on the opening direction:

$$f_a^{1,3} = (\mathbf{F}_a^2 - \mathbf{F}_a^4) \cdot \frac{\mathbf{M}_a^2 - \mathbf{M}_a^4}{|\mathbf{M}_a^2 - \mathbf{M}_a^4|} \tag{13}$$

$$f_b^{1,3} = (\mathbf{F}_b^2 - \mathbf{F}_b^4) \cdot \frac{\mathbf{M}_a^2 - \mathbf{M}_a^4}{|\mathbf{M}_a^2 - \mathbf{M}_a^4|} \tag{14}$$

$$f^{1,3} = f_a^{1,3} + f_b^{1,3}. \tag{15}$$

The hypothetic edge $e^{1,3}$ is allowed to open if both apical and basal opening forces are positive $f_a^{1,3} > 0, f_b^{1,3} > 0$. The newly established edge is assigned the initial apical and basal lengths l_a^{new} and l_b^{new}. The topology remains unchanged and the fourfold vertex is conserved if the formation of the new edge is unfavourable.

3D Vertex Model implementation for the numerical study of cyst formation

Periodic boundary conditions

In the simulation of the wing disc in the 3D Vertex Model, the epithelium is assumed to have periodic boundary conditions in x and y directions. A rectangular box with sizes L_x and L_y is therefore used for simulations (Fig.S4B). The size of the box is not fixed, and the positions of all vertices in the box are affinely rescaled when the lengths L_x and L_y are modified: under a change of tissue size $L_x \to L_x + \delta L_x, L_y \to L_y + \delta L_y$ every position $\mathbf{P}$ changes according to

$$\mathbf{P} \to \begin{pmatrix} \frac{L_x+\delta L_x}{L_x} & 0 & 0 \\ 0 & \frac{L_y+\delta L_y}{L_y} & 0 \\ 0 & 0 & 1 \end{pmatrix} \mathbf{P}. \tag{16}$$

The equilibrium system size (Fig.S4C) is obtained through the force balance equations:

$$\frac{\partial W}{\partial L_x} = \frac{\partial W_i(L_x, L_y)}{\partial L_x} - T_{\text{ext}} L_y = 0 \tag{17}$$

$$\frac{\partial W}{\partial L_y} = \frac{\partial W_i(L_x, L_y)}{\partial L_y} - T_{\text{ext}} L_x = 0. \tag{18}$$

Starting configurations

Simulations were performed with a tissue containing 2000 cells. To obtain different starting configurations of the tissue, random tissues were created by performing a 2D Voronoi tessellation of randomly distributed points in a periodic box of size $L_x^0 \times L_y^0$. Apical and basal vertices were then assigned the same x and y coordinates, but different z coordinates (Fig.S4B). This random initial configuration was then relaxed to an equilibrium shape by minimising the work function with respect to system size, vertex positions and topology

(Fig.S4C). For each set of examined mechanical parameters and each cyst size, we ran simulations with 15 different starting configurations of the tissue.

Minimisation algorithm

For the choice of the virtual work function we use for cyst simulations, δW can be integrated to yield

$$W = \sum_{\alpha} -P_\alpha V_\alpha + \sum_k W_k(A_k) + \sum_{ij} \Lambda_{ij} l_{ij} + \sum_v \frac{k_v}{2} d_v^2 - T_{\text{ext}} A^{tot}, \tag{19}$$

where $W_k(A_k) = \int_0^{A_k} T_k(\tilde{A}_k) d\tilde{A}_k$. For a fixed topology of the network of bonds and vertices, the work W can be minimised with respect to the position of the vertices and the system size L_x, L_y to find mechanical equilibrium configurations of the network. This is numerically done in a C++ implementation of the Polak-Ribiére conjugate gradient algorithm [S5].

Clone specification

An initial clone consisting of N mutant cells is created by assigning mutant properties to the N cells closest to an arbitrarily selected point in the relaxed homogeneous tissue (Fig.4A). To account for interface contraction, changes are applied to apical line and/or lateral surface tension at the interfaces between the two different cell populations, whereas all other mechanical properties of the mutant cells remain unchanged (Fig.4D-E). To account for increased bulk contraction in simulations, the lateral interface tension between mutant cells has been changed in comparison to the lateral surface tension between wild type cells, with the interface tension between mutant and wild type cells being the arithmetic mean of the two (Fig.4B-C).

Parameter search

In this section, we describe the parameter search procedure that was performed to reproduce the experimental dependency of four clone geometrical measurements on the number of cells in the clone N_c (Fig. 5C-D). Simulations involve 10 independent parameters listed in Table 1. Renormalising all lengths with the reference length scale $l_0 = (V_0)^{1/3}$ and line and surface tensions by the surface tensions T_l leaves 8 remaining unknown parameters.
To account for the shape of the wild type tissue, two additional constraints were introduced that we now describe. Eq. 67 is then used to obtain an relation between the average tissue aspect ratio $\beta = \langle h/\sqrt{A} \rangle$ and the external tension acting on the tissue T_{ext}:

$$\beta \simeq \frac{\gamma_a(\beta) + \gamma_b(\beta) - T_{\text{ext}}}{\frac{3^{1/4}}{\sqrt{2}} T_l}. \tag{20}$$

γ_a and γ_b denote the total tensions acting in the apical and basal surfaces of the tissue, and the corresponding expressions are given in Equations 68 and 69. Note that Eq. 67 was obtained for a regular hexagonal packing, and we assume it holds approximately true for the disordered packing considered here.
The average wild type aspect ratio of the pouch in the presence of the ECM was found to be $\beta^{wt} \simeq 16.2$ (Fig.S4F). The ECM possibly exerts an external tension T_{ext} on the tissue, and to estimate this tension the relaxation of the tissue after the removal of the ECM was analysed (Fig.S4D-G). Treatment of the wing discs

with collagenase led to significant ECM removal (Fig.S4D-E) and to the subsequent relaxation of the tissue shape on timescales of minutes, in the course of which the pouch flattened and the cells assumed a new aspect ratio $\beta^{coll} \simeq 11.6$ (Fig.S4F). Assuming that collagenase application only results in removal of mechanical constraints exerted by the ECM, this observation indicates the epithelium is under compression. Denoting T_{ext}^{wt} the compression due to the ECM, and T_{ext}^{coll} the remaining compression after collagenase treatment and removal of the ECM, the following two constraints enforce the tissue to have the right aspect ratio in both cases:

$$\beta^{wt} = \frac{\gamma_a(\beta^{wt}) + \gamma_b(\beta^{wt}) - T_{\text{ext}}^{wt}}{\frac{3^{1/4}}{\sqrt{2}} T_l} \tag{21}$$

$$\beta^{coll} = \frac{\gamma_a(\beta^{coll}) + \gamma_b(\beta^{coll}) - T_{\text{ext}}^{coll}}{\frac{3^{1/4}}{\sqrt{2}} T_l}. \tag{22}$$

Replacing the experimental values of β^{wt} and β^{coll} in equalities 21 and 22 imposes two constraints on the model parameters. Under these additional two constraints, a parameter search was performed to adjust the 8 last parameters listed in Table 1. We verified that the apical line tension and lateral surface tension around the clone must both be increased to explain experimental observations (Fig. S5C-G), we therefore assume here for simplicity that they are increased by the same factor. For each set of tested parameters 450 simulations with 30 different cyst sizes and 15 different initial tissue configurations were run, from which averages and standard error of the mean of the resulting cyst deformations were obtained, as shown in Fig. 5C-D. The corresponding curves of cyst deformation as a function of the number of cells within the cyst were generated for ~200 different parameter sets. The set of parameters given in Table 1 was found to give rise to equilibrium shapes that closely resemble the experimental data. In order to identify the key mechanical parameters controlling the deformations, simulations were run where only single parameters were changed in comparison to the preferred set of parameters given in Table 1. The results are shown and described in Supplementary Figure S5.

**description**	**parameter**	**value**
cell volume	V_0/l_0^3	1
lateral surface tension	T_l/T_l	1.0
lateral surface tension around clone	T_l^c/T_l	3.0
apical surface tension	T_a/T_l	3.1
basal surface tension	T_b/T_l	6.95
external compression	T_{ext}/T_l	-4.2
apical line tension	$\Lambda_a/(l_0 T_l)$	0.18
apical line tension around clone	$\Lambda_a^c/(l_0 T_l)$	0.53
basal line tension	$\Lambda_b/(l_0 T_l)$	0.18
stiffness of ECM attachment	k_v/T_l	5.0

Table 1: List of parameters obtained from comparison of the vertex model to clone shape measurements as a function of clone size.

Description of cyst formation with a continuum theory

In this section an alternative modeling approach is introduced where the epithelium is represented by a thin layer, coupled to a solid substrate by elastic links. The tissue mechanical properties are accounted for by a Helfrich mechanical free energy based on the curvature and applied tension ([S6, S7]). An additional term represents the elastic coupling of the tissue to the flat ECM. We use this continuum description to derive conditions for the stability of a flat, homogeneous tissue. We also study the buckling threshold of a clone subjected to compression due to an additional tension acting along its boundary. A similar question of budding of a domain under line tension has been discussed for lipid membranes in [S8]. For a classical introduction into the buckling of circular plates under a uniform load see for instance [S9, S10].

Parametrisation and the free energy

The tissue is described here as a thin 2D layer, where a point on the mid-surface representing the tissue **X** is parametrised in the Monge gauge (Fig.S4H)

$$\mathbf{X} = h(x, y)\mathbf{e}_z. \tag{23}$$

The tissue has a bending rigidity κ which penalizes deviation of the local curvature C from the preferred curvature C_0. In addition, an elastic material with elastic modulus k is assumed to connect the tissue to the underlying ECM, represented as a flat surface at position $h = 0$ (Fig.S4H). The corresponding mechanical work for a region of tissue S then reads

$$W = \int_S dA \left(\frac{\kappa}{2} C^2 - \kappa C_0 C + w_0(\rho) + \frac{k}{2} h^2 \right), \tag{24}$$

where $C = \frac{1}{R_1} + \frac{1}{R_2}$ denotes the mean curvature with R_1 and R_2 the two principal radii, ρ is the cell density, and w_0 is the mechanical energy density at zero curvature and zero height. In the limit of weak bending $|\nabla h| \ll 1$, the work function reads at second order in height deviation

$$W \approx W_0 + \int dA \left(\gamma \frac{(\nabla h)^2}{2} + \frac{\kappa}{2} (\Delta h)^2 - \kappa C_0 \Delta h + \frac{k}{2} h^2 \right) \tag{25}$$

where we have introduced the surface tension $\gamma = d(\rho^{-1} w_0)/d(\rho^{-1})$.

Linear stability of the flat, homogeneous tissue

The tissue mid plane position is described here in the 2D Monge gauge by the height function $h = h(t, \mathbf{x})$ where $\mathbf{x} = (x, y)$ describes the position in the plane. The tissue is defined on a disc centred at the origin with radius R, $\Omega(R) = \{\mathbf{x} : |\mathbf{x}| \leq R\}$. Introducing an effective friction coefficient $\alpha > 0$, the dynamical equation for the tissue height reads by differentiating Eq. 25 with respect to h:

$$\alpha \partial_t h(\mathbf{x}, t) = -\kappa \Delta^2 h(\mathbf{x}, t) + \gamma \Delta h - k h(\mathbf{x}, t) \tag{26}$$

with $\Delta = \partial_x^2 + \partial_y^2$ the Laplacian operator. If the tissue is periodic on the region $\Omega = [0, L] \times [0, L]$, the Fourier transform of $h(\mathbf{x}, t)$ on Ω is defined as:

$$\tilde{h}(\mathbf{q}, t) = \int_\Omega h(\mathbf{x}, t) e^{i\langle \mathbf{q}, \mathbf{x} \rangle} d\mathbf{x} \tag{27}$$

for $\mathbf{q} \in \tilde{\Omega} = [1/L, \infty] \times [1/L, \infty]$. Denoting $q = |\mathbf{q}|$, the dynamic equation (26) can be written in Fourier space:

$$\alpha \partial_t \tilde{h}(\mathbf{q}, t) = (-\kappa q^4 - \gamma q^2 - k)\tilde{h}(\mathbf{q}, t), \tag{28}$$

and therefore the criterion for mode $\mathbf{q}$ to be stable is

$$\kappa q^4 + \gamma q^2 + k > 0. \tag{29}$$

In the case of an infinite tissue all modes $q > 0$ are allowed, such that an infinite tissue is stable only for large enough tension γ:

$$\gamma > \gamma^* = -2\sqrt{\kappa k}. \tag{30}$$

The above criterion implies that a large tissue under compression ($\gamma < 0$) is stable only if the tissue is connected to the ECM with a nonzero attachment stiffness, $k \neq 0$.

Stability analysis of a clamped circular tissue region subjected to a contractile boundary

We consider here a stable tissue under tension $\gamma_0 > -2\sqrt{\kappa k}$. A circular region of the tissue is surrounded by a contractile boundary exerting a line tension Λ (Fig.5A, Fig.S4H). The resulting compression γ_c on the tissue circular region with radius R is then given by the law of Laplace:

$$\gamma_c = \gamma_0 - \frac{\Lambda}{R}. \tag{31}$$

For simplicity we consider that the circular region is clamped at its boundary. The dynamic equation for the shape of the tissue region is given by Eq. 26 with $\gamma = \gamma_c$. We assume rotational symmetry of the tissue region and denote r the radial coordinate. The clamped boundary conditions then read

$$h(R) = 0 \tag{32}$$

$$\partial_r h(R) = 0. \tag{33}$$

To study the stability of the circular tissue region, we make the following ansatz for $h(r, t)$:

$$h(r, t) = \tilde{h}(r) e^{st}. \tag{34}$$

We require that $Im(s) = 0$ and find that the tissue is unstable when a solution can be found for non-zero $\tilde{h}(r)$ and $s > 0$. Following this ansatz Eq. 26 can be rewritten

$$-\kappa\Delta_r^2\tilde{h}(r) + \gamma_c\Delta_r\tilde{h}(r) - (k + \alpha s)\tilde{h}(r) = 0, \tag{35}$$

where Δ_r is the Laplacian in polar coordinates with rotational symmetry

$$\Delta_r = \frac{\partial^2}{\partial r^2} + \frac{1}{r}\frac{\partial}{\partial r}. \tag{36}$$

The solution of Eq. 35 can be written

$$\tilde{h}(r, s) = AJ_0(\lambda_+ r) + BJ_0(\lambda_- r) + CY_0(\chi_+ r) + DY_0(\chi_- r), \tag{37}$$

where we have introduced 4 unknown constants A, B, C, D which have to be specified by the boundary conditions, and $J_0(x)$ and $Y_0(x)$ are Bessel functions of the first and second kind, respectively. Because $Y_0(x)$ diverges for $x \to 0$ and the height function must stay finite for $r \to 0$, it follows that $C = D = 0$. The two inverse length scales $\lambda_\pm > 0$ are solutions of the polynomial equation

$$\kappa\lambda_\pm^4 + \gamma_c\lambda_\pm^2 + (k + \alpha s) = 0, \tag{38}$$

which follows from the relation $\Delta_r J_0(\lambda r) = -\lambda^2 J_0(\lambda r)$. Solving Eq. 38, we find the following expressions for λ_+ and λ_-:

$$\lambda_\pm = \sqrt{-\frac{\gamma_c}{2\kappa} \pm \sqrt{\frac{\gamma_c^2}{4\kappa^2} - \frac{k + \alpha s}{\kappa}}}. \tag{39}$$

Imposing the boundary conditions (32)-(33) then results in the following two relations

$$AJ_0(\lambda_+ R) + BJ_0(\lambda_- R) = 0 \tag{40}$$

$$A\lambda_+ J_1(\lambda_+ R) + B\lambda_- J_1(\lambda_- R) = 0, \tag{41}$$

which have a non-zero solution for A and B provided that

$$F(s) = \lambda_- J_0(\lambda_+ R)J_1(\lambda_- R) - \lambda_+ J_0(\lambda_- R)J_1(\lambda_+ R) = 0. \tag{42}$$

where the dependency on s arises from λ_+ and λ_-. Solutions of Eq. 42 yields the rates of growth of perturbations of the shape of the circular region. Stability of the circular tissue region requires that all these solutions verify $Re(s) < 0$. The stability threshold of the clone can be found by solving the equation $F(0) = 0$. In doing so, a trivial solution $\lambda_+ = \lambda_-$ can be discarded as it results in a vanishing height profile. To obtain Figures S4K and S4L, we solve this equation numerically to obtain a phase diagram as a function of R and Λ.

We now give analytical expressions for the boundaries of the stability diagram in simple limits. We first note that in the limit of a large tissue region $R \to \infty$, the stability criterion converges to the stability threshold of

an infinite tissue, $\gamma_c > -2\sqrt{\kappa k}$ (Eq. 30). As a result, for large R the circular tissue region is unstable for

$$R < \frac{\Lambda}{\gamma_0 + 2\sqrt{k\kappa}}. \tag{43}$$

Subsequently we find the stability line in the limit where there is no elastic attachment to the ECM, $k = 0$. The stability threshold then reads simply

$$J_1\left(\sqrt{\frac{-\gamma_c}{\kappa}}R\right) = 0, \tag{44}$$

which has solution only for $\gamma_c < 0$, as the region is always stable for $\gamma_c > 0$. Denoting $j_{11} \simeq 3.832$ the first zero of the Bessel function J_1 and using Eq. 31, the condition for the circular region to be unstable can be rewritten

$$\gamma_0 R^2 - \Lambda R + j_{11}^2 \kappa < 0 \tag{45}$$

such that the sizes of unstable circular region are given for $k = 0$ by

$$\frac{\Lambda}{2\gamma_0} - \sqrt{\left(\frac{\Lambda}{2\gamma_0}\right)^2 - \frac{j_{11}^2\kappa}{\gamma_0}} < R < \frac{\Lambda}{2\gamma_0} + \sqrt{\left(\frac{\Lambda}{2\gamma_0}\right)^2 - \frac{j_{11}^2\kappa}{\gamma_0}} \qquad \text{for } \gamma_0 > 0 \tag{46}$$

$$R > \frac{j_{11}^2\kappa}{\Lambda} \qquad \text{for } \gamma_0 = 0 \tag{47}$$

$$R > \frac{\Lambda}{2\gamma_0} + \sqrt{\left(\frac{\Lambda}{2\gamma_0}\right)^2 - \frac{{j_{11}}^2\kappa}{\gamma_0}} \qquad \text{for } \gamma_0 < 0. \tag{48}$$

The lower limit for all values of γ_0 is $R > j_{11}^2\kappa/\Lambda$ for large $\Lambda \gg \sqrt{\kappa|\gamma_0|}$. An upper limit to stability exists only for positive tension $\gamma_0 > 0$; for negative tension $\gamma_0 < 0$ an infinite tissue is unstable, such that for $R \to \infty$ the circular region is always unstable when $k = 0$. The asymptotic stability criterion 43 indicates however that for $k > 0$ and $\gamma_0 < 0$, a region of large stable tissue sizes arises at large R when the attaching spring elasticity is large enough, $k > \gamma_0^2/(4\kappa)$.

Relation between the continuous model and the 3D Vertex Model

In this section the coarse grained parameters γ_0, κ, k and Λ of the continuum theory are related to the parameters of the 3D Vertex Model. The coarse grained tissue parameters are derived for a homogeneous tissue of identical cells arranged in a hexagonal packing. A similar approach is used in Ref. [S11]. We also do not discuss here anisotropic effects in the plane of the tissue. We take into account surface tensions on lateral, apical, and basal surfaces (T_l, T_a and T_b respectively), apical and basal line tensions (Λ_a, Λ_b respectively), elastic attachment of the vertices to the ECM (k_v) and the external tension acting on the tissue (T_{ext}). These parameters are taken here to be independent of the tissue geometry. The apical and basal areas of cell α are denoted A_α^a and A_α^b, the perimeters are denoted P_α^a and P_α^b, the volume is denoted V_α and the average Euclidean distance of the basal vertices to the ECM is denoted d_α. Following these assumptions, the mechanical energy W_Ω of a region of tissue Ω can be written as a sum of the effective free

energies of cells contained in this region of tissue

$$W_\Omega = \sum_{\alpha \in \Omega} \left(\frac{K_{3D}}{2}(V_\alpha - V^0)^2 + T^a A^a_\alpha + T^b A^b_\alpha + \frac{T^l}{2} A^l_\alpha + \frac{\Lambda^a}{2} P^a_\alpha + \frac{\Lambda^b}{2} P^b_\alpha + 2\frac{k_v}{2} d^2_\alpha \right). \tag{49}$$

where the factor 2 in the last term arises from the fact that each cell has on average 2 vertices. If all cells are assumed to have the same shape, the corresponding energy density $w = W_\Omega \rho / N_\Omega$ can be written:

$$w = \rho \left(\frac{K_{3D}}{2}(V - V^0)^2 + T^a A^a + T^b A^b + \frac{T^l}{2} A^l + \frac{\Lambda^a}{2} P^a + \frac{\Lambda^b}{2} P^b + k_v d^2 \right). \tag{50}$$

with N_Ω the number of cells within the piece of tissue Ω. We restrict ourselves here to cells with apical and basal surfaces being regular hexagons.The energy density $w(h, R_a, R_b)$ can then be reexpressed in terms of the cell height h and apical and basal radii R_a and R_b. The apical, basal and lateral surface areas, apical and basal perimeters and cell volume can indeed be related to h, R_a, R_b through the following relations:

$$A^a = \frac{3\sqrt{3}}{2} R_a^2 \tag{51}$$

$$A^b = \frac{3\sqrt{3}}{2} R_b^2 \tag{52}$$

$$A^l = 3\left(R_a + R_b\right)\sqrt{h^2 + \frac{3}{4}(R_a - R_b)^2} \tag{53}$$

$$P^a = 6R_a \tag{54}$$

$$P^b = 6R_b \tag{55}$$

$$V = \frac{\sqrt{3}}{2} h \left(R_a^2 + R_b^2 + R_a R_b\right). \tag{56}$$

We consider here the limit $K_{3D} \to \infty$ where the cell volume V is constrained to be equal to the reference volume V_0. The energy density $w(\rho, C, V_0)$ can then be written in terms of the cell density ρ, the total tissue curvature C and the reference volume V_0 through the following change of variables (Fig.S4I-J):

$$\rho = \frac{4}{(\sqrt{A^a} + \sqrt{A^b})^2} = \frac{8}{3\sqrt{3}(R_a + R_b)^2} \tag{57}$$

$$C = \frac{4(R_b - R_a)}{(R_a + R_b)h} \tag{58}$$

$$V_0 = \frac{\sqrt{3}}{2} h \left(R_a^2 + R_b^2 + R_a R_b\right). \tag{59}$$

For small curvature C, the energy density w can be expanded in power of the curvature C:

$$w(\rho, C, V_0) \simeq w_0(\rho, V_0) - \kappa C_0 C + \frac{\kappa}{2} C^2 + \frac{k}{2} d^2, \tag{60}$$

where the bending modulus of the tissue κ and the preferred curvature C_0 are functions of ρ, V_0 and the tissue line and surface tensions. In addition, we have intro cued the coarse-grained ECM elastic resistance $k = 2k_v\rho$. We obtain below the explicit expressions for these two mechanical quantities.

Bending modulus and preferred curvature

The effective bending modulus of the tissue κ is given by

$$\kappa = \left.\frac{\partial^2 w}{\partial C^2}\right|_{C=0} \tag{61}$$

$$= \frac{T^a + T^b}{8} V_0^2 \rho^2 + \frac{\sqrt{2}}{8 \cdot 3^{3/4}} T_l V_0 \sqrt{\rho} \left(\sqrt{3} - V_0^2 \rho^3\right) \tag{62}$$

and the preferred curvature of the tissue reads

$$C_0 = \frac{V_0\, \rho}{2\kappa} \left((T_a - T_b) + \frac{3^{1/4}}{\sqrt{2}} \sqrt{\rho} (\Lambda_a - \Lambda_b) \right). \tag{63}$$

Note that although κ can become negative for sufficiently large ρ, we have performed simulations here with parameters such that $\kappa > 0$.

Total in-plane surface tension

The effective surface tension γ exerted by the tissue depends on the apical and basal surface tensions, the apical and basal line tensions and the lateral surface tension:

$$\gamma = \frac{d(\rho^{-1} w_0)}{d(\rho^{-1})} \tag{64}$$

$$= T^a + T^b + \frac{3^{1/4}}{\sqrt{2}} \sqrt{\rho} (\Lambda^a + \Lambda^b) - \frac{3^{1/4}}{\sqrt{2}} \rho^{3/2} T_l V_0. \tag{65}$$

The effective surface tension exerted by the tissue has to be balanced by the external tension (i.e. $T_{\text{ext}} = \gamma$) for the tissue to be in mechanical equilibrium.
We rewrite the expression for the external tension by introducing the cell aspect ratio

$$\beta = \frac{h}{\sqrt{A}}. \tag{66}$$

With this definition, the total stress given in Eq. 65 can be rewritten

$$T_{\text{ext}} = \gamma_a(\beta) + \gamma_b(\beta) - \beta \frac{3^{1/4}}{\sqrt{2}} T_l, \tag{67}$$

where the total tensions exerted in the apical and the basal plane of the tissue γ_a and γ_b are respectively given by

$$\gamma_a(\beta) = T_a + \frac{3^{1/4}}{\sqrt{2}} \beta^{1/3} \frac{\Lambda_a}{l_0} \tag{68}$$

$$\gamma_b(\beta) = T_b + \frac{3^{1/4}}{\sqrt{2}} \beta^{1/3} \frac{\Lambda_b}{l_0}. \tag{69}$$

ECM elastic resistance

The coupling to the ECM in the 3D Vertex Model is represented by springs with spring constant k_v, that connect the basal vertices to the ECM. In a regular hexagonal packing every cell contains 6 vertices, which it each shares with two neighbouring cells, giving rise to the following coarse grained parameter of ECM attachment:

$$k = \frac{6}{3}\frac{k_v}{A_\alpha} = 2k_v\rho. \tag{70}$$

Clone boundary line tension

The excess tension due to the boundary effect around the clone can be due to an increase in apical and basal line tensions, as well as in lateral surface tension, Λ_a^e, Λ_b^e and T_l^e respectively, where the lateral surface tension has to be integrated over the cell height:

$$\Lambda = \Lambda_a^e + \Lambda_b^e + T_l^e V_0 \rho. \tag{71}$$

Critical buckling radii in the 3D Vertex Model

Using expressions for the coarse grained mechanical parameters as functions of the tensions generated in the 3D Vertex Model, an approximate critical buckling radii can be derived for simulations performed with the set of mechanical parameters given in Table 1. This can be done by using the stability criterion 42 obtained with the continuum theory and rewriting the continuum theory parameters in terms of the 3D Vertex Model parameters.

In order to relate the simulations and the analytical calculations, we also have to estimate the number of cells N in a circular clone with given radius R:

$$N = R^2\pi\rho. \tag{72}$$

Then we can use the experimental value of the aspect ratio of cells $\beta = h\sqrt{\rho} \approx 16.2$, to estimate the 2D cell density ρ for a given fixed cellular volume V_0:

$$\rho = \left(\frac{\beta}{V_0}\right)^{2/3}, \tag{73}$$

and using the relations 62, 65, 70 and 71, the values of the normalised effective mechanical parameters in simulations can be obtained:

$$\kappa/(T_l l_0^2) \simeq \ 0.34 \tag{74}$$

$$\gamma_0/T_l \simeq -4.2 \tag{75}$$

$$k/\left(\frac{T_l}{l_0^2}\right) \simeq \ 64.0 \tag{76}$$

$$\Lambda/(\beta^{2/3} l_0\, T_l + \Lambda_a) \simeq \ 2.0 \tag{77}$$

These normalised parameter values are used in Supplementary Figure S4L to plot phase spaces of predicted buckling instabilities for the simulations, assuming clamped boundaries of the clone.

Using the stability criterion given in Eq. 42, we show in Fig. S4L a phase diagram of buckling instability for a clone clamped at its boundary, with the resulting coarse grained parameters obtained above. For the increase in line tension corresponding to a 3-fold increase in apical line tension and lateral surface tension, the predicted range of buckling lies between about 3 and 106 cells. The maximum value of cyst formation is in qualitative agreement with the maximum size of observed cysts. Note however that the clamped boundary conditions used in this calculation are different from 3D Vertex Model simulations where the clone is embedded in the tissue.

References

[S1] Brandon D Bunker et al. ``The transcriptional response to tumorigenic polarity loss in Drosophila''. In: *eLife* 4 (2015), e03189.

[S2] Anne-Kathrin Classen et al. ``A tumor suppressor activity of Drosophila Polycomb genes mediated by JAK-STAT signaling." In: *Nature genetics* 41.10 (2009), pp. 1150–1155. ISSN: 1061-4036. DOI: 10.1038/ng.445. URL: http://dx.doi.org/10.1038/ng.445.

[S3] Reza Farhadifar et al. ``The influence of cell mechanics, cell-cell interactions, and proliferation on epithelial packing." In: *Current biology : CB* 17.24 (Dec. 2007), pp. 2095–104. ISSN: 0960-9822. DOI: 10.1016/j.cub.2007.11.049. URL: http://www.ncbi.nlm.nih.gov/pubmed/18082406.

[S4] Guillaume Salbreux, Guillaume Charras, and Ewa Paluch. ``Actin cortex mechanics and cellular morphogenesis''. In: *Trends in Cell Biology* 22.10 (2012), pp. 536–545. ISSN: 09628924. DOI: 10.1016/j.tcb.2012.07.001. arXiv: 84866882608. URL: http://dx.doi.org/10.1016/j.tcb.2012.07.001.

[S5] William H Press. *Numerical recipes 3rd edition: The art of scientific computing*. Cambridge university press, 2007.

[S6] W Helfrich. ``Elastic properties of lipid bilayers: theory and possible experiments." In: *Zeitschrift fur Naturforschung. Teil C: Biochemie, Biophysik, Biologie, Virologie* 28.11 (1973), pp. 693–703. ISSN: 0341-0471. DOI: 10.1002/mus.880040211.

[S7] Reinhard Lipowsky. ``The conformation of membranes." In: *Nature* 349 (1991). ISSN: 0028-0836. DOI: 10.1038/349475a0.

[S8] Reinhard Lipowsky. ``Budding of membranes induced by intramembrane domains''. In: *Journal de Physique II* 2.10 (1992), pp. 1825–1840. ISSN: 1155-4312. DOI: 10.1051/jp2:1992238.

[S9] Stephen Timoshenko and Serge Woinowksy-Krieger. *Theory of Plates and Shells*. McGraw-Hill Book Company, 1987.

[S10] Stephen P Timoshenko and James M Gere. *Theory of elastic stability, Engineering societies monographs*. 1961.

[S11] Edouard Hannezo, Jacques Prost, and Jean-Francois Joanny. ``Theory of epithelial sheet morphology in three dimensions." In: *Proceedings of the National Academy of Sciences of the United States of America* 111.1 (Jan. 2014), pp. 27–32. ISSN: 1091-6490. DOI: 10.1073/pnas.1312076111. URL: http://www.ncbi.nlm.nih.gov/pubmed/24367079.
