# Supplementary figures and images for: Interface Contractility between Differently Fated Cells Drives Cell Elimination and Cyst Formation
Source: Curr Biol. 2016 Mar 7;26(5):563–74. doi: 10.1016/j.cub.2015.12.063 (PMC5282066; doi:10.1016/j.cub.2015.12.063)

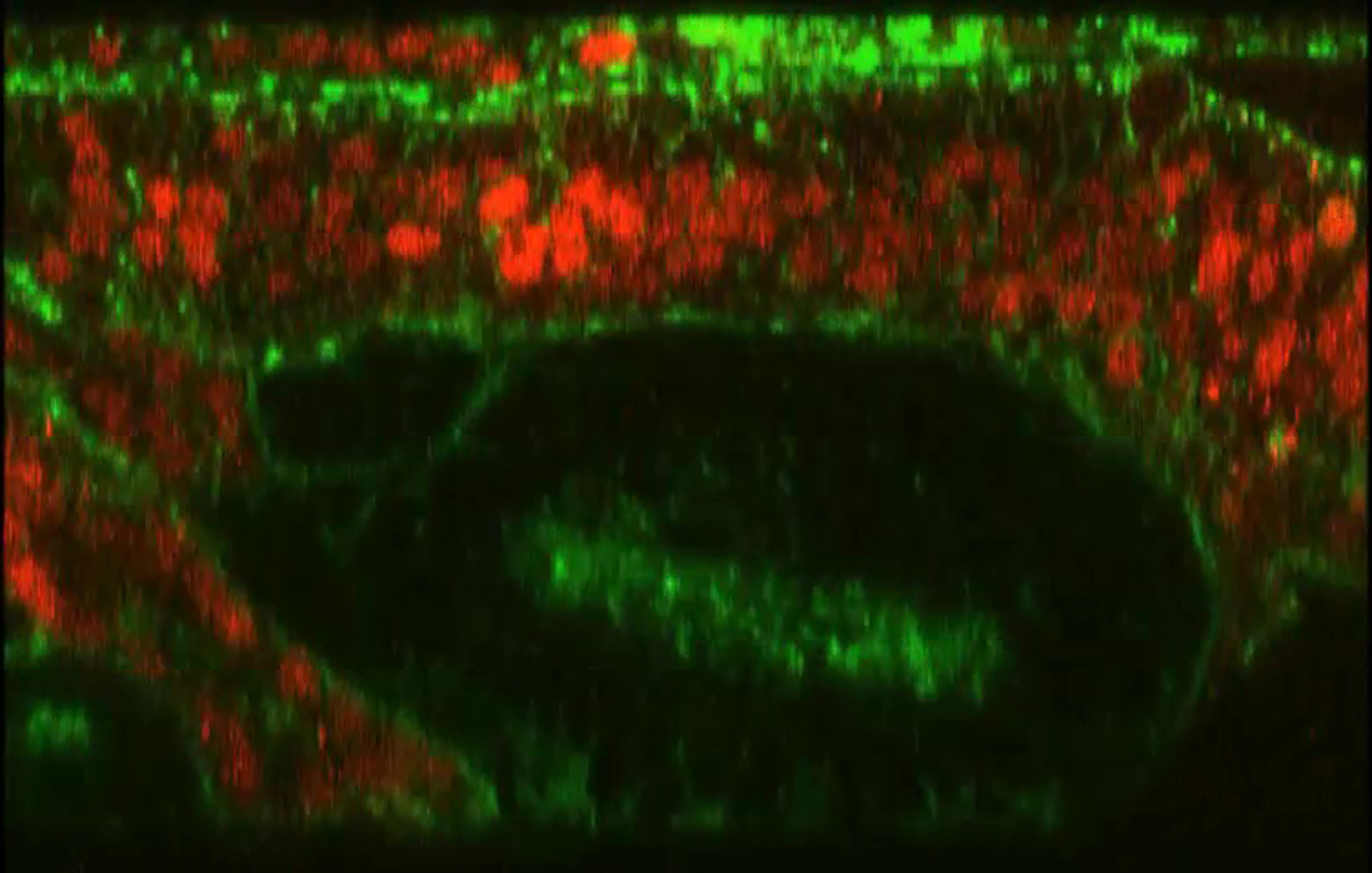

Supplement: Movie S1. Cyst Abscission at Late Stages of Cyst Formation — Wing imaginal disc containing Psc-Su(z)2XL26 clones at 102 hr after clone induction. Wild-type cells are marked by RFP (red) and cell outlines by phalloidin (green). A confocal xy stack was reconstructed into an xz cross-section movie. [file mmc2.jpg]
