# Supplementary material for: Interface Contractility between Differently Fated Cells Drives Cell Elimination and Cyst Formation
Source: Curr Biol. 2016 Mar 7;26(5):563–74. doi: 10.1016/j.cub.2015.12.063 (PMC5282066; doi:10.1016/j.cub.2015.12.063)
Supplement: Document S2. Article plus Supplemental Information [file mmc3.pdf]

Current Biology

Article

Interface Contractility between Differently Fated Cells Drives Cell Elimination and Cyst Formation

Graphical Abstract

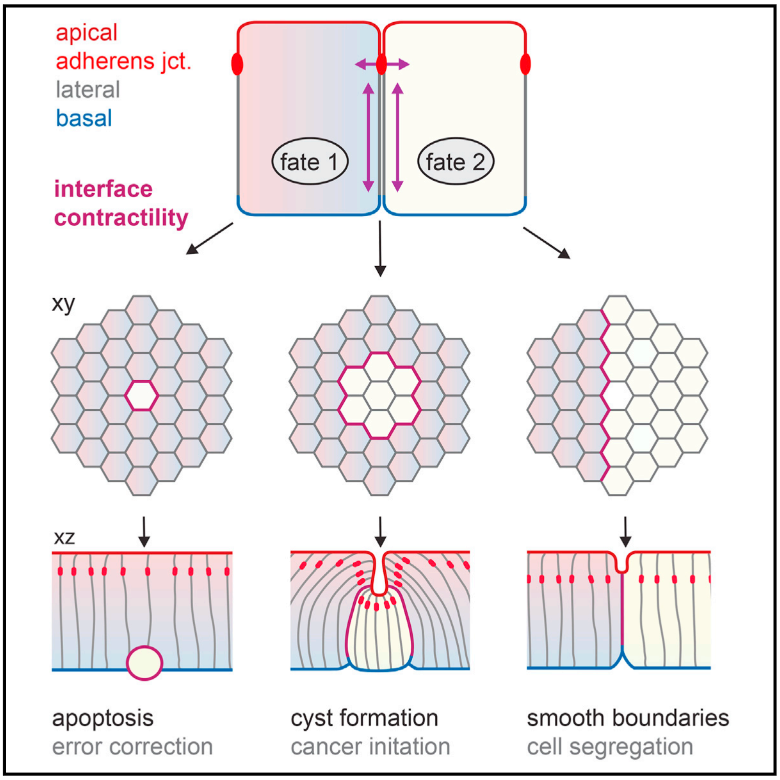

Highlights

- Different cell fates induce actomyosin contractility at shared cellular interfaces
- Our study implies cell-cluster-size-dependent consequences for tissue integrity
- Single cells are eliminated while intermediate-sized cell clusters form cysts
- Interface contractility may function in tissue homeostasis or tumor initiation

Authors

Christina Bielmeier, Silvanus Alt, Vanessa Weichselberger, ..., Frank Jülicher, Guillaume Salbreux, Anne-Kathrin Classen

Correspondence

guillaume.salbreux@crick.ac.uk (G.S.), classen@bio.lmu.de (A.-K.C.)

In Brief

Bielmeier et al. demonstrate that actomyosin contractility increases at interfaces between normal and aberrantly specified epithelial cells. This drives apoptotic elimination, cyst formation, or cell segregation. These results thus provide a novel perspective on morphogenetic mechanisms arising from cell-fate heterogeneities within tissues.

Bielmeier et al., 2016, Current Biology *26*, 563–574
March 7, 2016 ©2016 Elsevier Ltd All rights reserved
http://dx.doi.org/10.1016/j.cub.2015.12.063

CellPress

Current Biology
Article

CellPress

Interface Contractility between Differently Fated Cells Drives Cell Elimination and Cyst Formation

Christina Bielmeier,[1,4] Silvanus Alt,[2,3,4] Vanessa Weichselberger,[1] Marco La Fortezza,[1] Hartmann Harz,[1] Frank Jülicher,[2] Guillaume Salbreux,[2,3,*] and Anne-Kathrin Classen[1,*]
[1]Ludwig-Maximilians-University Munich, Faculty of Biology, Grosshadernerstrasse 2-4, 82152 Planegg-Martinsried, Germany
[2]Max Planck Institute for the Physics of Complex Systems, Nöthnitzer Strasse 38, 01187 Dresden, Germany
[3]The Francis Crick Institute, Lincoln's Inn Fields Laboratories, 44 Lincoln's Inn Fields, London WC2A 3LY, UK
[4]Co-first author
*Correspondence: guillaume.salbreux@crick.ac.uk (G.S.), classen@bio.lmu.de (A.-K.C.)
http://dx.doi.org/10.1016/j.cub.2015.12.063

SUMMARY

Although cellular tumor-suppression mechanisms are widely studied, little is known about mechanisms that act at the level of tissues to suppress the occurrence of aberrant cells in epithelia. We find that ectopic expression of transcription factors that specify cell fates causes abnormal epithelial cysts in *Drosophila* imaginal discs. Cysts do not form cell autonomously but result from the juxtaposition of two cell populations with divergent fates. Juxtaposition of wild-type and aberrantly specified cells induces enrichment of actomyosin at their entire shared interface, both at adherens junctions as well as along basolateral interfaces. Experimental validation of 3D vertex model simulations demonstrates that enhanced interface contractility is sufficient to explain many morphogenetic behaviors, which depend on cell cluster size. These range from cyst formation by intermediate-sized clusters to segregation of large cell populations by formation of smooth boundaries or apical constriction in small groups of cells. In addition, we find that single cells experiencing lateral interface contractility are eliminated from tissues by apoptosis. Cysts, which disrupt epithelial continuity, form when elimination of single, aberrantly specified cells fails and cells proliferate to intermediate cell cluster sizes. Thus, increased interface contractility functions as error correction mechanism eliminating single aberrant cells from tissues, but failure leads to the formation of large, potentially disease-promoting cysts. Our results provide a novel perspective on morphogenetic mechanisms, which arise from cell-fate heterogeneities within tissues and maintain or disrupt epithelial homeostasis.

INTRODUCTION

Epithelial morphogenesis is tightly regulated to allow epithelia to act as barriers between exterior and interior environments and to fulfill functions such as protection, secretion, or absorption. Epithelial morphogenesis relies on the coordinated interplay between cell fate, cell shape, and tissue remodeling [1–4]. Epithelia thus need to eliminate aberrantly specified cells to prevent disruption of tissue function or the occurrence of cancer. However, while aberrantly specified cells have been observed in tumors, little is known about how aberrant fates contribute to disruption of epithelial integrity.

In development, cell fates are specified by signaling pathways, such as Wnt/β-catenin, transforming growth factor β (TGF-β)/SMAD, Shh/Ci, or JAK/STAT. Strikingly, cells with altered Wnt/β-catenin components give rise to abnormal epithelial cysts in mouse models of colon cancer [5, 6]. Altered expression of cell-surface molecules have been suggested to drive coordinated invagination of mutant cells into cysts. In *Drosophila* imaginal discs, cell clusters mutant for Wnt/β-catenin and TGF-β/SMAD components similarly disrupt epithelial continuity through formation of cysts [7–10]. In contrast to the surface-molecule-driven segregation of cell populations suggested to occur in the mouse colon, cell-autonomous reduction in mutant cell height has been implicated as direct cause of cysts in fly tissues [9, 10].

Cyst formation in *Drosophila* epithelia is not restricted to disruption of Wnt/β-catenin or TGF-β/SMAD signaling but was observed for various unrelated genetic alterations [11–25]. While cyst formation has severe consequences for epithelial function, it is not understood what cellular mechanisms drive cyst formation in these different contexts and if cyst formation is associated with a biological function. We thus sought to identify the cell-biological processes and physical forces driving cyst formation in *Drosophila* imaginal discs, which have been instrumental in elucidating mechanisms controlling epithelial architecture in development and disease. We wanted to specifically understand whether cell-autonomous shape changes [9, 10], expression of cell-surface molecules [5], coordinated apical constriction [26], or proliferation within a confined space [27] drive cyst formation to elucidate how aberrant cells disrupt epithelial integrity.

RESULTS

Misexpression of Cell-Fate-Specifying Transcription Factors Underlies Cyst Formation in Imaginal Discs

Imaginal discs mutant for the redundantly acting, homologous tumor suppressor genes *Posterior sex combs* (*Psc*) and *Suppressor of zeste 2* (*Su(z)2*) contain epithelial cysts [21]. *Psc* and

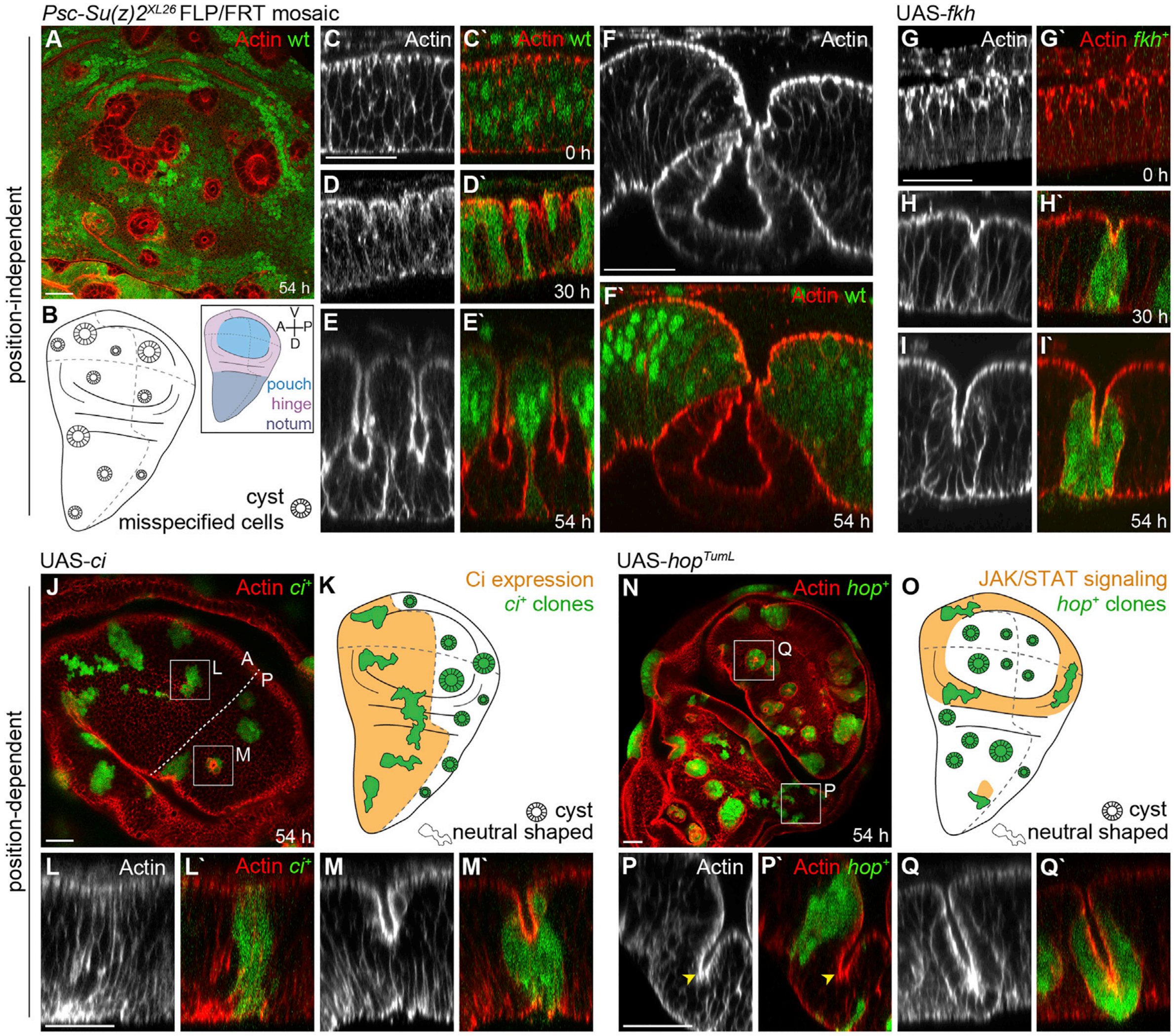

Figure 1. Ectopic Expression of Cell-Fate-Specifying Transcription Factors Causes Cysts

(A, C–J, L–N, P–Q) Wing disc pouch containing GFP-negative, *Psc/Su(z)2*^{XL26} clones (A–F; GFP is shown as green in A and C–F), or GFP-positive, *fkh*-expressing (G–I), *ci*-expressing (J–M), and *hop*^{tumL}-expressing (N–Q) clones (green in G–Q). Actin is shown in red or gray (A, C–I, J, L–N, P, and Q). Confocal xy sections at 54 hr (A, J, and N) and cross-sections (C–I, L, M, P, and Q) at 0, 30, and 54 hr after clone induction are shown.

(B) Scheme of position-independent cyst formation by *Psc/Su(z)2*^{XL26} and *fkh*$^{+}$ clones (gray). Inset defines disc subregions, compartment boundaries (dotted lines) and anterior, posterior, dorsal, and ventral axis.

(K and O) Scheme of position-dependent cyst formation by *ci*-expressing (K) and *hop*^{tumL}-expressing (O) clones in regions where Hh/Ci (K) or JAK/STAT (O) signaling (orange) is low.

Boxes in (J) and (N) frame clones whose cross-sections are displayed below. Arrowhead (P) points to endogenous tissue fold. Scale bars, 25 μm. See also Figures S1 and S2.

Su(z)2 encode Polycomb proteins, which epigenetically silence cell-fate-specifying transcription factors during development [28] and restrain proliferation by repressing JAK/STAT and Notch signaling [21, 29]. FLP/FRT-induced cell clusters ("clones") [30] homozygous for a precise deletion of both *Psc* and *Su(z)2* retracted from the apical surface of wing imaginal discs (Figures 1A–1D) and formed cyst-like structures locating to the basal side of the epithelium (Figures 1E and 1F). At late stages, many *Psc/Su(z)2* clones completely resolved contacts with wild-type cells and gave rise to persistent, proliferating cysts encapsulating an apical lumen (Movie S1).

To test whether high rates of cell proliferation were responsible for cyst initiation, we reduced proliferation in *Psc/Su(z)2* cells by interfering with the growth-promoting function of the Hippo/Yorkie pathway. We created *Psc/Su(z)2*, *yorkie* double-mutant clones and found that cysts still formed (Figures S1A–S1F). These observations strongly imply that cysts are not a result of spatial constraints imposed on proliferating *Psc/Su(z)2* cells.

In addition to restraining growth, Polycomb activity represses expression of numerous transcription factors involved in cell-fate specification [28]. To test whether fate misspecification in *Psc/Su(z)2* clones underlied cyst formation, we individually

 ©2016 Elsevier Ltd All rights reserved

overexpressed unrelated transcription factors silenced by *Psc/Su(z)2* (Figure S1M) using the GAL4/UAS flip-out system [30]. Intriguingly, ectopic expression of the forkhead-box transcription factor *fork head* (*fkh*) involved in salivary gland morphogenesis (Figures 1G–1I and S2A–S2D) [31], the homeobox factor *Abdominal-B* (*AbdB*) involved in segment specification (Figure S2F) [32], or the Runt-domain factor *lozenge* (*lz*) required for hemocyte differentiation (see Figure S2F) [33] were each sufficient to give rise to cysts. This suggests that ectopic activation of Polycomb-silenced cell-fate-specifying transcription factors may be sufficient to drive cyst formation in *Psc/Su(z)2* clones. Accordingly, downregulation of just one derepressed transcription factor, like *fkh* or *AbdB*, in Polycomb mutant cells is insufficient to prevent cysts [12] (data not shown).

We wanted to test whether cysts are specific to transcription factors silenced by *Psc/Su(z)2*, or to cell-fate misspecification in general. We ectopically expressed randomly selected transcription factors not regulated by Psc/Su(z)2 (Figure S1M). Clones expressing the homeobox factor *ultrabithorax* (*Ubx*) (see Figure S2F) or the Pax6-homolog *eyeless* (*ey*) (Figure S2J) caused cysts in wing discs. Ectopic expression of cell-fate transcription factors caused cysts in eye discs as well (Figures S2E and S2H). In contrast, a transcription factor involved in cellular growth (*dMyc*) [34] (Figures S2N and S2N′), transcription factors dependent on co-factors for activity (*pan*, *exd*) [14, 35] (data not shown), or a cytoplasmic protein characteristic of muscle fate (Figure S2N″), did not cause cysts. Combined, these observations suggest that aberrant activity of transcription factors specifying cell fate underlies cyst formation in imaginal discs.

Because we observed significant levels of apoptosis in clones overexpressing *fkh* (Figure S1I) or *ey* (Figure S6O), we tested whether inhibiting apoptosis could prevent cysts. However, neither co-expression of *p35* nor *dIAP1* prevented cysts (Figures S1J–S1L). Similarly, activation of JNK signaling could not account for cysts, as *Psc/Su(z)2* cysts still formed in discs mutant for the JNK-kinase *hemipterous* (*hep*) (Figures S1G and S1H). These results support that cell-fate misspecification, rather than a secondary stress response, underlies cyst formation.

The transcription factors tested caused cysts independent of clone position within the tissue (Figure 1B). Importantly, none of them are endogenously expressed in wings. In contrast, Wnt/β-catenin and TGF-β/SMAD mutant clones are reported to give rise to cysts dependent on position within endogenous signaling gradients (Figures S2P and S2Q) [7–10, 25]. While Wnt/β-catenin and TGF-β/SMAD-dependent cell-autonomous shape changes were suggested to cause cysts, we wanted to test whether, instead, cell-fate misspecification in general drives cyst formation. We thus generated clones activating downstream transcriptional effectors of the conserved patterning pathways Hh/Ci (Figures 1J–1M) or JAK/STAT (Figures 1N–1Q). We found that *cubitus interruptus* (*ci*)-expressing clones maintained normal epithelial shapes in anterior compartments, where Hh/Ci signaling is high (Figures 1J–1L). However, *ci*-expressing clones formed cysts in posterior compartments, where repression of *ci* prevents Hh/Ci signaling (Figures 1J, 1K, and 1M) [36]. Likewise, expression of a dominant-active JAK (*hop*$^{tum\text{-}L}$), which activates STAT [37], induced cysts only in pouch and notum regions of the disc, where JAK/STAT signaling is low (Figures 1N–1Q).

We found that misspecification within other patterning domains caused position-dependent cyst formation. Whereas *vestigial* (*vg*)-expressing clones gave rise to cysts in the hinge and notum, *homothorax* (*hth*)-expressing clones did so in the pouch. These patterns are complementary to endogenous regions of expression, which specify the proximal-distal axis of wing fates (Figures S2R and S2S). Ectopic expression of other factors, such as *Iro-C*, *salm*, and *omb*, were previously described to cause cysts in regions of low endogenous Iro-C, Spalt, and Omb activity [23–25]. Moreover, expression of the eye selector gene *eyeless* (*ey*) caused cysts in wings (Figure S2J) but rarely in eyes (Figure S2L). Collectively, these observations emphasize that cyst formation in imaginal discs represents a surprisingly general response to cell-fate misspecification and is driven by relative fate differences between misspecified and surrounding wild-type cells.

Cyst Formation Is Driven by Non-autonomous Enrichment of Actomyosin at the Interface between Misspecified and Wild-Type Cells

We then asked why cysts appear in response to the presence of differently fated cells. We first wanted to understand whether cyst formation is a cell-autonomous process, reflecting altered cell shape caused by altered gene expression [9, 10]. To visualize cell-autonomous changes, we generated wing discs where the majority of cells expressed *fkh* by lengthening the heat shock, thus increasing the likelihood of neighboring cells to activate the GAL4/UAS flip-out system [30]. While *fkh*-expressing cells remained columnar, we were surprised to find that small clusters of wild-type cells in the tissue retracted from the apical surface and gave rise to cysts (Figures 2A and 2C and S2A′–S2E′). Cyst formation of wild-type clones could also be induced by overexpression of *AbdB*, *Ubx*, or *ey* (Figures 2B, S2G, S2I, S2K, and S2M). Similarly, broad expression of *ci* caused wild-type cysts, however, only in posterior compartments where Hh/Ci signaling is low (Figure S2U). Our results therefore indicate that cyst formation is not driven cell autonomously by misspecified cells, but instead by apposition of differently fated cells. Consequently, we reasoned that cysts must form by a mechanism acting at the misspecified wild-type cell interface (MWI).

To elucidate this mechanism, we analyzed cell adhesion, polarity, and cytoskeletal markers in early cysts. However, levels and localization at interfaces of *fkh*-expressing or *Psc/Su(z)2* cells were not consistently different to those at interfaces of wild-type cells (data not shown). Actin enrichment at apical surfaces of invaginating cells is likely a consequence of apical constriction, as it was seen in misspecified and wild-type cysts (compare Figure 1H with Figures 2A and 2B).

Next, we focused on interfaces between different cell fates. We found that phalloidin-labeling intensities of actin at MWI adherens junctions of *fkh*-expressing clones were increased by 30%, even if some clones had not yet undergone invagination (Figures 2D, 2E, S3N, S3P, and S3R). Importantly, we found that, in addition, actin-labeling intensities were 40% higher at basolateral MWI interfaces (Figures 2D, 2F, S3O, S3Q, and S3R). At late stages of cyst formation, enrichment at the MWI persisted (Figures 2E, 2F, and S3R). Increased actin was also detected at the MWI of *ey*-expressing clones (Figure S3S) and when wild-type cells formed cysts (Figures 2E, 2F, S2B′, S2D′, S2E′,

CellPress

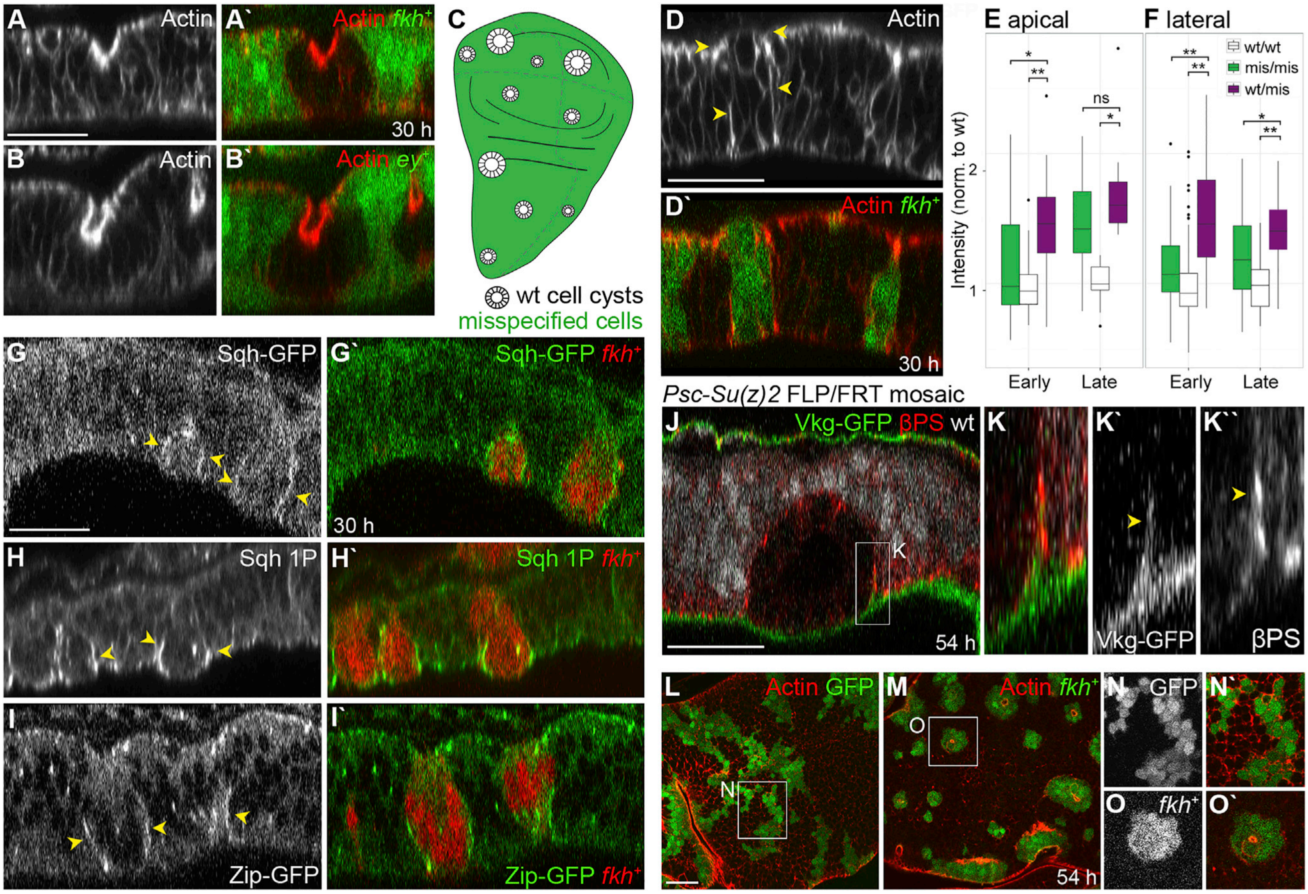

Figure 2. Cyst Formation Is Cell Non-autonomous and Correlates with Actomyosin Enrichment at the MWI

(A and B) xz cross-sections of wild-type clones 30 hr after induction of large domains of (A) *fkh*$^+$- or (B) *ey*$^+$-expressing cells (green). Actin in red or gray.

(C) Scheme of position-independent wild-type cysts surrounded by misspecified cells (green).

(D and G–I) xz cross-section of *fkh*-expressing clones (green, D; red, G–I) 30 hr after induction in discs stained for Actin (D), expressing Sqh-GFP (G), stained for Sqh-1P (H), or expressing Zip-GFP (I) (gray or red, D; green, G–I). Arrowheads highlight enrichment at MWI.

(E and F) Boxplots of normalized actin intensity at apical adherens junction (E) and basolateral interfaces (F) between wild-type (wt/wt), misspecified *fkh*$^+$ (mis/mis), and wild-type and *fkh*$^+$ cells (wt/mis). A two-tailed WSR test was applied. *p < 0.01, **p < 0.001; ns, not significant. See Figure S3R for details.

(J and K) xz cross-section of *Psc/Su(z)2*^{XL26} clones in discs expressing CollagenIV-GFP (Vkg-GFP), stained for βPS-Integrin (βPS) 54 hr after induction. Wild-type cells are gray in (J). Arrowheads (K) point to basement membrane deformation at MWI.

(L–O) xy sections of wild-type (green or gray, L and N) and *fkh*-expressing clones (green or gray, M and O) 54 hr after induction at one-third of cell height. Boxes frame regions shown at higher magnification in (K), (N), and (O). Scale bars, 25 μm. See also Figures S2 and S3.

and S3R). The resolution of a confocal microscope did not allow us to distinguish in cell-autonomous actin-labeling experiments if actin enriched at just one or both interface cortices. However, actin enrichment at the MWI, rather than cell-autonomous changes in misspecified or wild-type cells, appears to be a defining feature of early and late cyst stages.

A detailed analysis of cytoskeletal components demonstrated that the myosin II regulatory light-chain Sqh (Figures 2G, S3A, and S3B), an activated form of Sqh (Figures 2H, S3C, and S3D), the heavy-chain Zip (Figures 2I, S3E, and S3F), the FERM-domain protein Moesin (Figures S3I and S3J), and an activated form of Moesin (Figures S3K–S3M) localized to basolateral MWIs. In contrast, the myosin II regulator Rho1 was not enriched at MWI interfaces (Figures S3G and S3H). These observations demonstrate that, like actin, activated myosin and moesin are specifically recruited to the MWI and suggest that the MWI may be under increased actomyosin-mediated contractile tension.

Concurrent with enrichment in contractile components, we observed dramatic changes to the shape of the MWI. The basement membrane underwent an upward deformation accompanied by focused integrin (Figures 2J and 2K) and actin (Figures S3O and S3Q) enrichment at the MWI. These changes may reflect actin polymerization and integrin engagement as cells respond to contraction of the MWI away from the basement membrane.

While smoothening of adherens junction between differently fated cells has been described (i.e., [38]) (Figures S3E, S3N, and S3P), we observed that misspecified clones also exhibited striking minimization of basolateral contact areas (Figures 2L–2O). Basolateral clone circularity increased from 0.32 in wild-type to 0.76 in *fkh*-expressing clones (Figures S3T and S3U)

 ©2016 Elsevier Ltd All rights reserved

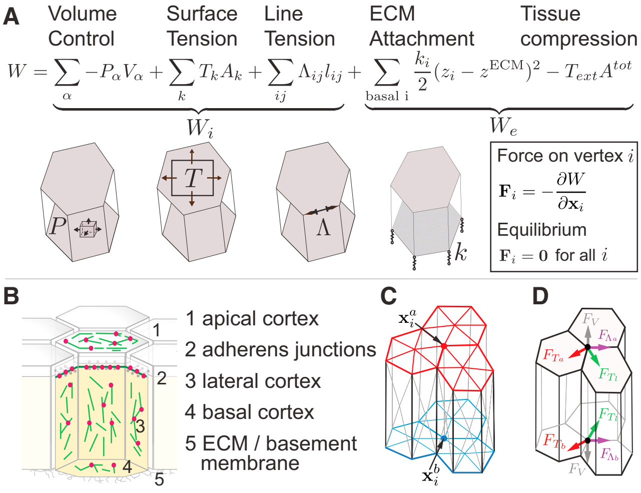

Figure 3. A Physical Description of Epithelia in a Three-Dimensional Vertex Model

(A) Forces are obtained from an effective mechanical work function W that is the sum of internal and external work functions, W_i and W_e. W_e takes into account (1) intracellular pressure P_α constraining cell volume, (2) surface tensions T_k acting on cell surfaces k, and (3) line tensions Λ_{ij} acting on edges between vertices i and j. W_e takes into account (1) springs resisting the deformation of basal vertices away from a reference plane and (2) external forces establishing compressive stress $T_{ext} < 0$.

(B) Epithelial surface tensions arise from actomyosin cortices (actin green, myosin red) associated with apical, lateral, and basal faces. Line tensions arise from actin cables observed at adherens junctions. Extracellular matrix proteins (ECMs) cover the basal tissue surface.

(C) In the model, tissue geometry is characterized by a set of vertices with positions x_i. An additional vertex is introduced at the barycenter of each surface. Triangles connecting central and contour vertices define cell boundaries.

(D) Forces acting on vertex i are obtained by differentiating the mechanical work with respect to vertex positions x_i. Forces have contributions from surface tensions (F_T), line tensions (F_Λ), and cellular pressures (F_V).

See also Figure S4.

and was similar to circularity of cysts formed by wild-type cells (Figure S3T).

In summary, recruitment of Actin, Myosin, and Moesin to the MWI correlated with dramatic minimization of the entire lateral contact area between wild-type and misspecified cells. This causes clones to acquire a characteristic smooth ball-like shape and culminates in complete resolution of MWI contacts, releasing *Psc/Su(z)2* cysts from the epithelium (Movie S1). We thus suggest that contractility at the MWI is indeed higher than at other cellular interfaces in the tissue.

Changes to Mechanical Properties of the MWI Are Sufficient and Necessary to Recapitulate Cyst Formation

To understand how mechanical properties of cells and changes in the distribution of cytoskeletal forces could drive cyst formation, we developed a three-dimensional vertex model for epithelia (Figures 3 and S4A–S4C; see "Modeling Procedures" in the Supplemental Information). We simulated the presence of a clone by placing a number of misspecified cells N_c within a wild-type cell population (Figure 4A) and then applied two types of mechanical changes. We modified line or surface tensions (1) in misspecified cells ("bulk contractility"; Figures 4B and 4C) or (2) only at the interface between misspecified and wild-type cells ("interface contractility"; Figures 4D and 4E).

Because reduction in cell height has been previously linked to cyst formation [7–10], we first performed bulk contractility simulations where all misspecified cells experienced increased lateral surface tensions. This perturbation altered preferred aspect ratios toward cuboidal shapes and indeed caused cysts in simulations (Figure 4B). However, cyst formation by wild-type cells could not be recapitulated: wild-type cells did not invaginate but remained tall (Figure 4C).

To confirm this prediction experimentally, we expressed an activated form of the Rho1-GTPase, which caused actin to accumulate at lateral cortices and reduced the cell height (Figure 4H) [10]. As predicted by simulations, small *Rho1^{V14}*-expressing clones caused deep indentations in discs (Figures 4F–4H). However, overexpression of *Rho1^{V14}* in large domains did not cause the remaining wild-type cells to invaginate (Figures 4I and 4J). Instead, both cell types exhibited different heights and, as predicted by simulations (Figures 4B and 4C), failed to minimize MWI contacts (Figures 4I, 4J, and S3T). Our results thus confirm that altering mechanical properties of individual cells can cause cysts but cannot induce cysts by wild-type cells. This suggests that cysts observed after cell-fate misspecification do not solely arise from cell-autonomous shape changes. Instead, apposition of fates induces a cellular response upstream of shape changes within misspecified cells, which drives cyst formation.

We therefore performed simulations to test whether MWI "interface contractility" is sufficient for cyst formation by misspecified and wild-type cells. We increased apical line tension and lateral surface tension at the interface and found that clones deformed into cysts (Figure 4D). As these simulations only involved changes to the MWI, inverse cysts by wild-type clones were recapitulated as well (Figure 4E). We therefore conclude that contractility at the MWI is higher than in the rest of the tissue and is sufficient and necessary to account for all cyst configurations observed in our experiments.

Cyst Formation Is Restricted to an Intermediate Range of Clone Sizes

We noticed that interface simulations predicted a strong dependency of final clone shape on clone size. To better analyze this dependency, we turned to a continuum theory of tissue mechanics, which includes only a few key parameters allowing us to draw generic conclusions on tissue shape stability. On large spatial scales, the vertex model epithelium effectively behaves as a continuous elastic sheet (Figure S4H; see "Modeling Procedures" in the Supplemental Information). Elastic sheets buckle if compressed, potentially driving cyst formation in our vertex model simulations. The threshold of buckling is determined by two considerations: First, for a circular contractile boundary, the compression felt by the enclosed elastic sheet depends on the inverse radius of the boundary, as described by the law of Laplace (Figure 5A). Therefore, large clones feel less pressure from the boundary and are less likely to buckle. Second, the resistance of an elastic sheet to bending is higher on small length scales (Figure 5B). Small clones therefore have a higher resistance to buckling than larger clones. The combination of these two effects implies that very small and very large clones do not

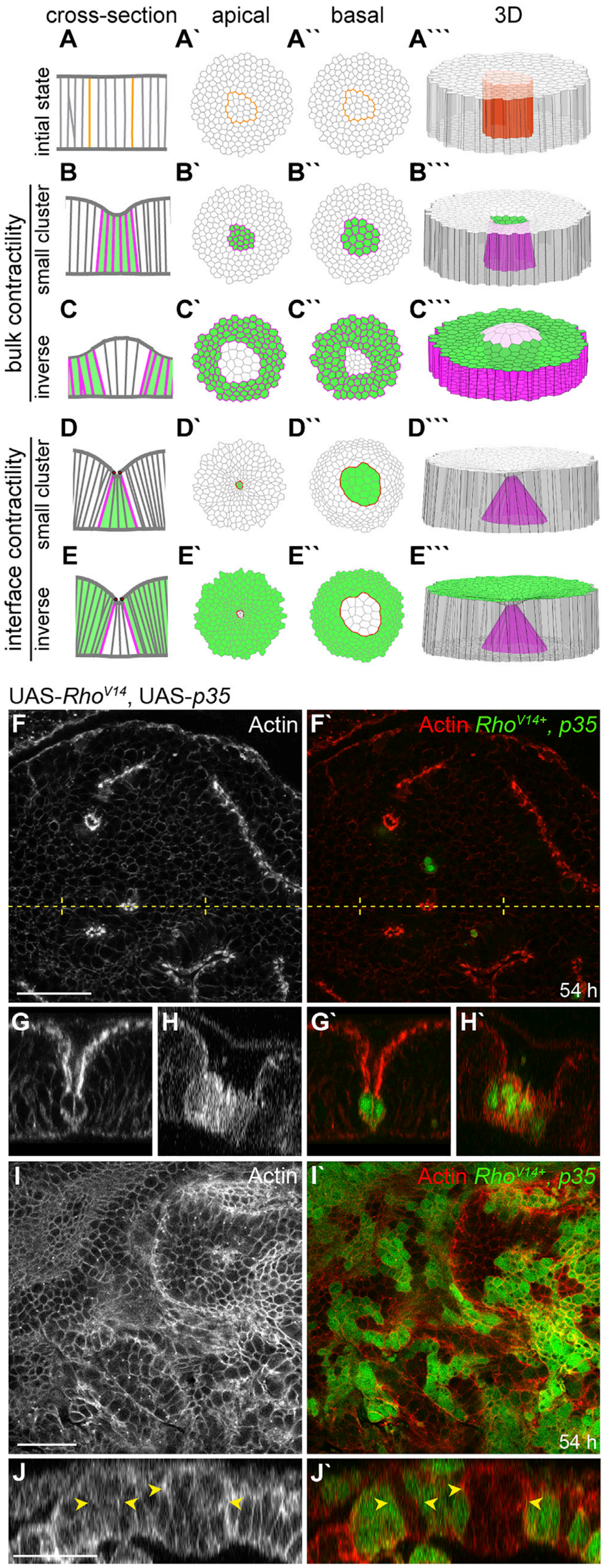

Figure 4. MWI Contractility Is Sufficient and Necessary to Recapitulate Cyst Formation

(A–E′′′) Vertex model simulations visualize epithelial shapes in cross-section (A–E), apical (A′–E′), basal (A′′–E′′), and 3D (A′′′–E′′′) views. A clone of 20 misspecified cells is shown before (A) and after changes to mechanical properties of misspecified cells (green) (B and C, "bulk contractility") or the MWI (D and E, "interface contractility"). Magenta and red lines represent a 3-fold increase in lateral surface and apical line tension, respectively.

(F–J) xy sections (F and I) and cross-sections (G, H, and J) of Rho^{V14},*p35*-expressing cells (green) 54 hr after induction. Actin is in gray or red. Arrowheads in (J) point to interspersed wild-type clones failing to form cysts. Dotted line in (F) indicates position at which cross-section (G) was reconstructed. Scale bars, 25 μm.

See also Figure S3.

form cysts. Indeed, we find that a circular elastic sheet under tension, connected to an external extracellular matrix (ECM) and subjected to a contractile circular boundary only buckles for intermediate size ranges (Figures S4K and S4L).

To confirm this prediction experimentally, we carried out a quantitative analysis of *fkh*-expressing clone shapes as a function of clone size. We measured apical and basal clone width w_a and w_b, as well as apical and basal deformation, u_a and u_b (Figure 5E). This analysis revealed that apical and basal deformations are maximal for intermediate clone sizes (N_c ~70 cells), and minimal for either small or very large clones (Figures 5C and 5F′–5J′). Similarly, differences in apical and basal widths were maximal for intermediate clone sizes, corresponding to strongly wedge-shaped cysts (Figures 5D and 5F′–5J′). Very small and very large clones do not undergo strong deformations but still experience a decrease in MWI roughness (Figures 5F′, 5J′, and S3O). These quantitative results align with the predicted size-dependent impact of a contractile interface on final clone shape in simulations and further support the notion that a contractile MWI underlies cyst formation.

A 3-Fold Simulated Increase in MWI-Tension Recapitulates Shape Parameters of Experimental Cysts

Having established experimentally for which clone sizes maximal deformations occur, we performed 3D vertex model simulations (Figures 5F–5J) to determine what range of physical forces could explain the observed relationship between clone shapes and clone size. We first established a set of mechanical parameters that reproduced experimentally measured cell aspect ratios in wild-type discs (Figure S4F; see "Modeling Procedures" in the Supplemental Information), including a parameter for ECM-induced compression [39, 40] set by measured aspect ratio changes after collagenase treatment (Figures S4D–S4G).

We then simulated cysts and measured shape parameters as for experimental clones (Figures 5C–5E). To match experimental measurements, we adjusted (1) apical, lateral, and basal surface tension of the tissue, (2) apical and basal line tension, (3) overall tissue compression, (4) stiffness of tissue-ECM attachment, and (5) apical line and lateral surface tensions at clone boundaries (Figure S5; see "Modeling Procedures" in the Supplemental Information). In doing so, we searched for the smallest increase in MWI contractility that could account for experimental measurements. We found that a 3-fold increase in apical line and lateral surface tensions at the MWI can recapitulate all features of the four experimental curves for w_a, w_b, u_a, and u_b (Figures

 ©2016 Elsevier Ltd All rights reserved

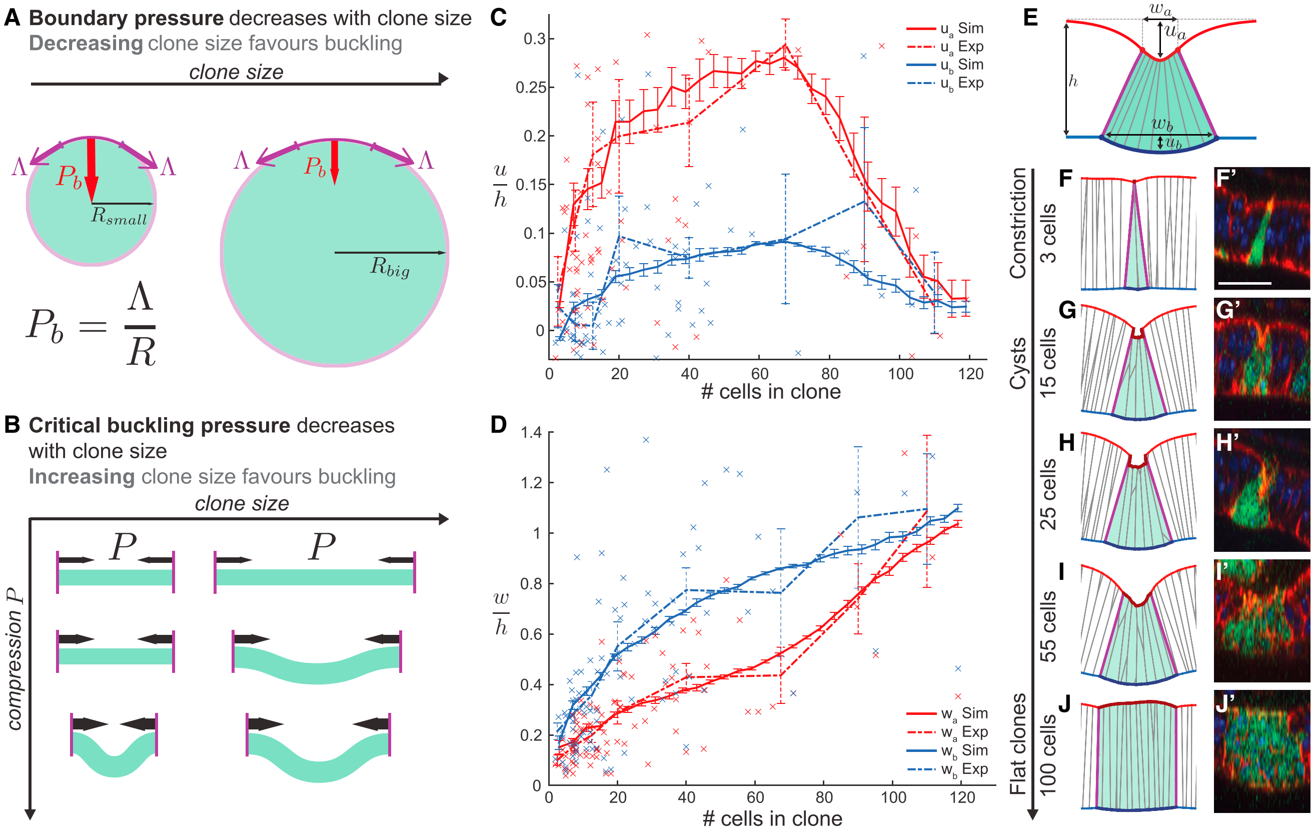

Figure 5. Final Clone Shape Depends on Clone Size

(A) Laplace's Law ($P_b = \Lambda/R$) predicts that the pressure P_b exerted by a contractile boundary with line tension Λ depends on the radius R of the enclosed material. Thus, large clones feel less pressure from a contractile boundary and are less likely to buckle.

(B) The resistance to bending of an elastic disk depends on its radius. Smaller clones exhibit higher resistance to buckling than larger clones.

(C and D) Experimental (dotted) and simulated (continuous line) deformations of apical (red) or basal (blue) cyst surfaces with respect to clone size. Parameters u_a, u_b (C) and w_a and w_b (D) are illustrated in (E). Error bars represent mean and SEM of 85 *fkh*-expressing clones 30 hr after induction and 15 simulations per data point.

(E) Deformation parameters measured experimentally and fitted by simulations. w_a, apical clone width; w_b, basal clone width; u_a, apical surface indentation; u_b, basal surface deformation.

(F–J) Simulated and experimental cross-sections of clones containing different cell numbers. Apical constriction, cyst formation, or minimal deformations correlate with clone size. Note that cross-section choice results in junctions not spanning apico-basal axis. Scale bars, 25 μm.

See also Figures S4 and S5.

5C and 5D). Importantly, an increase in apical line or lateral surface tensions alone did not recapitulate experimental observations (Figures S5E–S5G), emphasizing the functional significance of actomyosin enrichment at both adherens junctions and basolateral interfaces (Figures 2E and 2F). Furthermore, bulk contractility simulations resulted in clone shapes that did not correspond to those observed in experiments (Figure S5B), reinforcing our conclusion that bulk contractility cannot account for all phenotypes.

We then tested whether a buckling instability can indeed account for tissue deformations predicted by the 3D vertex model. We calculated estimates of the coarse-grained elastic modulus, surface tension, and bending modulus of a tissue represented by the vertex model, with line and surface tensions obtained by the fitting procedure described above (Figures S4H–S4J; see "Modeling Procedures" in the Supplemental Information). Using these parameters, we found that a buckling instability is predicted for clones below ~106 cells (Figures S4K and S4L), in qualitative agreement with the size range of experimental and vertex model cysts (Figures 5C–5J). Therefore, a buckling transition captures the maximum clone size below which cyst formation occurs.

Single Misspecified Cells Are Eliminated from Epithelia by MWI Contractility

In simulations and experiments, very small clones do not form cysts but display significantly reduced apical areas (Figure 5F). This resembles initial stages of cell extrusion events occurring during clearing of apoptotic or live cells [41]. It suggested that MWI contractility may specifically drive elimination of small misspecified cell clusters by promoting apical constriction and extrusion.

To understand whether size-dependent elimination of misspecified clusters indeed occurred, we quantified the frequencies of

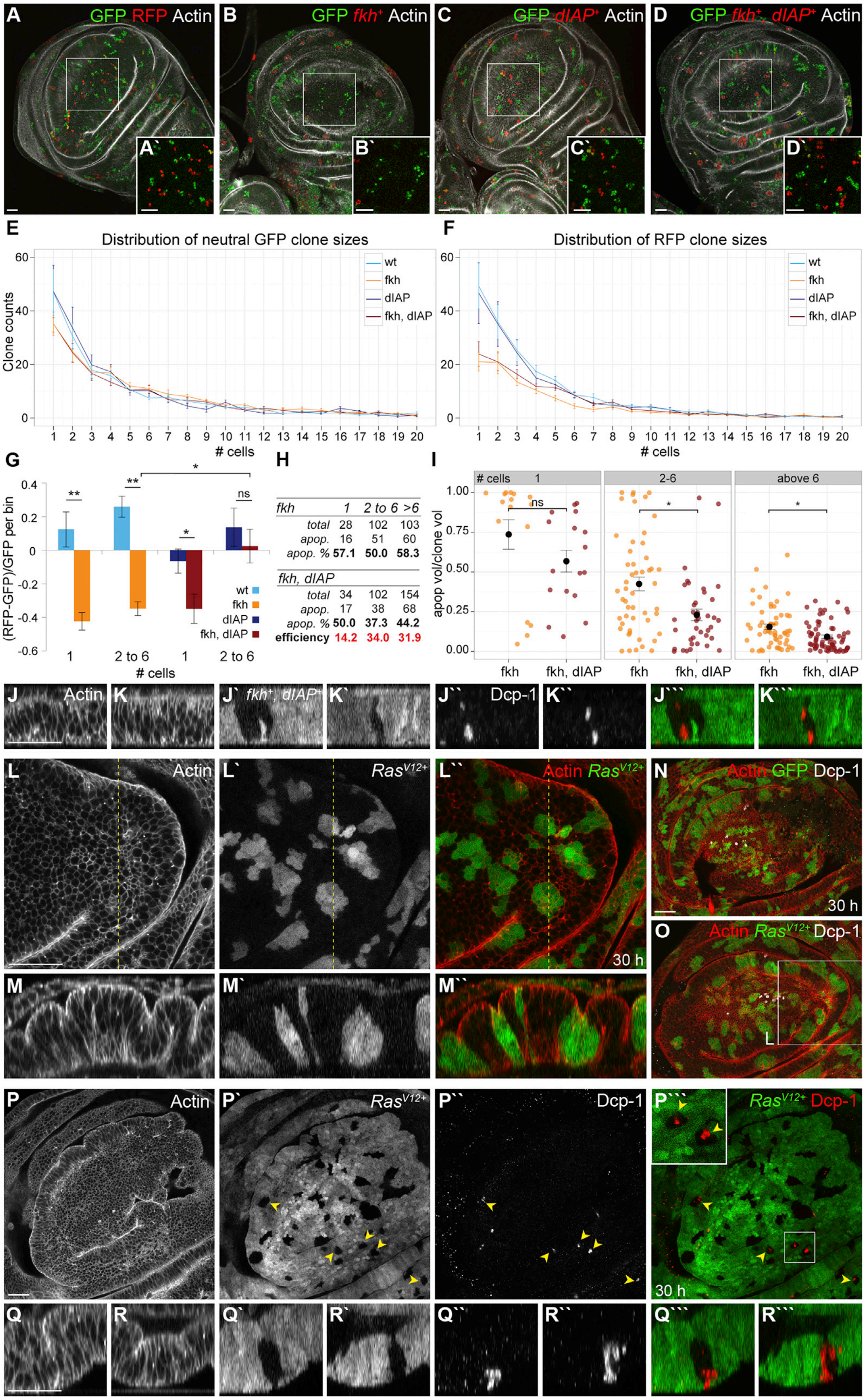

(legend on next page)

 ©2016 Elsevier Ltd All rights reserved

misspecified clone sizes and compared them to those of wild-type clones. To control for experimental variability, we used the Tie-Dye technique [18] to generate misspecified cells marked by RFP and wild-type cells marked by GFP in the same disc (Figures 6B–6D). To normalize for differences in frequencies of GFP and RFP clone induction, we compared clone counts to those of neutral control discs (Figure 6A). When we analyzed Tie-Dye discs, we found that *fkh*-expressing clones of up to six cells were indeed dramatically underrepresented (Figures 6E–6G and S6A–S6D). Similarly, we observed dramatic loss of *ci*-expressing clones in the posterior compartment of Tie-Dye discs (Figures S6F–S6I).

We wanted to understand how these cells are eliminated. Because misspecified cells often underwent apoptosis (Figures S1I and S6O), we asked whether small *fkh*-expressing clones exhibited higher levels of apoptosis than larger clones. We quantified the volume occupied by apoptotic cells positive for cleaved Caspase Dcp-1 in *fkh*-expressing clones (Figure S6K). While the relative number of apoptotic clones did not change between small and large clone size bins (Figure 6H), we found that the number of apoptotic cells was significantly increased in clones containing up to six cells compared to clones larger than six cells (Figure 6I). This suggests that small *fkh*-expressing clones may be subject to stronger apoptotic stimuli than larger clones.

To understand whether apoptosis is necessary for elimination of small clones, we inhibited apoptosis by co-expressing *dIAP1* and analyzed *fkh,dIAP1*-expressing clone sizes (Figures 6E–6G and S6A–S6D) and Dcp-1 volumes (Figures 6H, 6I, S6E, S6J, and S6L). Expression of *dIAP1* rescued large *fkh*-expressing clone sizes back to wild-type sizes indicating that apoptosis in large clones is strongly reduced (Figures 6G and S6A–S6D). In contrast, *dIAP1* expression was not able to prevent loss of single *fkh*-expressing cells (Figures 6G and S6A–S6D). In addition, *dIAP1* expression reduced the number of large apoptotic *fkh*-expressing clones more efficiently than of single-cell clones (Figure 6H). Similarly, while dIAP1 reduced the number of apoptotic cells in large *fkh*-expressing clones, it did not alter relative apoptotic volumes in single cells (Figures 6I and S6E). Combined, these experiments suggest that specifically small *fkh*-expressing clones experience strong apoptotic stimuli, which cannot be counteracted by co-expression of rate-limiting levels of dIAP1, and ultimately drive elimination of small clones.

We hypothesized that if apoptosis in small clones is specific to MWI contractility, then apoptosis must also be induced in small wild-type clones encircled by MWI contractility. We thus examined wild-type clones after induction of large *fkh* or *ey*-expressing domains. We observed indeed frequent Dcp-1 activation in small wild-type cysts (Figures 6J, 6K, S6M, S6N, and S6P). Combined, our results strongly suggest that MWI contractility may drive cell elimination by inducing apoptosis specifically in small MWI-encircled cell clusters.

We next investigated a potential relevance of our observations to disruption of epithelial integrity in cancer. Specifically, we wanted to understand whether the occurrence of round clones in discs upon overexpression of oncogenic *Ras* (Ras^{V12}) [16] is driven by MWI contractility. Indeed, when we visualized small Ras^{V12}-expressing clones, we found that they formed cysts (Figures 6L, 6M, and 6O) [22]. Excitingly, we found that wild-type clones surrounded by Ras^{V12}-expressing cells undergo interface smoothening and cyst formation (Figures 6P–6R). This suggests that oncogenic Ras induces MWI contractility, likely because Ras also specifies cell fate. While we rarely observed apoptosis in wild-type or Ras^{V12}-transformed cells (Figures 6N and 6O), we found that apoptosis is frequently activated in small wild-type clones surrounded by Ras^{V12}-expressing cells (Figures 6P–6R) where Ras^{V12}-induced MWI-effects are strongest. Combined, these results reinforce our conclusion that MWI contractility is induced by apposition of cell populations with different fates and that MWI contractility drives cell elimination by activation of apoptosis in small, encircled cell clusters.

DISCUSSION

We describe here the biological and mechanical effects of a cellular mechanism acting between differently fated epithelial cells (Figure 7). We find that actomyosin recruitment to an interface between different fates promotes extrusion of single cells (Figure 7A). In contrast, interface contractility around

Figure 6. Small Misspecified Clusters Are Eliminated from Epithelia by MWI Contractility

(A–D) Tie-Dye discs 30 hr after induction carrying neutral GFP-expressing (green) and RFP-expressing clones (A) or fkh^+- (B), $dIAP1^+$- (C), and $fkh,dIAP1^+$-expressing clones (D) (red; Actin in gray). Boxes frame position of higher magnification insets.

(E and F) Neutral GFP (E) and transgene-expressing RFP (F) clone size frequencies 30 hr after induction. RFP^+ clones express either RFP alone or fkh^+ and/or $dIAP1^+$, as indicated. Histograms display clone counts for each clone size, binned into single-cell steps.

(G) Relative loss of fkh^+ and $fkh,dIAP1^+$-expressing clones compared to wild-type or $dIAP1^+$ control clones. For each disc, GFP^+ clone counts were subtracted from RFP^+ clone counts per size bin and normalized to GFP^+ clone counts for the respective bin.

(E–G) Mean and SEM of n = 8–10 discs for each genotype, analyzed by one-tailed WMW or Welch's t tests are shown. *p < 0.01, **p < 0.001, ns, not significant. See Figures S6A and S6D for details.

(H) Counts of fkh^+- or $fkh,dIAP1^+$-expressing apoptotic clones binned into three size categories: one cell, two to six cells, and above six cells. Total counts, apoptotic counts, and percentages of apoptotic clones per size bin are shown. Efficiency of inhibiting apoptosis by *dIAP1* expression was calculated as percentage of apoptosis in fkh^+ (n = 3 discs, 233 clones) / percentage of apoptosis in $fkh,dIAP1^+$ clones (n = 3 discs, 290 clones) per bin size.

(I) Dot plot of Dcp-1-positive volume fractions in fkh^+- and $fkh,dIAP1^+$-expressing apoptotic clones binned into indicated size classes. Mean and SEM within bins analyzed by two-tailed WMW tests are shown. *p < 0.01; ns, not significant. See Figure S6E for details.

(J–K‴) Cross-sections of $fkh,dIAP1^+$-expressing cells (J′ and K′; green, J‴ and K‴) 30 hr after induction. Actin (J and K), Dcp-1 (J″ and K″; red, J‴ and K‴).

(L–M″) Ras^{V12}-expressing clones (L′ and M′; green, L″ and M″) 30 hr after induction, stained for Actin (L and M; red, L″ and M″). Line indicates position at which xz cross-section (M) was reconstructed.

(N and O) GFP- (N) or Ras^{V12}-expressing clones (O) (green) stained for Actin (red) and Dcp-1 (gray).

(P–R‴) xy sections (P) and xz cross-section (Q and R) of Ras^{V12}-expressing cells (P′–R′; green, P‴–R‴) 30 hr after induction stained for Actin (P–R) and Dcp-1 (P″–R″; red, P‴–R‴). Box frames higher magnification inset. Arrowheads point to apoptotic wild-type clones.

Scale bars, 25 μm. See also Figure S6.

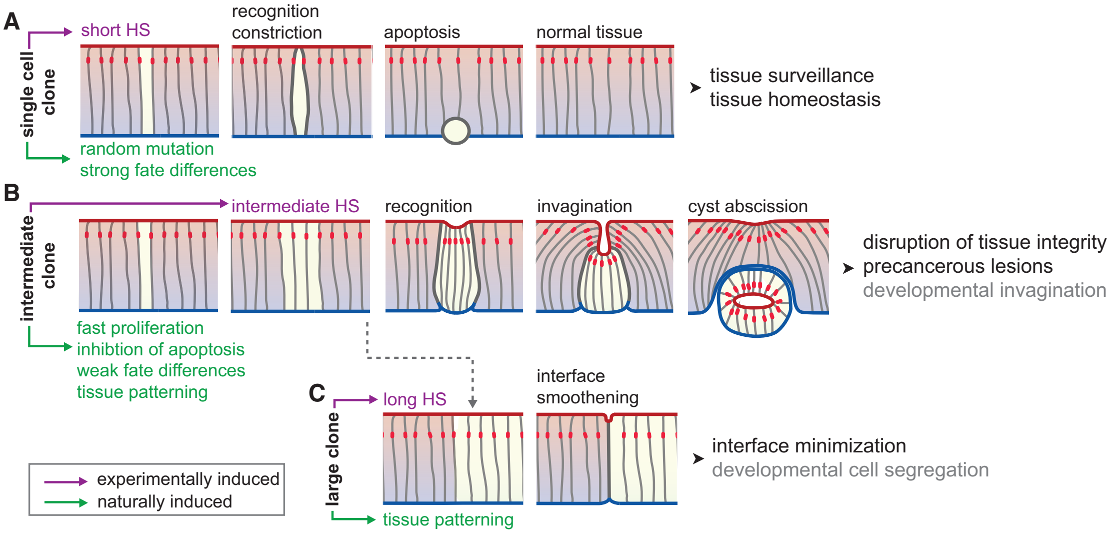

Figure 7. Morphogenetic Behaviors Induced by Interface Contractility

Tissue with apical (red), basal (blue), lateral surfaces (gray), and adherens junctions (red). Magenta indicates experimentally induced conditions (HS, heat shock), green potentially natural scenarios creating differently fated clone sizes. Gray indicates speculation on a role of interface contractility in development.

(A) Single misspecified cells are experimentally induced by a short heat shock (HS). Random mutations arise naturally in single cells and may cause fate differences. Interface contractility causes apical constriction and apoptosis to preserve tissue homeostasis.

(B) Intermediate-sized clones are induced experimentally by intermediate HS. Misspecified cell clusters may arise naturally from single cells that proliferate before detection or escape apoptosis by potent onco- or tumor-suppressor-gene mutations. During development, intermediate clusters arise by patterning. Cysts compromise tissue integrity and potentially promote precancerous lesions.

(C) Large clones are induced experimentally by long HS. During development, large lineage domains arise by patterning and tissue growth. Interface contractility leads to interface smoothening as observed at lineage boundaries.

intermediate-sized cell clusters induces apical buckling into cysts (Figure 7B). In large clones, interface contractility solely decreases interface roughness (Figure 7C). We thus suggest that interface contractility acts as surveillance mechanism on single misspecified cells but also drives disease-promoting disruption of epithelial integrity by deforming cell clusters into cysts. We speculate that interface contractility may have broader functions, such as establishing compartment boundaries between different cell populations during development.

Our data suggest that MWI contractility causes cell elimination by triggering apoptosis. The signal that activates apoptosis in small clones may arise from strong apical constriction. Apical constriction may limit apical area and thus receptors available for survival signals or may regulate mechano-sensitive survival pathways such as Hippo/Yorkie [42]. Another interface-dependent process called cell competition [43], which kills metabolically unfit "loser" cells that touch fitter "winner" cells, is unlikely to play a role. Cell competition fails to elicit contractile changes at winner-loser cells interfaces and, importantly, acts unidirectional by eliminating loser cells, even if encircled by winner cells. We suggest that epithelia likely evolved multiple mechanisms to eliminate homeostatic perturbations in either metabolic activity (cell competition) or cell fate (MWI contractility).

Paradoxically, cysts are caused by a failure to eliminate intermediate-sized cell clusters. In tissues, intermediate-sized clusters may arise by proliferation of aberrant apoptosis-resistant cells. Prominently, MWI-contractility-inducing tumor suppressor mutations, such as *Psc/Su(z)2*, or transformation with oncogenic Ras^{V12} may confer apoptosis resistance. Strikingly, in mouse models of colon cancer, cysts have been observed upon deregulation of Wnt/β-catenin or TGF-β/SMAD signaling [5, 6, 44]. Our work demonstrates that disruption of many patterning fields causes cysts, emphasizing that cysts may be an early hallmark of epithelial cancers driven by mutagenic changes to cell fate. Because cysts survive abscission from tissues, their formation may promote displacement of cells into new microenvironments and may precede emergence of invasive behaviors.

In our study, many transcription factors induced MWI contractility. Previous publications have described cysts in mosaic analysis of fate specification in imaginal discs and even neuroepithelia [11–25]. We suggest that cells likely detect cell-surface cues to compare fates. Given the diversity of fates we investigated, cells must likely use multiple proteins to reference identity and regulate contractility at MWIs. Ephrin signaling [45], LRR transmembrane proteins like Capricious and Tartan [46], or Toll-receptor patterning [47] may be potential mediators of fate recognition.

Representing the tissue as an elastic sheet in a continuum theory allowed us to identify the mechanical principles driving cyst formation based on two simple physical effects that control tissue buckling instability: the law of Laplace and the resistance of tissues to bending. These findings were confirmed by a novel vertex model of epithelia, which allowed us to simulate detailed three-dimensional cellular structures. Previous studies highlighted the importance of line tension for interface morphology [4]. However, cellular forces associated with interface mechanics in three dimensions have not been explored. We show that in addition to adherens junctions, contractility at basolateral interfaces is extensively regulated. Taking this third dimension of

 ©2016 Elsevier Ltd All rights reserved

cellular forces into account has crucial consequences for our understanding of 3D-tissue morphology, as it induces a repertoire of tissue deformation including cell extrusion, invagination, and interface smoothening. Our simulations suggest that a 3-fold increase in lateral surface tension and in apical line tension is required to account for these deformations. This increase is similar to the 2.5-fold increase in line tensions that has been estimated to act at the interface between developmentally specified compartments [48]. It will thus be crucial to investigate whether cellular mechanisms that regulate interface contractility at aberrantly specified cells and at developmental compartments are alike [4, 48]. Similarly, many developmental invagination processes are driven by cell-fate specification of intermediate-sized cell clusters [36, 49]. These processes therefore offer an opportunity to understand similarities and differences between morphogenetic behaviors driven by apposition of differently fated cells in development or disease.

EXPERIMENTAL PROCEDURES

Fly Genetics

For detailed genotypes and experimental conditions, please refer to Table S1 and Supplemental Experimental Procedures. Briefly, FLP/FRT and "GAL4/UAS flip-out" crosses were raised at 25°C and flipase expression was induced 72 hr after egg lay (AEL) by a heat shock at 37°C. Tissues were analyzed at indicated time points after heat shock.

Immunohistochemistry and Imaging Processing

Discs were dissected and fixed in 4% formaldehyde/PBS for 18 min. Washes were performed in PBS + 0.1 Triton X-100 (PBT) and blocking in PBT+5% normal goat serum. Discs were incubated with primary antibodies (Supplemental Experimental Procedures) overnight at 4°C. Secondary antibodies were incubated for 2 hr at room temperature. Samples were imaged using a Leica TCS-SP5 confocal microscope. Images were processed and analyzed using workflows established in Fiji (Supplemental Experimental Procedures).

SUPPLEMENTAL INFORMATION

Supplemental Information includes Supplemental Experimental Procedures, modeling procedures, six figures, one table, and one movie and can be found with this article online at http://dx.doi.org/10.1016/j.cub.2015.12.063.

AUTHOR CONTRIBUTIONS

C.B., S.A., G.S., and A.-K.C. designed the experiments; C.B., S.A., V.W., and H.H. performed the experiments; C.B., S.A., V.W., M.L.F., G.S., and A.-K.C. analyzed the data; S.A., F.J., and G.S. designed the 3D vertex model; and C.B., S.A., G.S., and A.-K.C. wrote the manuscript.

ACKNOWLEDGMENTS

We thank M. Juenger, I. Hariharan, D. Bilder, F. Schnorrer, R. Ward, and N. Azpiazu for sharing reagents. We thank BDSC for providing fly stocks, and DSHB for antibodies. We thank H. Leonhardt and Nanosystems Initiative Munich for support. We thank N. Gompel, I. Kadow, and I. Solovei and reviewers for critical comments on the manuscript. We thank C. Bleese for help with graphical visualization. We thank the LSM and IMPRS graduate schools for supporting our students. Funding for this work was provided by MPG, DFG (CL490-1), and LMU-CAS.

Received: August 13, 2015
Revised: November 20, 2015
Accepted: December 16, 2015
Published: February 4, 2016

REFERENCES

1. Heisenberg, C.P., and Bellaïche, Y. (2013). Forces in tissue morphogenesis and patterning. Cell *153*, 948–962.
2. Varner, V.D., and Nelson, C.M. (2014). Cellular and physical mechanisms of branching morphogenesis. Development *141*, 2750–2759.
3. San Roman, A.K., and Shivdasani, R.A. (2011). Boundaries, junctions and transitions in the gastrointestinal tract. Exp. Cell Res. *317*, 2711–2718.
4. Dahmann, C., Oates, A.C., and Brand, M. (2011). Boundary formation and maintenance in tissue development. Nat. Rev. Genet. *12*, 43–55.
5. Batlle, E., Henderson, J.T., Beghtel, H., van den Born, M.M.W., Sancho, E., Huls, G., Meeldijk, J., Robertson, J., van de Wetering, M., Pawson, T., and Clevers, H. (2002). Beta-catenin and TCF mediate cell positioning in the intestinal epithelium by controlling the expression of EphB/ephrinB. Cell *111*, 251–263.
6. Pinto, D., and Clevers, H. (2005). Wnt control of stem cells and differentiation in the intestinal epithelium. Exp. Cell Res. *306*, 357–363.
7. Gibson, M.C., and Perrimon, N. (2005). Extrusion and death of DPP/BMP-compromised epithelial cells in the developing Drosophila wing. Science *307*, 1785–1789.
8. Shen, J., and Dahmann, C. (2005). Extrusion of cells with inappropriate Dpp signaling from Drosophila wing disc epithelia. Science *307*, 1789–1790.
9. Widmann, T.J., and Dahmann, C. (2009). Wingless signaling and the control of cell shape in Drosophila wing imaginal discs. Dev. Biol. *334*, 161–173.
10. Widmann, T.J., and Dahmann, C. (2009). Dpp signaling promotes the cuboidal-to-columnar shape transition of Drosophila wing disc epithelia by regulating Rho1. J. Cell Sci. *122*, 1362–1373.
11. Pallavi, S.K., Ho, D.M., Hicks, C., Miele, L., and Artavanis-Tsakonas, S. (2012). Notch and Mef2 synergize to promote proliferation and metastasis through JNK signal activation in Drosophila. EMBO J. *31*, 2895–2907.
12. Gandille, P., Narbonne-Reveau, K., Boissonneau, E., Randsholt, N., Busson, D., and Pret, A.M. (2010). Mutations in the polycomb group gene polyhomeotic lead to epithelial instability in both the ovary and wing imaginal disc in Drosophila. PLoS ONE *5*, e13946.
13. Bessa, J., Carmona, L., and Casares, F. (2009). Zinc-finger paralogues tsh and tio are functionally equivalent during imaginal development in Drosophila and maintain their expression levels through auto- and cross-negative feedback loops. Dev. Dyn. *238*, 19–28.
14. Aldaz, S., Morata, G., and Azpiazu, N. (2005). Patterning function of homothorax/extradenticle in the thorax of Drosophila. Development *132*, 439–446.
15. Beuchle, D., Struhl, G., and Müller, J. (2001). Polycomb group proteins and heritable silencing of Drosophila Hox genes. Development *128*, 993–1004.
16. Prober, D.A., and Edgar, B.A. (2000). Ras1 promotes cellular growth in the Drosophila wing. Cell *100*, 435–446.
17. Liu, X., Grammont, M., and Irvine, K.D. (2000). Roles for scalloped and vestigial in regulating cell affinity and interactions between the wing blade and the wing hinge. Dev. Biol. *228*, 287–303.
18. Worley, M.I., Setiawan, L., and Hariharan, I.K. (2013). TIE-DYE: a combinatorial marking system to visualize and genetically manipulate clones during development in Drosophila melanogaster. Development *140*, 3275–3284.
19. Perea, D., Molohon, K., Edwards, K., and Díaz-Benjumea, F.J. (2013). Multiple roles of the gene zinc finger homeodomain-2 in the development of the Drosophila wing. Mech. Dev. *130*, 467–481.
20. Gold, K.S., and Brand, A.H. (2014). Optix defines a neuroepithelial compartment in the optic lobe of the Drosophila brain. Neural Dev. *9*, 18.
21. Classen, A.K., Bunker, B.D., Harvey, K.F., Vaccari, T., and Bilder, D. (2009). A tumor suppressor activity of Drosophila Polycomb genes mediated by JAK-STAT signaling. Nat. Genet. *41*, 1150–1155.
22. Bell, G.P., and Thompson, B.J. (2014). Colorectal cancer progression: lessons from Drosophila? Semin. Cell Dev. Biol. *28*, 70–77.

23. Organista, M.F., and De Celis, J.F. (2013). The Spalt transcription factors regulate cell proliferation, survival and epithelial integrity downstream of the Decapentaplegic signalling pathway. Biol. Open *2*, 37–48.
24. Villa-Cuesta, E., González-Pérez, E., and Modolell, J. (2007). Apposition of iroquois expressing and non-expressing cells leads to cell sorting and fold formation in the Drosophila imaginal wing disc. BMC Dev. Biol. *7*, 106.
25. Shen, J., Dahmann, C., and Pflugfelder, G.O. (2010). Spatial discontinuity of optomotor-blind expression in the Drosophila wing imaginal disc disrupts epithelial architecture and promotes cell sorting. BMC Dev. Biol. *10*, 23.
26. Sawyer, J.M., Harrell, J.R., Shemer, G., Sullivan-Brown, J., Roh-Johnson, M., and Goldstein, B. (2010). Apical constriction: a cell shape change that can drive morphogenesis. Dev. Biol. *341*, 5–19.
27. Shyer, A.E., Tallinen, T., Nerurkar, N.L., Wei, Z., Gil, E.S., Kaplan, D.L., Tabin, C.J., and Mahadevan, L. (2013). Villification: how the gut gets its villi. Science *342*, 212–218.
28. Schuettengruber, B., and Cavalli, G. (2009). Recruitment of polycomb group complexes and their role in the dynamic regulation of cell fate choice. Development *136*, 3531–3542.
29. Martinez, A.M., Schuettengruber, B., Sakr, S., Janic, A., Gonzalez, C., and Cavalli, G. (2009). Polyhomeotic has a tumor suppressor activity mediated by repression of Notch signaling. Nat. Genet. *41*, 1076–1082.
30. Hafezi, Y., and Nystul, T. (2012). Advanced Techniques for Cell Lineage Labelling in Drosophila (John Wiley & Sons).
31. Myat, M.M., Isaac, D.D., and Andrew, D.J. (2000). Early genes required for salivary gland fate determination and morphogenesis in Drosophila melanogaster. Adv. Dent. Res. *14*, 89–98.
32. Pearson, J.C., Lemons, D., and McGinnis, W. (2005). Modulating Hox gene functions during animal body patterning. Nat. Rev. Genet. *6*, 893–904.
33. Bataillé, L., Augé, B., Ferjoux, G., Haenlin, M., and Waltzer, L. (2005). Resolving embryonic blood cell fate choice in Drosophila: interplay of GCM and RUNX factors. Development *132*, 4635–4644.
34. Johnston, L.A., Prober, D.A., Edgar, B.A., Eisenman, R.N., and Gallant, P. (1999). Drosophila myc regulates cellular growth during development. Cell *98*, 779–790.
35. van de Wetering, M., Cavallo, R., Dooijes, D., van Beest, M., van Es, J., Loureiro, J., Ypma, A., Hursh, D., Jones, T., Bejsovec, A., et al. (1997). Armadillo coactivates transcription driven by the product of the Drosophila segment polarity gene dTCF. Cell *88*, 789–799.
36. Morata, G. (2001). How Drosophila appendages develop. Nat. Rev. Mol. Cell Biol. *2*, 89–97.
37. Zoranovic, T., Grmai, L., and Bach, E.A. (2013). Regulation of proliferation, cell competition, and cellular growth by the Drosophila JAK-STAT pathway. JAK-STAT *2*, e25408.
38. Zimmerman, S.G., Thorpe, L.M., Medrano, V.R., Mallozzi, C.A., and McCartney, B.M. (2010). Apical constriction and invagination downstream of the canonical Wnt signaling pathway require Rho1 and Myosin II. Dev. Biol. *340*, 54–66.
39. Aegerter-Wilmsen, T., Heimlicher, M.B., Smith, A.C., de Reuille, P.B., Smith, R.S., Aegerter, C.M., and Basler, K. (2012). Integrating force-sensing and signaling pathways in a model for the regulation of wing imaginal disc size. Development *139*, 3221–3231.
40. Pastor-Pareja, J.C., and Xu, T. (2011). Shaping cells and organs in Drosophila by opposing roles of fat body-secreted Collagen IV and perlecan. Dev. Cell *21*, 245–256.
41. Eisenhoffer, G.T., and Rosenblatt, J. (2013). Bringing balance by force: live cell extrusion controls epithelial cell numbers. Trends Cell Biol. *23*, 185–192.
42. Fletcher, G.C., Elbediwy, A., Khanal, I., Ribeiro, P.S., Tapon, N., and Thompson, B.J. (2015). The Spectrin cytoskeleton regulates the Hippo signalling pathway. EMBO J. *34*, 940–954.
43. Levayer, R., and Moreno, E. (2013). Mechanisms of cell competition: themes and variations. J. Cell Biol. *200*, 689–698.
44. Haramis, A.P., Begthel, H., van den Born, M., van Es, J., Jonkheer, S., Offerhaus, G.J., and Clevers, H. (2004). De novo crypt formation and juvenile polyposis on BMP inhibition in mouse intestine. Science *303*, 1684–1686.
45. Fagotto, F., Winklbauer, R., and Rohani, N. (2014). Ephrin-Eph signaling in embryonic tissue separation. Cell Adhes. Migr. *8*, 308–326.
46. Milán, M., Pérez, L., and Cohen, S.M. (2002). Short-range cell interactions and cell survival in the Drosophila wing. Dev. Cell *2*, 797–805.
47. Paré, A.C., Vichas, A., Fincher, C.T., Mirman, Z., Farrell, D.L., Mainieri, A., and Zallen, J.A. (2014). A positional Toll receptor code directs convergent extension in Drosophila. Nature *515*, 523–527.
48. Landsberg, K.P., Farhadifar, R., Ranft, J., Umetsu, D., Widmann, T.J., Bittig, T., Said, A., Jülicher, F., and Dahmann, C. (2009). Increased cell bond tension governs cell sorting at the Drosophila anteroposterior compartment boundary. Curr. Biol. *19*, 1950–1955.
49. Röper, K. (2012). Anisotropy of Crumbs and aPKC drives myosin cable assembly during tube formation. Dev. Cell *23*, 939–953.

 ©2016 Elsevier Ltd All rights reserved

Current Biology, Volume *26*

Supplemental Information

Interface Contractility between Differently Fated Cells Drives Cell Elimination and Cyst Formation

Christina Bielmeier, Silvanus Alt, Vanessa Weichselberger, Marco La Fortezza, Hartmann Harz, Frank Jülicher, Guillaume Salbreux, and Anne-Kathrin Classen

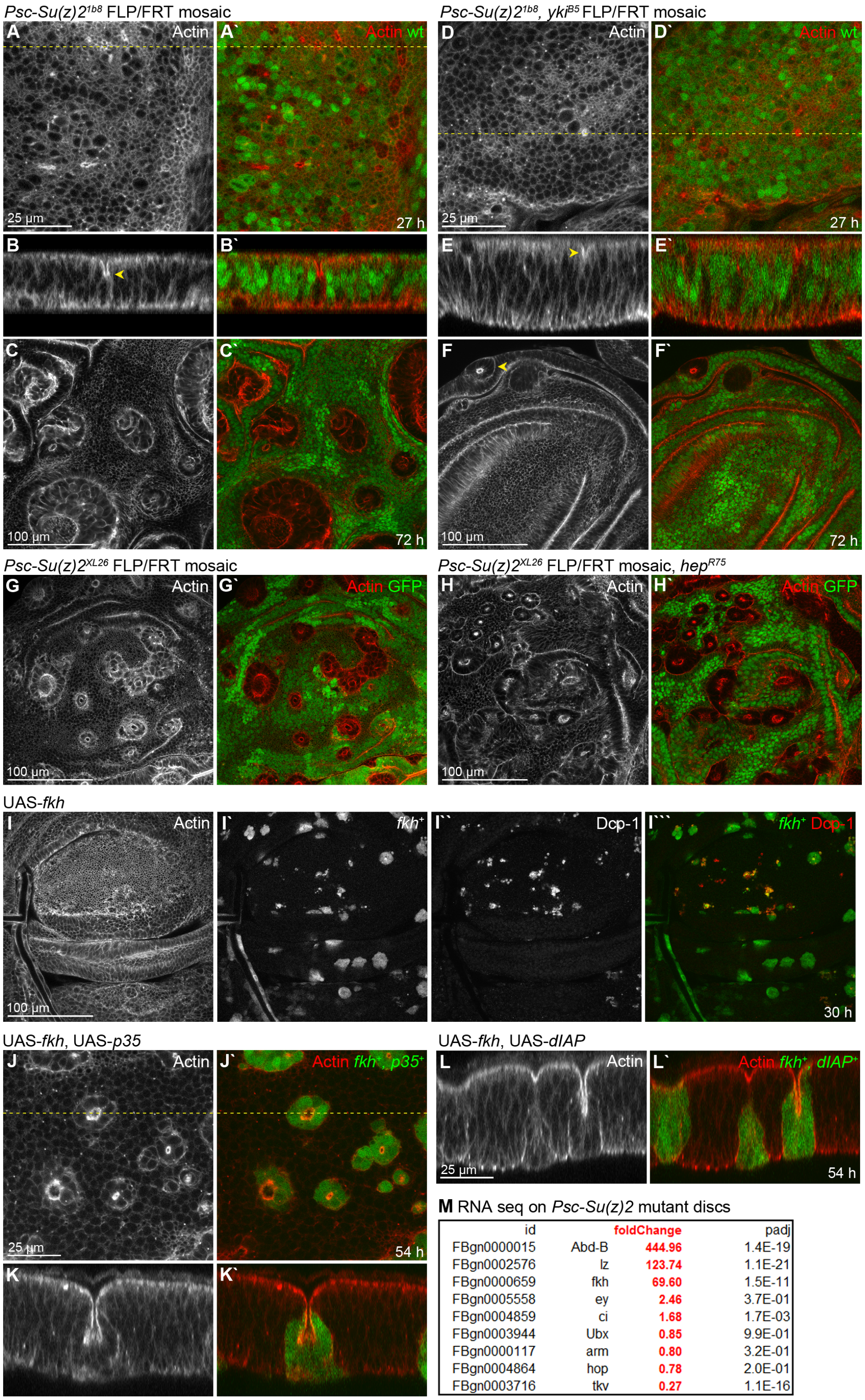

M RNA seq on *Psc-Su(z)2* mutant discs

id		foldChange	padj
FBgn0000015	Abd-B	**444.96**	1.4E-19
FBgn0002576	lz	**123.74**	1.1E-21
FBgn0000659	fkh	**69.60**	1.5E-11
FBgn0005558	ey	**2.46**	3.7E-01
FBgn0004859	ci	**1.68**	1.7E-03
FBgn0003944	Ubx	**0.85**	9.9E-01
FBgn0000117	arm	**0.80**	3.2E-01
FBgn0004864	hop	**0.78**	2.0E-01
FBgn0003716	tkv	**0.27**	1.1E-16

Figure S1

Overgrowth, JNK-signaling or induction of apoptosis cannot account for cyst formation by misspecified cells

Related to Figure 1

(A-F) Wing imaginal disc containing *Psc-Su(z)2*^{1b8} clones (A-C) or clones mutant for both *Psc-Su(z)2*^{1b8} and *yki*^{B5} (D-F). Wild type cells (wt) are marked by GFP (green in (A'-F')). Actin is shown in red (A'-F') or grey (A-F). Confocal xy-section at 30 h (A, D) and reconstructed xz cross-sections (B, E) at position indicated by dotted line in (A, D). Arrowheads are pointing to invaginating clones. (C, F) Confocal xy-section at 72 h after clone induction. *Psc-Su(z)2*^{1b8} mutant clones in (C) undergo extensive proliferation leading to big cysts at 72 h after clone induction. The *yki*^{B5} allele prevents proliferation and survival of *Psc-Su(z)2*^{1b8} mutant cells (F) but not invagination and cyst formation (arrowheads in E, F).

(G, H) *Psc-Su(z)2*^{XL26} clones in a wild type wing disc (G) and in a wing disc hemizygous for a *hep*^{R75} allele (H). Confocal xy-sections at 54 h after clone induction are shown. *Psc -Su(z)2*^{XL26} mutant cells are marked by lack of GFP (green) (G', H'). Actin is shown in red (G',H') or grey (G,H). *hep*^{R75} prevents activation of JNK signaling in imaginal discs but does not interfere with cyst formation.

(I) Pouch region containing *fkh*-expressing clones (grey in **I**', green in **I**''') at 30 h after clone induction showing extensive activation of apoptosis, as revealed by the presence of the cleaved effector Caspase, Death Caspase-1 (Dcp-1) (grey in **I**'', red in **I**'''). Actin is shown in (**I**).

(J-L) Pouch region containing *fkh*-expressing clones (green in J'-M') that also express the anti-apoptotic factors *p35* (J, K) or *dIAP1* (L) at 54 h after clone induction. Confocal xy-sections (J) and reconstructed xz cross-sections (K, L) at position indicated by dotted line in (J) are shown. Actin is shown in grey (J-L) or red (J'-L'). Cell-autonomous inhibition of apoptosis by *p35* or by *dIAP1* does not prevent cyst formation.

(M) RNA-Seq analysis of *Psc-Su(z)2*^{XL26} mutant wing discs [S1]. *Abd-B* and *lz* are strongly transcriptionally upregulated in *Psc-Su(z)2*^{XL26} mutant cells. *Ubx*, *ci*, *arm* and other signaling components driving cyst formation are not upregulated in *Psc-Su(z)2*^{XL26} mutant discs. RNA-Seq data was validated by real-time PCR experiments [S2] (not shown).

Length of scale bars is indicated in individual images. Related data sets are shown at equal magnifications.

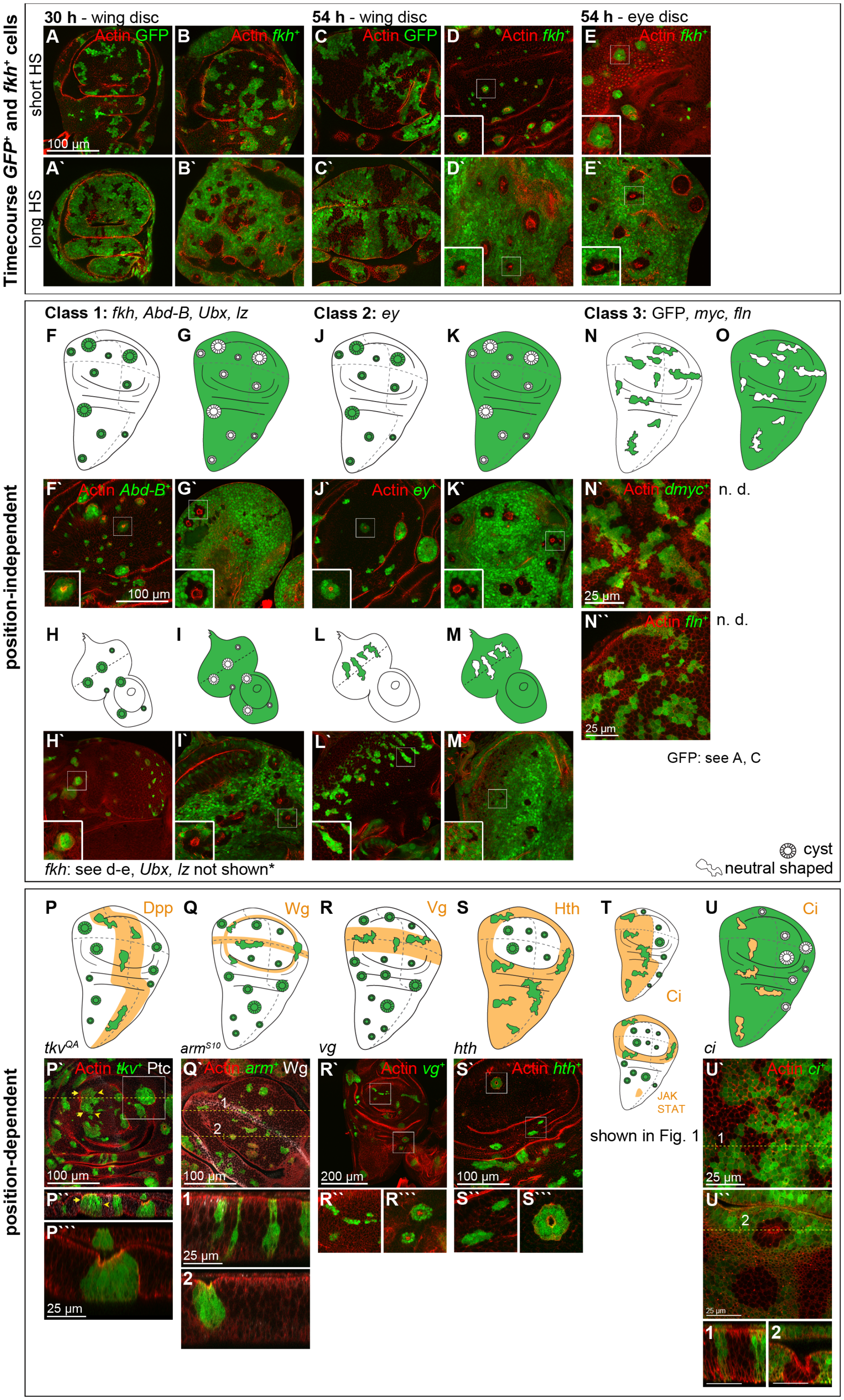

Timecourse GFP+ and fkh+ cells
30 h - wing disc
54 h - wing disc
54 h - eye disc
short HS
long HS
Actin GFP
Actin fkh+
100 µm
Class 1: fkh, Abd-B, Ubx, lz
Class 2: ey
Class 3: GFP, myc, fln
position-independent
Actin Abd-B+
Actin ey+
Actin dmyc+
Actin fln+
n. d.
25 µm
GFP: see A, C
fkh: see d-e, Ubx, lz not shown*
cyst
neutral shaped
position-dependent
Dpp
Wg
Vg
Hth
Ci
JAK STAT
tkvQA
armS10
vg
hth
ci
Actin tkv+ Ptc
Actin arm+ Wg
Actin vg+
Actin hth+
Actin ci+
shown in Fig. 1
200 µm

Figure S2

Ectopic expression of cell fate specifying transcription factors induces cyst formation

Related to Figures 1 and 2

(A-E) Confocal xy-sections of pouch region containing GFP-expressing (A, C) and *fkh*-expressing (B, D) clones as well as eye discs containing *fkh*-expressing clones (E) at 30 h (A, B) and 54 h (C-E) after clone induction using a short (A-E) and long heat-shock (A`-E`). Actin is shown in red, clones in green. White boxes in D, E frame examples of cyst-forming clones shown in small insets.

(F-I) Example of transcription factors that form cysts upon overexpression in wing and eye imaginal disc (*fkh*, *Abd-B*, *Ubx* and *lz*; **lz* only tested in the wing disc). F-I show schematic representation of position-independent cyst formation in the wing (F, G) and eye (H, I) where cysts are formed by misspecified cells (green; F, H) or wild type cells (white; G, I). F`-I` show confocal xy-sections of pouch region (F`, G`) and eye discs (H`, I`) containing *Abd-B*-expressing clones (green) 54 h after clone induction using a short (F`, H`) and long (G`, I`) heat-shock. Actin is shown in red.

(J-M) Overexpression of *ey* leads to cysts in the wing discs but rarely in the eye disc. Schematic representation is shown for wing disc (J, K) and eye discs (L, M). J`-M` show confocal xy-section of pouch region (J`, K`) and eye discs (L`, M`) containing *ey*-expressing clones (green) at 54 h after clone induction using a short (J`, L`) and long (K`, M`) heat-shock. Actin is shown in red.

(N-O) Overexpression of GFP, *myc* and *fln* does not lead to cyst formation. Schematic wing imaginal discs are shown in N, O. Confocal xy-sections of pouch region containing *dMyc*-expressing clones (N`) and *flightin* (*fln*)-expressing clones (N``)(green) at 54 h after clone induction. Actin is shown in red.

(P-U) Examples of position-dependent cyst formation by activation of transcription factors that show a spatially defined expression pattern in the wing imaginal disc. P-U show a schematic representation of wing imaginal discs indicating individual endogenous expression patterns (orange) and regions of cyst formation.

(P`- P```) Confocal xy-section (P`) and xz cross-section (P``, P```) of pouch region containing clones that express a dominant-active version of the *thickveins* (*tkv*) receptor 54 h after clone induction. Actin is shown in red; Ptc staining visualizes the A/P boundary (grey). Dashed line in P` indicates position of cross-section shown in P``. White box frames region of xz cross-section in P```. Note the rough clone boundaries in high Dpp-signaling regions (arrowhead in P`). In contrast, boundaries from the same clone dramatically smoothen (arrow) at basal contacts when they locate outside of Dpp-signaling domains.

**(Q`)** Confocal xy-section (Q`) and xz-cross section (1, 2) of pouch region containing clones (green) that express a dominant-active *armadillo* (*arm*) at 54 h after clone induction. Actin is shown in red, Wg staining in white. Dashed yellow lines indicate position of cross-sections shown in 1 and 2. Note that cysts form in regions of low endogenous Wg signaling (G, H) whereas clones maintain neutral shapes in regions of high Wg signaling.

(R`- R```, S`-S```) Confocal xy-section of pouch region containing clones (green) that express the transcription factor *vestigial* (*vg*, R`-R```) or *homothorax* (*hth*, S`-S```) at 54 h after clone induction. Actin is shown in red. White boxes frame regions, which are shown at higher magnification below. Note the difference in clone smoothening.

**(U`- U``)** Position-dependent cyst formation by wild type cells is illustrated by clonal overexpression of *ci*. Confocal xy-sections (U`, U``) and xz cross-sections (1, 2) of *ci*-expressing clones (green) in the posterior (U`) and anterior (U``) compartment at 54 h after clone induction using a long heat-shock. *ci*-expressing cells are marked by GFP (green), Actin is shown in red. Dashed yellow lines indicate position of cross-sections shown in 1 and 2.

Length of scale bars is indicated in individual images. Related data sets are shown at equal magnifications.

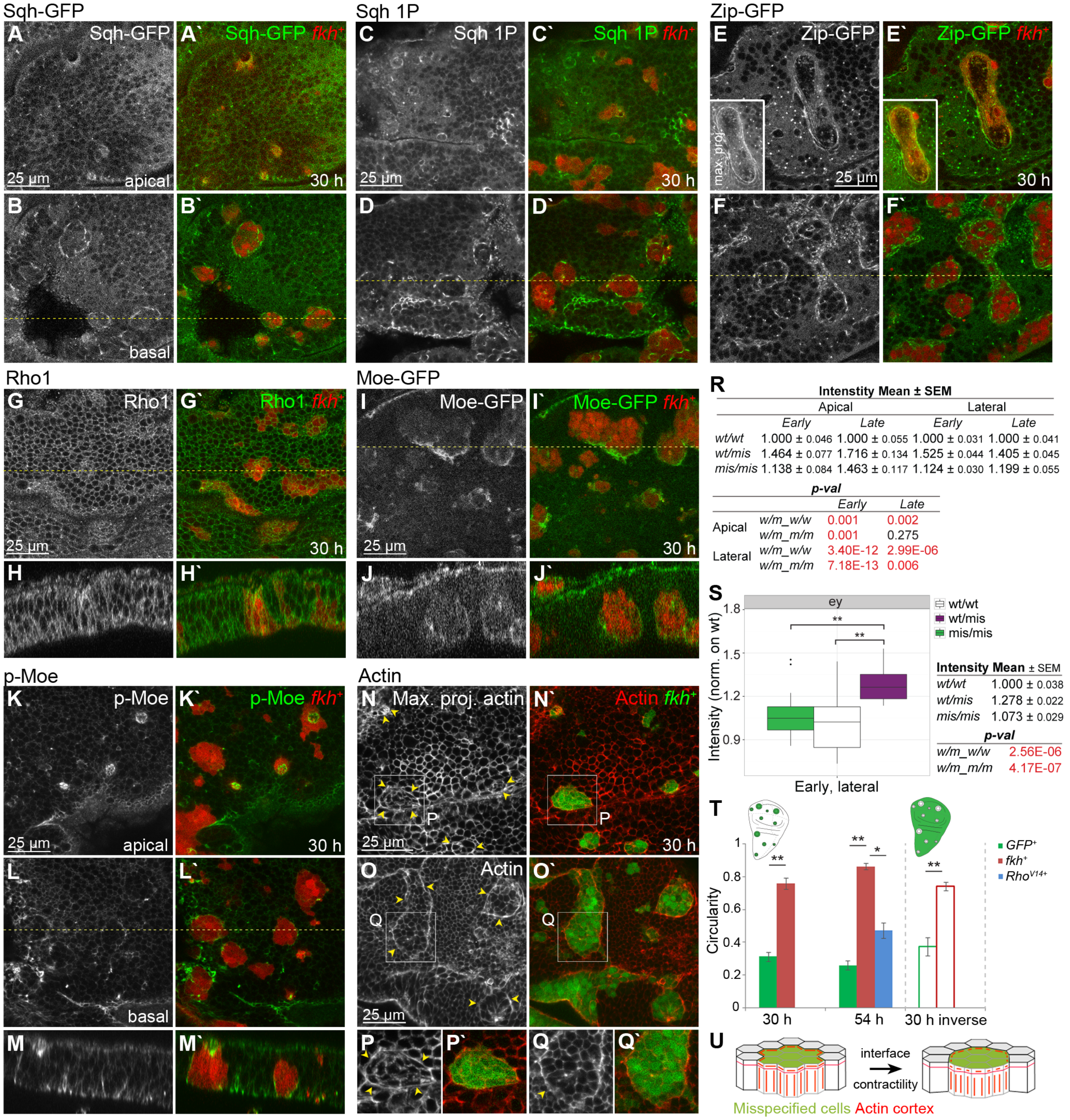

Figure S3

The contractile actomyosin machinery is recruited to the MWI leading to minimization of MWI contact area

Related to Figure 2

(A-F) Single xy-sections through apical (A, C, E) and basolateral (B, D, F) levels of a wing disc pouch containing *fkh*-expressing clones marked by GFP (red in A'-F') at 30 h after clone induction. Additionally, the disc expresses a non-muscle myosin II regulatory light chain GFP-fusion protein (Sqh-GFP, grey in A, B, green in A', B'), stained for the activated phosphorylated form of Sqh (Sqh 1P, grey in C, D, green in C', D') or expressing a non-muscle myosin II heavy chain GFP-fusion protein (Zip-GFP, grey in E, F, green in E',

F'). The dotted lines in the basolateral xy-sections (B, D, F) indicate positions of xz cross-sections shown in Figure 2G-I. Insets in (E) are maximum projections of all apical sections. Note the additional strong enrichment of actomyosin components at basolateral MWI's (B, D, F).

(G-J) Single xy-sections (G, I) and reconstructed xz cross-sections (H, J) of a wing imaginal disc containing *fkh*-expressing clones marked by GFP (red in G'-J') at 30 h after clone induction. Additionally, the disc is stained for the GTPase Rho1 (grey in G, H and green in G', H') or expresses a Moesin-GFP-fusion protein (Moe-GFP) (grey in I, J and green in I', J'). The dotted lines in (G, I) indicate positions of xz cross-sections (H, J). Whereas Moe-GFP enriches at lateral MWI's, Rho1 does not specifically enrich there.

(K-Q) Single xy-sections through apical (K, N, P) and basolateral (L, O, Q) levels of a wing disc pouch containing *fkh*-expressing clones marked by GFP (red in K'-M', green in N'-Q') at 30 h after clone induction. Additionally, the disc was stained for the phosphorylated form of Moesin (p-Moe, green in K'-M', grey in K-M) or stained with phalloidin (red in N'-Q' and grey in N-Q) to visualize accumulation of actin at apical adherens junctions (N, P) and basal integrin junctions (O, Q) at the MWI (arrowheads).

The dotted line in (L) indicates positions of xz cross-sections (M). Boxes in (N, O) frame regions shown at higher magnification in (P, Q).

(R) Normalized mean intensities and SEM of actin intensity measurements at apical and lateral domains in discs containing *fkh*-expressing cells (top table corresponds to data visualized as box plots in Fig. 2E, F). Data of experimental conditions where *fkh*-expressing clones (10 min heat-shock) and wild type cells (25 min heat-shock) would form cysts were pooled to reflect the mirror symmetry of the cyst-forming conditions. Actin intensities at interfaces between wild type cell (wt/wt), between misspecified *fkh*-expressing cells (mis/mis) and at the interface between wild type and *fkh*-expressing cell (wt/mis) are shown for early stages (30 h) and late stages (54 h) after induction of *fkh*-expressing cells.

Significance of differences between data sets (table below) was tested by applying a two-tailed WSR-test. Calculated p-values are shown. Red = p-val < 0.01. Number of clones analyzed for basolateral actin intensity: 10min heat-shock, 30 h, n=49 clones; 10min heat-shock, 54 h, n=17 clones; 25 min heat-shock, 30h, n=38 clones; 25 min heat-shock, 54 h, n=14 clones. Number of clones analyzed for apical actin intensity: 10min heat-shock, 30h, n=15 clones; 10min heat-shock, 54 h, n=7 clones; 25 min heat-shock, 30 h, n=10 clones; 25 min heat-shock, 54 h, n=3 clones. Lack of statistically significant differences for apical actin intensities at late stages are due to mild cell-autonomous enrichment of actin in *fkh*-expressing cells.

(S) Actin intensity at basolateral interfaces between misspecified *ey*-expressing cells (mis/mis), wild type cells (wt/wt), and between wild type and misspecified *ey*-expressing cells (wt/mis) 30 h after clone induction. Data of experimental conditions, where *ey*-expressing clones (10min heat-shock, n=22 clones) and wild type clones (25 min heat-shock, n=10 clones) were pooled to reflect the mirror symmetry of cyst-forming conditions. A box plot and a table displaying mean and SEM of the data are shown. Significance of differences between data sets was tested by applying a two-tailed WSR test. ** = p-val < 0.001. Calculated p-values are shown in the table. Red = p-val < 0.01.

(T) Clone circularity represented in a bar graph and schematic representation of conditions for which clone circularity was determined. Solid colored bars represent circularity for neutral wild type clones expressing GFP (green), and *fkh*-expressing clones (red) or Rho^{V14}, *p35*-expressing clones (blue) giving rise to cysts, early (30 h) and late (54 h) after clone induction. White bars represent circularity for wild type clones if surrounded by neutral GFP-expressing cells (green outline) or by *fkh*-expressing cells (red outline) at early stages after induction (30 h). Number of clones analyzed: GFP 30 h (n=10), *fkh* 30 h (n=10), GFP 54 h (n=6), *fkh* 54 h (n=11), *Rho*^{V14} 54 h (n=11), GFP inv 30 h (n=9), *fkh* inv 30 h (n=9). Inv (inverse) describes

conditions where wild type cells were surrounded by either GFP or *fkh*-expressing cells. Graph represents the mean and SEM of the data. Significance of differences between data sets was tested by applying a two-tailed WMW test. * = p-val < 0.01, ** = p-val < 0.001

(U) Schematic representation of the effect of actin recruitment to the MWI (red) leading to increased apical and basolateral MWI contractility, as well as MWI minimization and decreased roughness.

Length of scale bars is indicated in individual images. Related data sets are shown at equal magnifications.

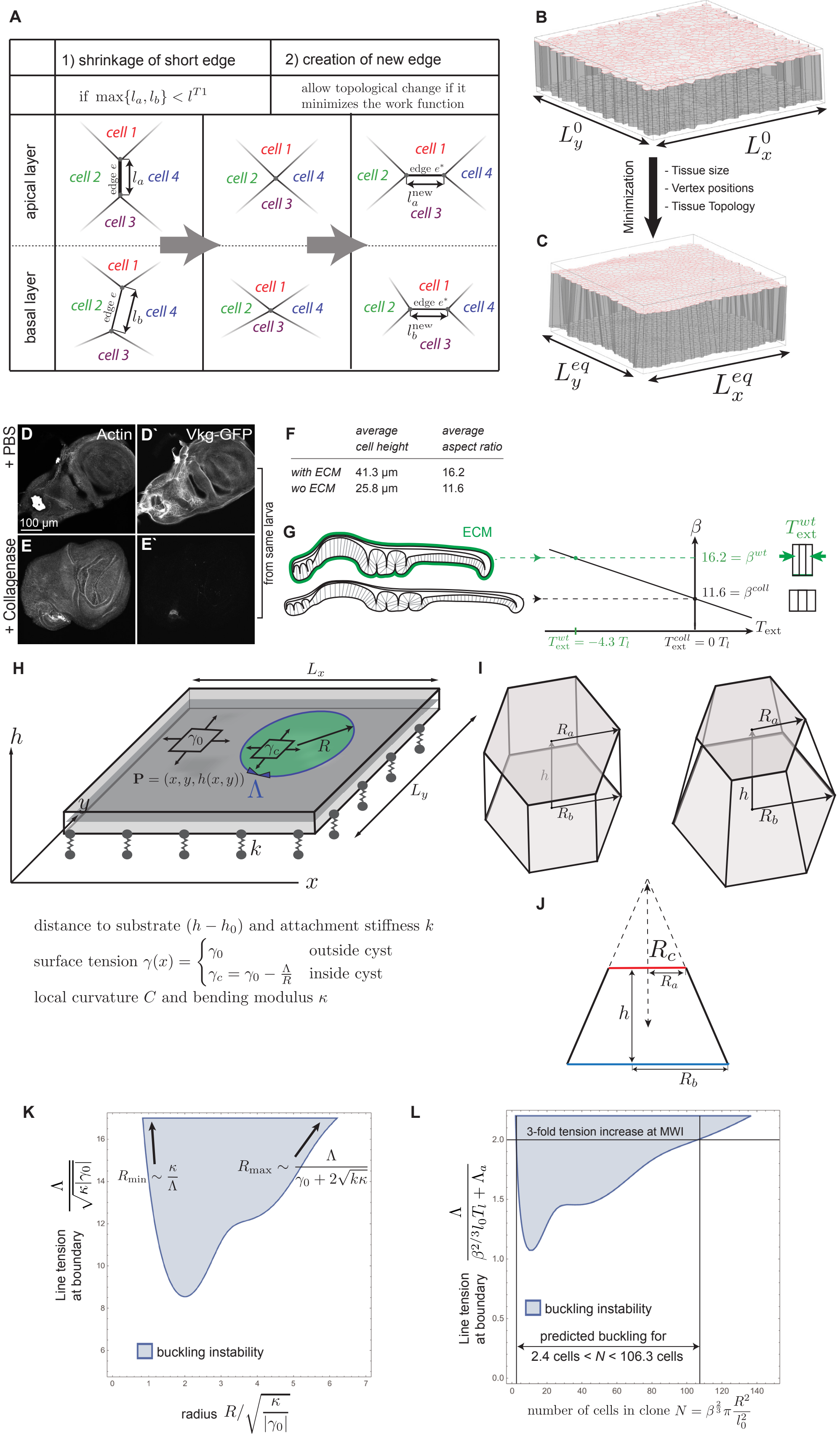
A
1) shrinkage of short edge
2) creation of new edge
if $\max\{l_a, l_b\} < l^{T1}$
allow topological change if it minimizes the work function
apical layer
basal layer
cell 1
cell 2
cell 3
cell 4
B
L_y^0
L_x^0
Minimization
- Tissue size
- Vertex positions
- Tissue Topology
C
L_y^{eq}
L_x^{eq}
+ PBS
+ Collagenase
D Actin
D` Vkg-GFP
100 µm
E
E`
from same larva
F
with ECM 41.3 µm 16.2
wo ECM 25.8 µm 11.6
average cell height
average aspect ratio
G
ECM
$16.2 = \beta^{wt}$
$11.6 = \beta^{coll}$
$T^{wt}_{ext} = -4.3\ T_l$
$T^{coll}_{ext} = 0\ T_l$
T_{ext}
H
L_x
L_y
$\mathbf{P} = (x, y, h(x, y))$
Λ
R
k
distance to substrate $(h - h_0)$ and attachment stiffness k
surface tension $\gamma(x) = \begin{cases} \gamma_0 & \text{outside cyst} \\ \gamma_c = \gamma_0 - \frac{\Lambda}{R} & \text{inside cyst} \end{cases}$
local curvature C and bending modulus κ
I
R_a
R_b
h
J
R_c
K
$R_{min} \sim \frac{\kappa}{\Lambda}$
$R_{max} \sim \frac{\Lambda}{\gamma_0 + 2\sqrt{k\kappa}}$
Line tension at boundary $\frac{\Lambda}{\sqrt{\kappa|\gamma_0|}}$
radius $R/\sqrt{\frac{\kappa}{|\gamma_0|}}$
buckling instability
L
3-fold tension increase at MWI
Line tension at boundary $\frac{\Lambda}{\beta^{2/3} l_0 T_l + \Lambda_a}$
buckling instability
predicted buckling for
2.4 cells < N < 106.3 cells
number of cells in clone $N = \beta^{\frac{2}{3}} \pi \frac{R^2}{l_0^2}$

Figure S4
3D Vertex Model implementation and continuum model description of cyst formation
Related to Figure 3 and 4

(A) A T_1 transition in the 3D Vertex Model represents an exchange of cell neighbors, where topological transitions take place both on apical and basal networks.
1) If an edge's (e) apical and basal lengths (l_a, l_b) fall beyond a predefined threshold (l_{T1}), the edge is replaced by a vertex.
2) A new edge replacing the vertex is then chosen as described in the Suppl. Information (e^*). The initial apical and basal lengths of e^* of the new edge are set to l_a^{new} and l_b^{new} respectively.
(B, C) To generate randomized wild type tissues, a 2D Voronoi tesselation for randomly distributed points inside a periodic box with sizes L_x^0 and L_y^0 was created (B). This initial tissue is relaxed with respect to the size of the periodic box (L_x^{eq}, L_y^{eq}), to the positions of the vertices inside the box and with respect to topological changes (C).
(D, E) Wing imaginal discs siblings expressing Vkg-GFP (grey in D`, E`) and stained for Actin (grey in D, E). One sibling disc was treated for 12 min with collagenase (E), the other with PBS only (D). Note absence of Vkg-GFP signal after collagenase treatment (E`) and increase in disc area (compare E with D).
(F) The aspect ratio β of a cell is defined to be the ratio between its height and the square root of its apical surface area. After collagenase treatment pouch cells take on a more cuboidal shape and their average aspect ratio decreases from 16.2 to 11.6.
(G) Tissue aspect ratio as a function of the external tension applied on the tissue, if all mechanical parameters other than T_{ext} are chosen as in Table 1. If the wing disc is under external compression due to the ECM, the compression is removed by collagenase treatment. The aspect ratio of uncompressed cells relates to the relative strength of the lateral surface tension. The aspect ratio of compressed cells can then be used to estimate the extent of external compression.
(H) In a continuum description, the tissue is described as a thin membrane, assuming that the height of the tissue is small compared to the length scale of deformations. The position of points in the tissue mid-plane are given in the Monge gauge by their heights $h(x, y)$. The membrane representing the tissue has a bending rigidity κ penalizing deviations of the local mean curvature C from the spontaneous curvature C_0, is subjected to an external surface tension γ_0, and is attached by elastic linkers with elastic modulus k to the underlying ECM, represented as a flat surface at $z = 0$. The misspecified clone with radius R is surrounded by the MWI, which exerts an effective line tension Λ, inducing a size-dependent compression γ_c acting on the clone. The resulting work function is given in Eq. 24.
(I) The continuous model parameters are related to the 3D vertex model parameters by considering a packing of identical hexagonal cells with apical and basal radii R_a and R_b and height h.
(J) For a 3D packing of identical cells, the local radius of curvature of the tissue mid-plane R_c can be related to the cell's apical and basal radii R_a and R_b.
(K) Stability diagram of a circular tissue region clamped at its boundary, under negative surface tension γ_0 and subjected to an additional line tension acting along its boundary. The blue region depicts the regime of unstable tissue sizes for normalized strength of the boundary effect $\Lambda/\sqrt{\kappa|\gamma_0|}$, and fixed attachment stiffness $k/(\gamma_0^2/\kappa) = 3$.
(L) Stability diagram of a circular tissue region clamped at its boundary and subjected to a line tension along its boundary, with parameters of the continuum theory related to vertex model parameters (section 4.5 of Suppl. Information). Parameters ($\kappa/(T_l l_0^2) \simeq 0.34$, $\gamma_0/T_l \simeq -4.2$, $k/(T_l/l_0^2) \simeq 64$) are obtained by taking

vertex model parameters of Table 1 in the Suppl. Information and estimating the corresponding continuum theory parameters. The black line indicates a 3-fold increase in apical line and lateral surface tension at the MWI.

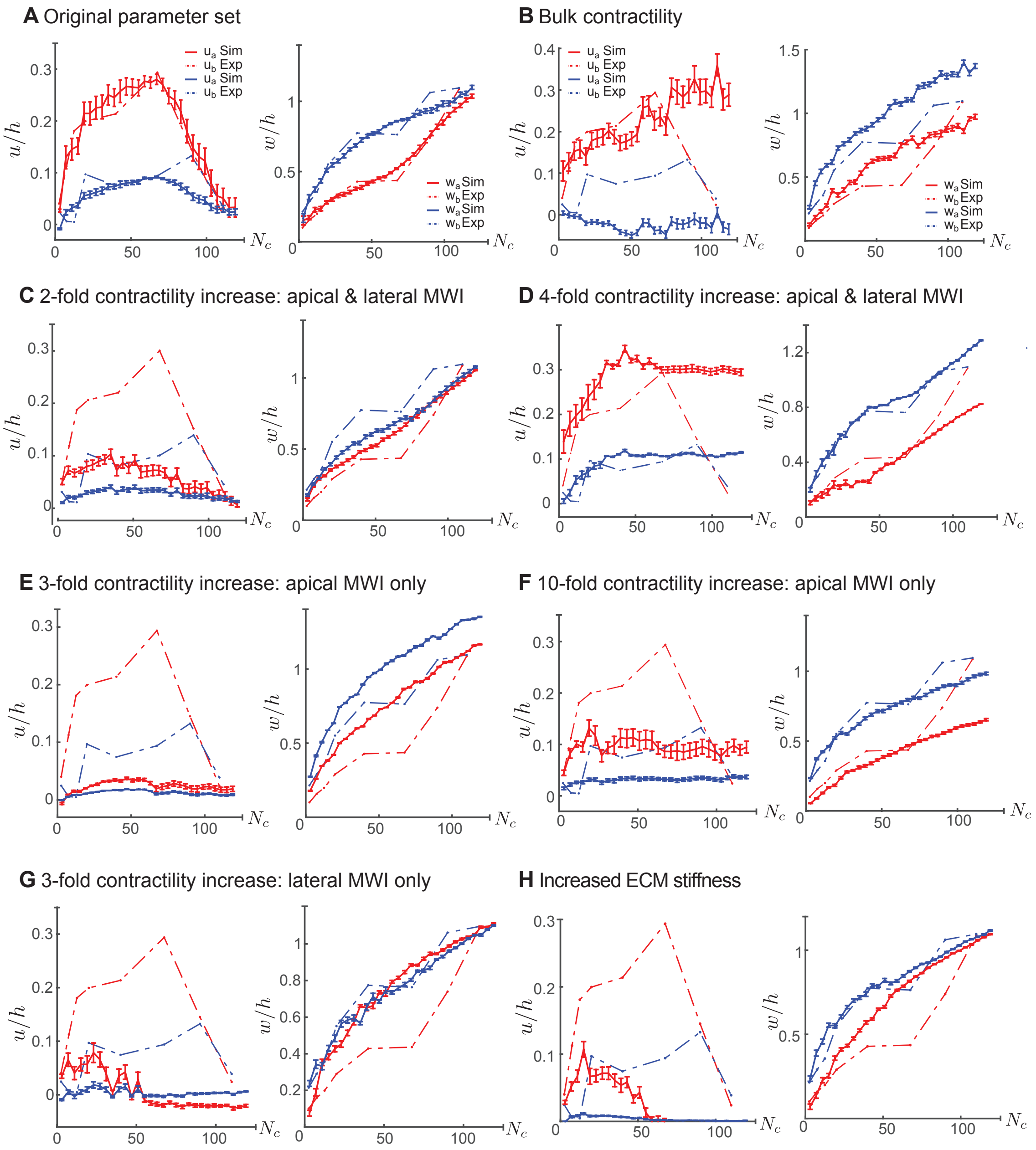

Figure S5

Parameter variation for cyst simulations in the 3D vertex model

Related to Figure 5

Variations of mechanical parameters around parameters fitted to experimental observations. Mechanical parameters for each set of simulations are the same as in Figure 4, except for indicated parameter changes and values marked by an asterisk in equations below. Graphs show mean and SEM of 15 simulations. Graphs illustrate experimental (dotted line) and simulated (continuous line) deformation of apical (red) or basal (blue) cyst surfaces with respect to clone size. Parameters u_a, u_b, w_a and w_b are illustrated in a schematic in Fig. 5E, and are normalised with the height h of the surrounding tissue.

(A) Original parameter set as given in Table 1 in the Supplementary Modeling Procedures with a 3-fold

increase in apical line and lateral surface tension at the MWI.

(B) Simulations with 3-fold increased lateral surface tension in misspecified cells only. Large clones are strongly deformed by such a perturbation, in disagreement with experimental observations.
$(T_l^{(mis/mis,*)} = 3T_l^{(wt/wt)}$, $T_l^{(wt/mis,*)} = 2T_l^{(wt/wt)})$

(C) Simulations with increased apical line and lateral surface tensions at the MWI by a factor of 2. The clone deformation is weaker than observed in experiments.
$(T_l^{(wt/mis,*)} = 2T_l^{(wt/wt)}$, $\Lambda_a^{(wt/mis,*)} = 2\Lambda_a^{(wt/wt)})$

(D) Simulations with increased apical line and lateral surface tensions at the MWI by a factor of 4. The clone deformation is larger than observed in experiments.
$(T_l^{(wt/mis,*)} = 4T_l^{(wt/wt)}$, $\Lambda_a^{(wt/mis,*)} = 4\Lambda_a^{(wt/wt)})$

(E-F) Simulations with 3 and 10 fold increased apical line tension alone at the MWI, and no increase in lateral surface tension. Experimentally observed apical indentation is not recapitulated in these conditions. This suggests that the contribution of contractility at basolateral interfaces is necessary to describe the observed clone shapes.
(E: $T_l^{(wt/mis,*)} = T_l^{(wt/wt)}$, $\Lambda_a^{(wt/mis,*)} = 3\Lambda_l^{(wt/wt)}$;
$F : T_l^{(wt/mis,*)} = T_l^{(wt/wt)}$, $\Lambda_a^{(wt/mis,*)} = 10\Lambda_a^{(wt/wt)}$)

(G) Simulations with 3-fold increase in lateral surface tension at the MWI, and no increase in apical line tension. Experimentally observed apical indentation and apicobasal asymmetry is not recapitulated in these conditions.This indicates that the additional symmetry breaking by the increase in apical line tension at the MWI is required to explain the observed deformations.
$(T_l^{(wt/mis,*)} = 3T_l^{(wt/wt)}$, $\Lambda_a^{(wt/mis,*)} = \Lambda_a^{(wt/wt)})$

(H) Simulations with increased stiffness of attachment to the ECM. With increasing attachment stiffness the apical and basal surfaces deform less.
$(k^* = 2k)$

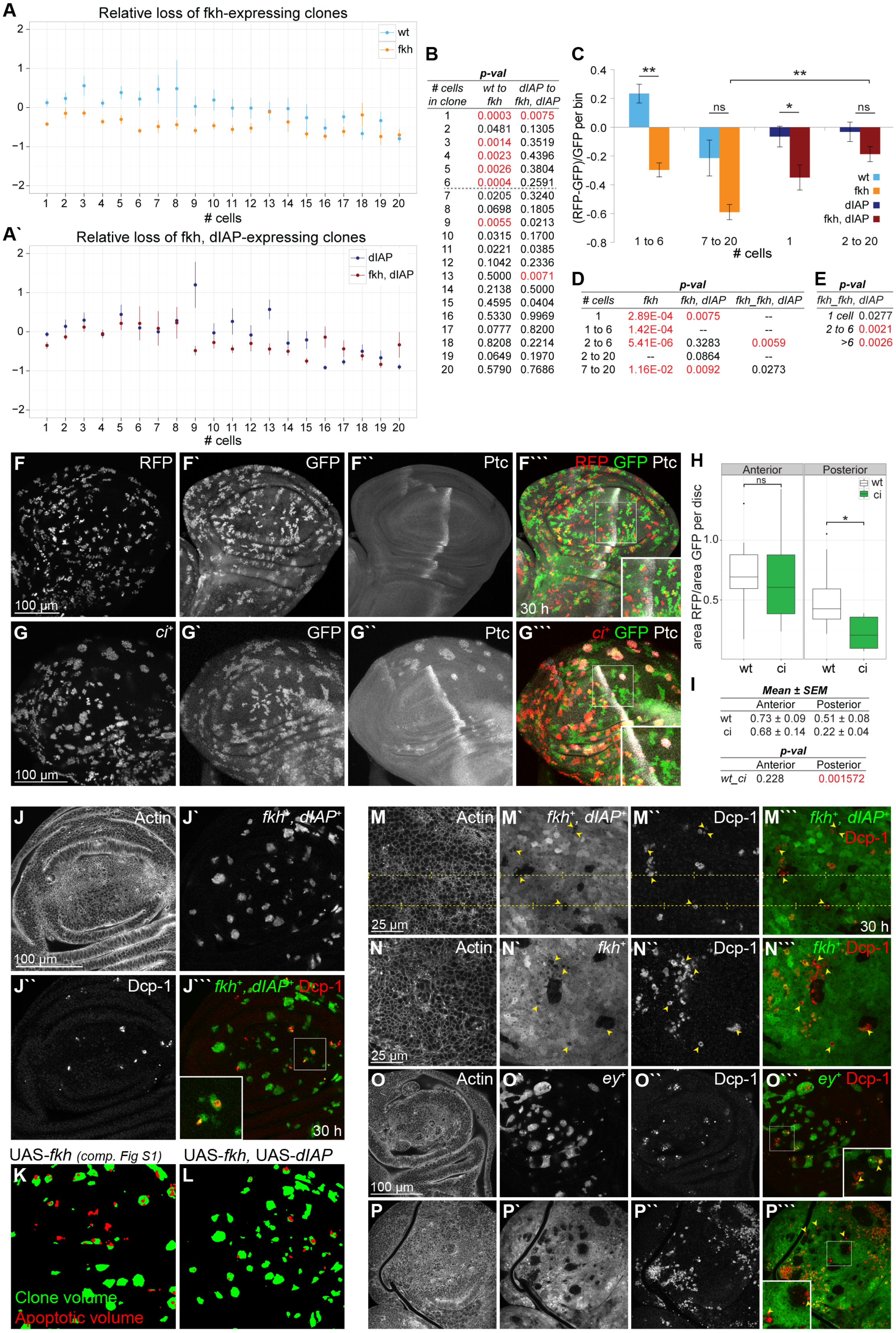
A
Relative loss of fkh-expressing clones
wt
fkh
cells
A`
Relative loss of fkh, dIAP-expressing clones
dIAP
fkh, dIAP
cells
B
p-val
cells in clone | wt to fkh | dIAP to fkh, dIAP
1 0.0003 0.0075
2 0.0481 0.1305
3 0.0014 0.3519
4 0.0023 0.4396
5 0.0026 0.3804
6 0.0004 0.2591
7 0.0205 0.3240
8 0.0698 0.1805
9 0.0055 0.0213
10 0.0315 0.1700
11 0.0221 0.0385
12 0.1042 0.2336
13 0.5000 0.0071
14 0.2138 0.5000
15 0.4595 0.0404
16 0.5330 0.9969
17 0.0777 0.8200
18 0.8208 0.2214
19 0.0649 0.1970
20 0.5790 0.7686
C
(RFP-GFP)/GFP per bin
1 to 6
7 to 20
1
2 to 20
cells
wt
fkh
dIAP
fkh, dIAP
ns
D
p-val
cells | fkh | fkh, dIAP | fkh_fkh, dIAP
1 2.89E-04 0.0075 --
1 to 6 1.42E-04 -- --
2 to 6 5.41E-06 0.3283 0.0059
2 to 20 -- 0.0864 --
7 to 20 1.16E-02 0.0092 0.0273
E
p-val
fkh_fkh, dIAP
1 cell 0.0277
2 to 6 0.0021
>6 0.0026
F RFP
F` GFP
F`` Ptc
F``` RFP GFP Ptc
100 µm
30 h
G ci+
G` GFP
G`` Ptc
G``` ci+ GFP Ptc
100 µm
H
Anterior
Posterior
wt
ci
ns
area RFP/area GFP per disc
I
Mean ± SEM
| Anterior | Posterior
wt 0.73 ± 0.09 0.51 ± 0.08
ci 0.68 ± 0.14 0.22 ± 0.04
p-val
| Anterior | Posterior
wt_ci 0.228 0.001572
J Actin
J` fkh+, dIAP+
J`` Dcp-1
J``` fkh+, dIAP+ Dcp-1
100 µm
30 h
UAS-fkh (comp. Fig S1)
UAS-fkh, UAS-dIAP
K
L
Clone volume
Apoptotic volume
M Actin
M` fkh+, dIAP+
M`` Dcp-1
M``` fkh+, dIAP+ Dcp-1
25 µm
30 h
N Actin
N` fkh+
N`` Dcp-1
N``` fkh+ Dcp-1
25 µm
O Actin
O` ey+
O`` Dcp-1
O``` ey+ Dcp-1
100 µm
P
P`
P``
P```

Figure S6

Misspecified cells are eliminated from imaginal discs by MWI contractility

Related to Figure 6

(A) Relative loss of (A) *fkh* and (A') *fkh*, *dIAP1*-expressing RFP-positive clones compared to control RFP-positive clones ((A) wt and (A') *dIAP1*-expressing clones, respectively) in Tie-Dye wing discs. RFP clones expressed either RFP alone (light blue), *fkh* (light red), *dIAP1* (dark blue) or *fkh*, *dIAP1* (dark red). For each disc, the number of control GFP-expressing clones was subtracted from the number of RFP-clones and normalized to the number of GFP-clones for the respective clone size bin. This analysis utilizes the strength of the Tie-Dye system as the data is normalized to intrinsic GFP-positive control clones for each clone size bin and genotype.

Graphs represent the mean and SEM of the data (n=10 discs for wt, *fkh*, *fkh*, *dIAP1* each; n=8 discs for *dIAP1*). Number of GFP clones analyzed: GFP clones: wt (n=1738), *fkh* (n=1714), *dIAP1* (n=1431), *fkh*, *dIAP1* (n=1572). RFP clones: wt (n=1861), *fkh* (n=997), *dIAP1* (n=1381), *fkh*, *dIAP1* (n=1221)

(B) Table listing p-values calculated to test for statistical significant difference in relative loss of RFP clones (A, A') per clone size bin utilizing a one-tailed WMW-test. Red = p-val < 0.01. The analysis suggests that *fkh*-expressing clones of less than 6 cells and *fkh*, *dIAP1*-expressing clones of 1 cell are significantly under-represented in the data set. We thus chose these thresholds for further analysis.

(C) Analysis of relative loss of *fkh* and *fkh*, *dIAP1*-expressing RFP-positive clones compared to relevant control RFP-positive clones binned into two statistically significant clone size categories (*fkh* 1-6 cells and above 6 cells, *fkh*, *dIAP1* 1 cell clones and above 1 cell clones). Color code, genotype pairing and calculations are the same as in Fig. 6G and S6A. All graphs report mean and SEM. Significant differences between non-parametrically distributed data sets was tested by applying a one-tailed WMW test. A one-tailed Welch's t test was applied to normally distributed data sets with unequal variances. * = p-val < 0.01, ** = p-val < 0.001, ns= not significant. See Fig. S6D for detailed p-values. 10 discs were analyzed for wt, *fkh* and *fkh*, *dIAP1* genotypes each, 8 discs for *dIAP1* controls.

(D) Table listing p-values calculated to test for statistical significant difference in relative loss of RFP clones (data shown in Fig. 6G and Fig. S6C) per clone size bin as indicated. Significant differences between non-parametrically distributed data sets were tested by applying a one-tailed WMW test. A one-tailed Welch's t test was applied to normally distributed data sets with unequal variances. Red = p-val < 0.01.

(E) Table listing p-values calculated to test for statistical significant difference in relative apoptotic volume ratios between *fkh* and *fkh*, *dIAP1*-expressing clones (data shown in Fig. 6I) per clone size bin as indicated. Significant differences between data sets were tested by applying two-tailed WMW test. Red = p-val < 0.01.

(F, G) Tie-Dye imaginal discs 30 h after clone induction carrying neutral RFP-expressing clones (grey in F, red in F''') or *ci*, RFP-expressing clones (grey in G, red in G''') and neutral GFP-expressing clones (grey in F', G', green in F''', G'''). Discs were stained for Patched (Ptc) (grey in F'', G'', F''', G''') to visualize the A/P boundary. White boxes frame higher magnification insets for overlays shown in F''' and G'''. Note general loss of *ci*-expressing clones and persistence of larger clones in the posterior compartment.

(H, I) Boxplot (H) and mean relative area (I) occupied by neutral RFP-expressing or *ci*, RFP-expressing clones normalized to neutral GFP clone area in the anterior and posterior compartment. Absolute clone areas were quantified in 9 wt control and 11 *ci*-expressing Tie-Dye discs at 30 h after clone induction. Significance of differences between data sets was tested by applying a two-tailed WMW test. (H) * = p-val < 0.01, ns= not significant. (I) Red = p-val < 0.01. **(J)** Single basolateral xy-sections of a wing pouch containing *fkh*, *dIAP1*-expressing clones at 30 h after clone induction. Clones are marked by expression of GFP (J',

green in J'''). The disc was stained for phalloidin (J) and the apoptotic marker Dcp-1 (J'', red in J'''). White box frames higher magnification inset shown in J'''.

(K, L) Examples of Weka segmented masks (Fiji) used for quantitative analysis in Fig. 6 H, I derived from images in Fig. S1I and Fig. S6J. Apoptotic volume (red) was tracked for each clone volume (green) in 3D. A single xy-section is shown.

(M) Single basal xy-section of a wing pouch containing *fkh*, *dIAP1*-expressing cells (M', green in M''') at 30 h after clone induction. The disc was stained for phalloidin (M) and the apoptotic marker Dcp-1 (M'', red in M'''). Much of the apoptotic signal derives from wild type cells (examples highlighted by yellow arrowheads). Dotted lines indicate positions at which xz cross-sections shown in Fig. 6J, K were reconstructed.

(N) Single basal xy-sections of a wing pouch containing *fkh*-expressing cells (N', green in N''') at 30 h after clone induction. The disc was stained for phalloidin (N) and the apoptotic marker Dcp-1 (N'', red in N'''). Much of the apoptotic signal derives from wild type cells (examples highlighted by yellow arrowheads).

(O, P) Single xy-sections of a wing discs containing *ey*-expressing cells (O', P', and green in O''', P''') induced by a short 8 min heat-shock (O) and a long 25 min heat-shock (P), imaged 30 h after clone induction. The disc was stained for phalloidin (O, P) and the apoptotic marker Dcp-1 (O'', P'', red in O''', P'''). Whereas apoptosis is mainly observed in small *ey*-expressing clones (O), much of the apoptotic signal derives from wild type cells if surrounded by large domains of *ey*-expressing cells (P).

Table S1

Genotypes and experimental conditions

Detailed genotypes and experimental conditions (time-point of dissection, heat-shock length, egg collection/larval staging and use of spacer between coverslip and slide for microscopy) of data represented in individual figures

Figure	Genotype	Time points	Heat-shock	Egg-coll.	spacer
	Figure 1				
1 A	*hsflp* $^{[122]}$ */+; FRT42D ubi-eGFP / FRT42D Psc-Su(z)2* $^{[XL26]}$*; +/+*	54 h	40 min	no	no
1 C	*hsflp* $^{[122]}$ */+; FRT42D ubi-mRFP / FRT42D Psc-Su(z)2* $^{[XL26]}$*; +/+*	0 h	45 min	no	yes
1 D	*hsflp* $^{[122]}$ */+; FRT42D ubi-mRFP / FRT42D Psc-Su(z)2* $^{[XL26]}$*; +/+*	30 h	45 min	no	yes
1 E, F	*hsflp* $^{[122]}$ */+; FRT42D ubi-mRFP / FRT42D Psc-Su(z)2* $^{[XL26]}$*; +/+*	54 h	45 min	no	yes
1 G	*UAS-fkh-3xHA / hsflp* $^{[122]}$*; + / +; act > y* $^{[+]}$ *> GAL4, UAS-GFP/+*	0h	10 min	no	yes
1 H	*UAS-fkh-3xHA / hsflp* $^{[122]}$*; + / +; act > y* $^{[+]}$ *> GAL4, UAS-GFP/+*	30 h	10 min	yes	yes
1 I	*UAS-fkh-3xHA / hsflp* $^{[122]}$*; + / +; act > y* $^{[+]}$ *> GAL4, UAS-GFP/+*	54 h	10 min	yes	yes
1 J-M	*hsflp* $^{[122]}$ */ +; + / +; act > y* $^{[+]}$ *> GAL4, UAS GFP / UAS-ci.HA.wt*	54h	10 min	no	yes
1 N-Q	*hsflp* $^{[122]}$ */ +; + / UAS-hop* $^{[TumL]}$*; act > y* $^{[+]}$ *> GAL4, UAS GFP / +*	54 h	8 min	no	yes
	Figure 2				
2 A	*UAS-fkh-3xHA / hsflp* $^{[122]}$*; + / +; act > y* $^{[+]}$ *> GAL4, UAS-GFP/+*	30 h	25 min	yes	yes
2 B	*hsflp* $^{[122]}$ */ +; + / UAS-ey ; act > y* $^{[+]}$ *> GAL4, UAS GFP/ +*	30 h	25 min	yes	yes
2 D	*UAS-fkh-3xHA / hsflp* $^{[122]}$*; + / +; act > y* $^{[+]}$ *> GAL4, UAS-GFP/+*	30 h	10 min	yes	yes
2 G	*hsflp* $^{[122]}$ */ UAS-fkh-3xHA; + / sqh-GFP; Act5C.GAL4 (FRT.CD2), UAS-RFP / +*	30 h	10 min	no	yes
2 H	*UAS-fkh-3xHA / hsflp* $^{[122]}$*; + / +; act > y* $^{[+]}$ *> GAL4, UAS-GFP/+*	30 h	10 min	no	yes
2 I	*hsflp* $^{[122]}$ */ UAS-fkh-3xHA; + / zip>GFP; Act5C.GAL4 (FRT.CD2), UAS-RFP /+*	30 h	10 min	no	yes
2 J-K	*hsflp* $^{[122]}$ */ +; FRT42D ubi-mRFP / FRT42D Psc-Su(z)2* $^{[XL26]}$*, vkg>GFP; +/+*	54 h	40 min	no	yes
2 M, N	*hsflp* $^{[122]}$*/ +; + / +; act > y* $^{[+]}$ *> GAL4, UAS-GFP/ +*	54 h	10 min	yes	yes
2 L, O	*UAS-fkh-3xHA / hsflp* $^{[122]}$*; + / +; act > y* $^{[+]}$ *> GAL4, UAS-GFP/+*	54 h	10 min	yes	yes
	Figure 4				
3 F, G	*hsflp* $^{[122]}$ */ +; + / UAS-p35; Act5C.GAL4 (FRT.CD2), UAS-RFP / UAS-RhoV14*	54 h	10 min	no	no
3 H	*hsflp* $^{[122]}$ */ +; + / UAS-p35; Act5C.GAL4 (FRT.CD2), UAS-RFP / UAS-RhoV14*	54 h	10 min	no	yes
3 I-J	*hsflp* $^{[122]}$ */ + ; + / UAS-p35; act > y* $^{[+]}$ *> GAL4, UAS-GFP / UAS-RhoV14*	54 h	20 min	no	yes

	Figure 5				
4 F-J	UAS-fkh-3xHA / hsflp $^{[122]}$; + / +; act > y$^{[+]}$ > GAL4, UAS-GFP/+	30 h	10 min	yes	yes
	Figure 6				
5 A	hsflp$^{[122]}$/+; ubi <stop <GFP^{nls}, act5C <stop <lacZ^{nls}/+; Act5C <stop <GAL4, UAS-his2A::RFP/+	30h	7 min	24h	no
5 B	UAS-fkh-3xHA/ hsflp$^{[122]}$; ubi <stop <GFP^{nls}, act5C <stop <lacZ^{nls}/+; Act5C <stop <GAL4, UAS-his2A::RFP/+	30h	7 min	24h	no
5 C	hsflp$^{[122]}$/ Y; ubi <stop <GFP^{nls}, act5C <stop <lacZ^{nls}/Sp or +; Act5C <stop <GAL4, UAS-his2A::RFP/UAS-dIAP	30h	7 min	24h	no
5 D	UAS-fkh-3xHA/ hsflp$^{[122]}$; ubi <stop <GFP^{nls}, act5C <stop <lacZ^{nls}/Sp or +; Act5C <stop <GAL4, UAS-his2A::RFP/UAS-dIAP	30h	7 min	24h	no
5 J-K	UAS-fkh-3xHA / hsflp $^{[122]}$; + / +; act > y$^{[+]}$ > GAL4, UAS-GFP/UAS-dIAP	30 h	30 min	no	no
5 L,M,O	hsflp $^{[122]}$/ Y or +; UAS-Ras^{V12} / + ; act > y$^{[+]}$ > GAL4, UAS-GFP/+	30h	10 min	no	no
5 P,Q,R	hsflp $^{[122]}$/ Y or +; UAS-Ras^{V12} / + ; act > y$^{[+]}$ > GAL4, UAS-GFP/+	30h	27 min	no	no
5 N	hsflp $^{[122]}$/ Y or +; Sp / CyO ; act > y$^{[+]}$ > GAL4, UAS-GFP/+	30 h	10 min	no	no
	Figure S1				
S1 A-B	hsflp $^{[122]}$/+; FRT42D ubi-eGFP / FRT42D Psc-Su(z)2$^{[1b8]recA}$; +/+	27 h	60 min	no	no
S1 C	hsflp $^{[122]}$/+; FRT42D ubi-eGFP / FRT42D Psc-Su(z)2$^{[1b8]recA}$; +/+	72 h	60 min	no	no
S1 D-E	hsflp $^{[122]}$/+; FRT42D ubi-eGFP / FRT42D Psc-Su(z)2$^{[1b8]recA}$, yki$^{[B5]}$; +/+	27 h	60 min	no	no
S1 F	hsflp $^{[122]}$/+; FRT42D ubi-eGFP / FRT42D Psc-Su(z)2$^{[1b8]recA}$, yki$^{[B5]}$; +/+	72 h	60 min	no	no
S1 G	hsflp $^{[122]}$/+; FRT42D ubi-eGFP / FRT42D Psc-Su(z)2$^{[XL26]}$; +/+	54 h	40 min	no	no
S1 H	hep$^{[r75]}$/Y; FRT42D ubi-eGFP / FRT42D Psc-Su(z)2$^{[XL26]}$; T155 Gal4 UAS-flp/+	/	/	no	no
S1 I	UAS-fkh-3xHA / hsflp $^{[122]}$; + / +; act > y$^{[+]}$ > GAL4, UAS-GFP/+	30 h	7 min	no	no
S1 J-K	UAS-fkh-3xHA / hsflp $^{[122]}$; + / UAS-p35; act > y$^{[+]}$ > GAL4, UAS-GFP/ +	54 h	10 min	no	yes
S1 L	UAS-fkh-3xHA / hsflp $^{[122]}$; + / +; act > y$^{[+]}$ > GAL4, UAS-GFP/ UAS-dIAP	54 h	10 min	no	yes
	Figure S2				
S2 A	hsflp $^{[122]}$/ +; + / +; act > y$^{[+]}$ > GAL4, UAS-GFP/ +	30 h	10 min	yes	yes
S2 A`	hsflp $^{[122]}$/ +; + / +; act > y$^{[+]}$ > GAL4, UAS-GFP/ +	30 h	25 min	yes	yes
S2 B	UAS-fkh-3xHA / hsflp $^{[122]}$; + / +; act > y$^{[+]}$ > GAL4, UAS-GFP/+	30 h	10 min	yes	yes
S2 B`	UAS-fkh-3xHA / hsflp $^{[122]}$; + / +; act > y$^{[+]}$ > GAL4, UAS-GFP/+	30 h	25 min	yes	yes
S2 C	hsflp $^{[122]}$/ +; + / +; act > y$^{[+]}$ > GAL4, UAS-GFP/ +	54 h	10 min	yes	yes

S2 C`	*hsflp [122]/ +; + / +; act > y[+] > GAL4, UAS-GFP/ +*	54 h	25 min	yes	yes
S2 D, E	*UAS-fkh-3xHA / +, act > CD2 > GAL4, UAS GFP/ +; MKRS hsflp / +*	54 h	60 min	no	no
S2 D`, E`	*UAS-fkh-3xHA / hsflp [122]; + / +; act > y[+] > GAL4, UAS-GFP/+*	54 h	40 min	no	no
S2 H`	*act > CD2 > GAL4, UAS GFP/ UAS-AbdB; MKRS hsflp / +*	54 h	60 min	no	no
S2 F`	*hsflp [122] / +; + / UAS-AbdB ; act > y[+] > GAL4, UAS GFP/ +*	54 h	10 min	no	no
S2 G`, I`	*hsflp [122] / +; + / UAS-AbdB ; act > y[+] > GAL4, UAS GFP/ +*	54 h	40 min	no	no
S2 L`	*act > CD2 > GAL4, UAS GFP/ UAS-ey; MKRS hsflp / +*	54 h	60 min	no	no
S2 J`	*hsflp [122] / +; + / UAS-ey ; act > y[+] > GAL4, UAS GFP/ +*	54 h	10 min	no	no
S2 K`, M`	*hsflp [122] / +; + / UAS-ey ; act > y[+] > GAL4, UAS GFP/ +*	54 h	40 min	no	no
S2 N``	*hsflp [122] / +; + / + ; act > y[+] > GAL4, UAS GFP/ UAS-fln-HA3*	54 h	10 min	no	no
S2 N`	*hsflp [122] / +; +; act > y[+] > GAL4, UAS GFP/ UAS-dmyc*	54 h	10 min	no	yes
S2 P`-P```	*hsflp [122] / +; +/ +; act > y[+] > GAL4, UAS GFP / UAS-tkv.CA*	54 h	10 min	no	no
S2 Q`-Q```	*hsflp [122] / UAS-armS10; + / +; act > y[+] > GAL4, UAS GFP / +*	54 h	10 min	no	no
S2 R`-R```	*hsflp [122] / +; + / +; Act5C.GAL4 (FRT.CD2), UAS-RFP / UAS-vg*	54 h	10 min	no	no
S2 S`-S```	*hsflp [122] / +; + / +; act > y[+] > GAL4, UAS GFP / UAS-hth*	54 h	10 min	no	no
S2 U`-U``	*hsflp [122] / +; + / +; act > y[+] > GAL4, UAS GFP / UAS-ci.HA.wt*	54 h	30 min	no	yes
	Figure S3				
S4 A-B	*hsflp [122] / UAS-fkh-3xHA; + / sqh-GFP; Act5C.GAL4 (FRT.CD2), UAS-RFP / +*	30 h	10 min	no	yes
S4 C-D	*UAS-fkh-3xHA / hsflp [122]; + / +; act > y[+] > GAL4, UAS-GFP/+*	30 h	10 min	no	yes
S4 E-F	*hsflp [122] / UAS-fkh-3xHA; + / zip>GFP; Act5C.GAL4 (FRT.CD2), UAS-RFP /+*	30 h	10 min	no	yes
S4 G-H	*UAS-fkh-3xHA / hsflp [122]; + / +; act > y[+] > GAL4, UAS-GFP/+*	30 h	10 min	no	yes
S4 I-J	*hsflp [122] / UAS-fkh-3xHA; + / +; Act5C.GAL4 (FRT.CD2), UAS-RFP / sqh>MoeGFP*	30 h	15 min	no	yes
S4 K-M	*UAS-fkh-3xHA / hsflp [122]; +/ +; act > y[+] > GAL4, UAS-GFP/+*	30 h	10 min	no	yes
S4 N, P	*UAS-fkh-3xHA / hsflp [122]; + / +; act > y[+] > GAL4, UAS-GFP/+*	30 h	10 min	yes	yes
S4 O, Q	*UAS-fkh-3xHA / hsflp [122]; + / +; act > y[+] > GAL4, UAS-GFP/+*	30 h	15 min	yes	yes
	Figure S6				
S6 F	*hsflp[122]/+ or Y; ubi <stop <GFP^{nls}, act5C <stop <lacZ^{nls}/Sp; Act5C <stop <GAL4, UAS-his2A::RFP/Dr*	30 h	7 min	24 h	no
S6 G	*hsflp[122]/+ or Y; ubi <stop <GFP^{nls}, act5C <stop <lacZ^{nls}/Sp; Act5C <stop <GAL4, UAS-his2A::RFP/UAS-ci.HA.wt*	30 h	7 min	24 h	no

S6 J	*UAS-fkh-3xHA / hsflp* $^{[122]}$*; + / +; act > y*$^{[+]}$ *> GAL4, UAS-GFP/UAS-dIAP*	30 h	7 min	no	no
S6 K	*UAS-fkh-3xHA / hsflp* $^{[122]}$*; + / +; act > y*$^{[+]}$ *> GAL4, UAS-GFP/+*	30 h	7 min	no	no
S6 L	*UAS-fkh-3xHA / hsflp* $^{[122]}$*; + / +; act > y*$^{[+]}$ *> GAL4, UAS-GFP/UAS-dIAP*	30 h	7 min	no	no
S6 M	*UAS-fkh-3xHA / hsflp* $^{[122]}$*; + / +; act > y*$^{[+]}$ *> GAL4, UAS-GFP/UAS-dIAP*	30 h	30 min	no	no
S6 N	*UAS-fkh-3xHA / hsflp* $^{[122]}$*; + / +; act > y*$^{[+]}$ *> GAL4, UAS-GFP/+*	30 h	30 min	no	no
S6 O	*hsflp* $^{[122]}$ */ + or Y; + / UAS-ey; act > y*$^{[+]}$ *> GAL4, UAS-GFP/ +*	30h	7 min	no	no
S6 P	*hsflp* $^{[122]}$ */ + or Y; + / UAS-ey; act > y*$^{[+]}$ *> GAL4, UAS-GFP/ +*	30h	30 min	no	no
	Movie S1				
	hsflp $^{[122]}$ */+; FRT42D ubi-mRFP / FRT42D Psc-Su(z)2*$^{[XL26]}$*; +/+*	102 h	45 min	no	yes

Supplemental experimental procedures

Drosophila genetics

For detailed fly genotypes and heat-shock induction conditions please refer to Table S1. Briefly, all crosses were kept on standard media at 25°C. FLP/FRT and 'GAL4/UAS flip-out' overexpression experiments utilized heat-shock-driven expression of a flipase. The respective crosses were allowed to lay eggs for 72 h at 25°C followed by a heat-shock at 37°C for 40-60 min (FLP/FRT) or 5-25 min ('GAL4/UAS flip-out'). Larvae were dissected at wandering 3rd instar stage or as indicated (0 h, 30 h, 54 h or 102 h after heat-shock). To generate tightly staged larval populations for clone shape and actin intensity quantification experiments (Fig. 2 & 5), crosses were allowed to lay eggs for 7 h on grape plates. 1st instar larvae were transferred to standard media 24 h later (50 larvae per vial) and heat-shocked 72 h AEL at 37°C. For Tie-Dye experiments (Fig. 6), crosses were allowed to lay eggs on standard media for 24 h at 18°C to prevent early induction of flipase expression. 34 h after start of egg lay, vials were transferred to 25°C and 72 h after egg lay, larvae were subjected to a heat-shock at 37°C for 7 min. Larvae were dissected 30 h after heat-shock and analyzed as described below.

Immunohistochemistry and imaging

Imaginal discs were dissected and fixed in 4% formaldehyde/PBS for 18 min at room temperature. Washes and blocking were performed in PBS + 0.1 Triton X-100 (PBT) and PBT+5% normal goat serum, respectively. Discs were incubated with primary antibodies overnight at 4°C: guinea pig anti-Spaghetti-squash 1P (1:400, gift from Robert Ward), mouse anti-βPS-Integrin (1:10, DSHB, CF.6G11), rabbit anti-phospho-Ezrin/Radixin/Moesin (1:500, Cell Signaling, #3149), mouse-anti Rho1 (1:100, DSHB, p1D9), mouse anti-wingless (1:100, DSHB, 4D4), mouse anti-patched (1:20, DSHB, Apa1), rabbit anti-Cleaved Drosophila Dcp-1 (1:250) (Cell Signaling, #9578). Discs were counterstained with DAPI (0.25 ng/μl, Sigma), Phalloidin (Alexa Fluor 488 and Alexa Fluor 647 phalloidin,1:100, Molecular Probes, or Phalloidin-TRITC, 1:400, Sigma). Secondary antibodies (coupled to Alexa Fluorophores, Molecular Probes) were incubated for 2 h at room temperature. Discs were mounted using Molecular Probes Antifade Reagents (#S2828). To prevent squeezing of samples by coverslips, two stripes of double-sided tape (Tesa, #05338) were placed on the slide. Samples were imaged using a Leica TCS SP5 confocal microscope equipped with HCX PL APO Lambda Blue 20x (NA 0.7) and HCX PL APO Lambda Blue 63x (NA 1.4) lenses. Images were processed and analyzed using Fiji (ImageJ 1.48b).

Collagenase treatment

Wing imaginal discs were incubated for 12 min in 200 μl - 400 μl of Collagenase (CLSPA; Worthington Biochemical Corp, 1000 u/ml + 2 mM $CaCl_2$ and 2mM $MgCl_2$). Discs were immediately fixed for 18 min in 4% PFA/PBS and processed as described above.

Image Analysis and Quantification using Fiji

General information on imaging conditions

For all clone shape quantifications at least 3 imaginal discs were analyzed. To obtain an overview of the pouch, a stack was imaged using a 63x objective (zoom 1, xy 0.24 μm, z 1.51 - 2.98 μm). Subsequently, selected regions were imaged at higher magnification (zoom 3, xy 0.08 μm, zoom 2.5 xy 0.096 μm, z 0.42 - 1.0 μm). Channels were merged in FIJI and cropped stacks representing individual clones were used for nuclei counting, clone volume and circularity measurements. For Tie-Dye experiments, image stacks of the pouch and hinge region (20x objective, xy 0.45 - 0.54 μm, z 2.01 - 2.18 μm) were generated and analyzed. For Dcp-1 quantifications, image stack using a 63x objective (zoom 1, xy 0.24 μm, z 2.01 - 3.48 μm) were analyzed.

General information on statistical analysis of imaging data

Every data set was checked for normality of distribution and homogeneity of variances by applying Shapiro's and Bartlett's test, respectively. The α value for each analysis was set to 0.01 ($\alpha = 0.01$). Wilcoxon Signed-Rank test (WSR), Wilcoxon-Mann-Whitney test (WMW) and Welch's t-test were applied as indicated in figure legends.

Quantification of actin intensities (Fig. 2)

Actin intensities were measured on reconstructed xz-cross-section (reslice tool, 1 μm spacing, top and left reslice) from zoom 1 image stacks. In overlay stacks of phalloidin and clonal marker, the section at a vertical position through the center of radially symmetric clones was identified. Apical junctions and lateral surfaces were selected and average fluorescence intensity measured using the 'measure' tool. For each clone, the two lateral MWI interfaces, up to 8 cell surfaces inside the clone and up to 15 cell surfaces outside the clone, near the MWI, were measured. Data points were averaged to obtain 1 value for wt/wt, wt/mis and mis/mis interfaces for each clone. These values were then normalized to the average mean intensity of actin staining on wt/wt interfaces within an experimental series.

Quantification of clone volumes (Fig. 5)

To determine volumes of individual clones, the GFP-signal defining the clone volume was used to generate a quantification mask employing the 'Threshold' (settings: default, stack histogram, dark background, between 5-30) and 'Remove outlier' (settings: black and white pixel removal with radii 0.5-2 at a threshold of 50) function of Fiji. The resulting binary mask was used to measure clone area in each section using the 'Analyze particles' function (settings: size (micron2): usually 10-Infinity for zoom 3 images and 5-Infinity for zoom 1 images, Circularity 0.00-1.00). To control if the clone area was well defined, the stack displaying the outlines of measured particles was merged with the original GFP stack and manually validated. Particle area measurements in all sections for each clone were summed up and multiplied with z-stack step size to obtain clone volumes.

Quantification of cell number and average cell volumes (Fig. 5)

Nuclei counts were obtained using reslices of zoom 3 image stacks. In an overlay stack between DAPI and clonal marker, nuclei were tracked through each slice and counted with the help of the text tool of Fiji. An average cell volume for each experimental condition was calculated by dividing the clone volume obtained above by nuclear counts for each individual clone. Average cell volumes obtained in zoom 3 images were used to extrapolate cell numbers from the GFP-volume measured for additional clones imaged at zoom 1 for the same experimental conditions.

Circularity measurements (Fig. 3)

To calculate circularity of *fkh*-overexpressing and neutral wild type clones, we used image stacks of discs that were heat-shocked for 10min and analyzed 30 h and 54 h later, as well as disc heat-shock for 25 min and analyzed 30 h after clone induction. A section at exactly a third (for *fkh*-expressing clones) and a fifth (for *Rho*^{V14}-expressing clones) of the disc height from the most basal section was identified and used for tracing a clone outline with the help of the polygon tool in Fiji. Area and perimeter of this region were measured and circularity was defined using the following equation:

$$C = 4\pi \frac{\text{Area}}{\text{Perimeter}^2}. \quad (1)$$

Quantification of wild type cell volumes

To quantify cell volumes and aspect ratios of wild type cells we analyzed stage-matched wild type imaginal discs derived from a neutral control cross or wild type cells next to *fkh*-overexpressing cells. Based on a phalloidin counterstain, 2 regions within the same stack were chosen for analysis: 15-25 wild type cells with (1) small apical cell areas and (2) big apical areas. This approach was chosen to reflect the endogenous diversity of cell shapes in imaginal discs. The height of cells was defined in a reslice and multiplied by the apical area of all selected cells. An average wild type cell volume was obtained by dividing the resulting volume through the number of selected cells.

Experimental clone shape analysis (Fig. 5)

To quantify experimental clone shapes and to compare them to computational simulations, we extracted characteristic shape coordinates for each clone. Precision of coordinate selection was verified between zoom 3 and zoom 1 image stacks. Zoom 1 image data was then used for further analysis because more clones could be sampled. Coordinates were defined using the Fiji Point picker tool. From each coordinate set, 4 characteristic quantities were extracted using MATLAB. First the apical surface of the wild type tissue was identified by the least-square fit of a linear function through all apical wild type points. Tissue height h was defined as the average distance of all basal wild type points to the apical surface. The absolute apical indentation u_a for each clone was defined as distance of the apical midpoint of the cyst from the apical wild type surface. Analogously, the basal deformation u_b was defined as the distance of the basal clone midpoint to a straight line fitted through basal wild type points. The apical and the basal width w_a and w_b are defined as the distance between the left and right clone interfaces apically and basally, respectively. As final step the measurements were normalized with the height h of the surrounding tissue, to obtain relative tissue deformations. For each clone, these normalised quantities were obtained in two perpendicular cross

sections and then averaged, to account for small deviations from a rotationally symmetric clone shape. These relative deformations u_a/h, u_b/h, w_a/h and w_b/h are plotted as a function of the clone size in Fig. 4 for the optimal parameter set and in Fig. S5 for variations around this set.

Tie-Dye clone size frequency - *fkh* and *fkh*, *dIAP* (Fig. 6)

Imaginal disc of all Tie-Dye genotypes were counterstained with DAPI and phalloidin and were processed in Fiji as follows: Peripodial membrane clones were excluded from the analysis and deleted in both GFP and RFP channels manually using the polygon and clear function. Since a majority of the Tie-Dye signal in the notum derives from adepithelial cells, the ROI set for subsequent analysis only considered the pouch and hinge regions of the disc. To identify the optimal fluorescence threshold for RFP and GFP masks, different thresholds were tested. This was done by choosing a threshold, removing outliers and generating outlines of the resulting mask. Mask outlines were merged with original images and checked for visual match with GFP and RFP borders. GFP and RFP thresholds were determined separately for each disc, but were validated by optimal fit and similar volume measurements for clones that express both GFP and RFP.
We decided to exclude GFP/RFP double-labeled clones from our final analysis because small differences in GFP and RFP masks would have caused us to systematically underestimate their size. We therefore only selected GFP or RFP positive clones by subtracting the mask of GFP from that of RFP and vice versa using the image calculator tool. In the resulting masks (GFP only, RFP only) outliers were removed by a series of 'Remove outlier' commands (settings: black and white pixel removal with radii 0.5-3 at a threshold of 50). Mask holes were filled using the 'Fill holes' tool.
The resulting GFP only and RFP only masks were analyzed using the 3D object counter (Version V2.0, Threshold 128, No exclusion of size or edges, Maps to show: Objects, Results table to show: Summary, Statistics). Volume measurements were transferred to Excel. 3-4 small clones per disc where selected from the object map, identified in original image and nuclei were counted for each clone. The clone volume as determined by the 3D analysis was divided by the number of nuclei to obtain an average nuclei volume for each genotype.
GFP and RFP volumes below an empirically determined minimum cell volume for each genotype were excluded from the analysis. All remaining data points were binned into multiples of empirically determined average cell volumes for each genotype. Relative loss of RFP clones was calculated for each chosen bin: (RFP clone count - GFP clone count) / GFP clone count within the analyzed bin.

Clone size quantification in *ci*-expressing Tie-Dye discs (Fig. S6)

ci-expressing and control Tie-Dye wing imaginal discs were counterstained with DAPI and Patched (Ptc) to mark the anterior/posterior compartment boundary. Image stacks were processed and analyzed in Fiji as follows: ROIs for the total pouch, the anterior and posterior compartment were generated on maximum projections of the Ptc staining. Maximum projections of GFP and RFP image substacks that included all clones in the pouch were created and segmentation masks using the threshold function followed by a series of 'Remove outlier' commands were established. GFP positive clones were selected by subtracting the RFP mask from the GFP mask using the image calculator tool. GFP and RFP clone areas in the whole pouch, the posterior and the anterior compartment were measured using the 'Analyze particle' function within previously defined ROIs.

Dcp-1 volume quantification (Fig. 6)

Imaginal disc carrying regular GAL4/UAS flip-out clones expressing *fkh* or *fkh*, *dIAP* were stained for Dcp-1, DAPI and phalloidin. Images were processed in Fiji as follows: Peripodial membrane clones were excluded from the analysis and manually deleted (GFP channel) using the polygon and clear function. Each image plane was classified in background, clone and Dcp-1 signal using the trainable Weka segmentation tool and segmentation holes were filled using the 'Fill Hole' function. The quality of the segmentation was verified visually. Dcp-1 signal that was not part of a flip-out clone was removed by combining Dcp-1 and GFP masks with a logical AND operation. Segmented image planes were used to reconstruct 3D objects of clonal and Dcp-1 stainings. For each clone, the total volume, the volume of all included Dcp-1 sites was determined. All 3D operations were performed with custom macros using the API of the 3D ImageJ Suite. For defining the average cell volume, 3-4 clones were selected per disc, nuclei within the clones were counted and divided by clone volume. GFP volumes below an empirically determined minimum cell volume for each genotype were excluded from the analysis. All remaining data points were binned into multiples of empirically determined average cell volumes for each genotype.

Modeling procedures

A 3D Vertex Model for simple epithelia

In this section we introduce and motivate a 3D Vertex Model for epithelial mechanics, which is then applied to quantitatively understand the formation of cysts in the *Drosophila* imaginal wing disc as a function of the distribution of forces generated inside the disc. Our 3D Vertex Model can be seen as a generalisation of the 2D vertex model [S3], where both apical and basal cell surfaces are taken into account, and the epithelium can deform in 3 dimensions.
We start by describing in detail how the epithelium is represented by a set of vertices, after which we show how internal and external forces are included in the vertex model description. In the last parts of the introduction we describe how topological transitions are implemented in our 3D Vertex Model and we provide details on how the simulations were carried out.

Geometry of the 3D Vertex Model

In the 3D Vertex Model the tissue is represented by a set of vertices. We distinguish a set of apical and basal vertices with positions $\mathbf{x}_a$ and $\mathbf{x}_b$, associated with the epithelium's apical and basal surfaces. Vertices are connected by a network of apical, basal and lateral bonds which represent the cell outlines. The apical and basal vertices and bond networks are taken to have the same topology, but can have different positions (Fig.3). Lateral bonds therefore simply connect each apical vertex to its basal counterpart.
Each cell α is represented by a set of M apical and basal vertices $\mathbf{x}_a^{\alpha,i}$ and $\mathbf{x}_b^{\alpha,i} (i = 1...M)$ and their associated bonds. Because vertices constituting a surface are not necessarily coplanar, the surface enclosing the cell has to be defined. Each interface k is defined by a subset of vertices $\mathbf{x}_1^k, \mathbf{x}_2^k, ..., \mathbf{x}_N^k$. The contour line C of the surface is then given by the path ($\mathbf{x}_1^k - \mathbf{x}_2^k - ... - \mathbf{x}_N^k - \mathbf{x}_1^k$) and the associated centre of mass $\mathbf{c}^k$ is defined as:

$$\mathbf{c}^k = \frac{1}{L}\int_C \mathbf{r}(s) ds \tag{2}$$

$$= \frac{1}{\sum_i |\mathbf{x}_i^k - \mathbf{x}_{i+1}^k|} \sum_i |\mathbf{x}_i^k - \mathbf{x}_{i+1}^k| \frac{\mathbf{x}_i^k + \mathbf{x}_{i+1}^k}{2}, \tag{3}$$

where $\mathbf{r}(s)$ is a vector pointing on the contour C, s is an Euclidean coordinate going along the contour C, and the sum over vertices i, j is performed on vertices belonging to the interface k. We then use the center of mass point $\mathbf{c}^k$ to generate a triangulation of the interface k, by joining the center of mass $\mathbf{c}^k$ with all vertices belonging to the interface. Each cell is therefore enclosed by a set of planar triangles (Fig.3C) and the triangulation is used to compute cell volume as well as the apical, basal and lateral surface areas.

Mechanical forces in the 3D Vertex Model

In the vertex model forces act to displace vertices that describe the epithelium. These forces are derived from a virtual work, based on the tensions generated inside the epithelium and external mechanical constraints. To emphasise the different contributions, the virtual work δW is written as the sum of the work exerted within

the epithelium δW_i and outside the epithelium δW_e:

$$\delta W = \delta W_i + \delta W_e. \tag{4}$$

The details of these contributions are given in the following sections.

Internal forces

The internal virtual work is written

$$\delta W_i = \sum_k T_k \delta A_k + \sum_{i,j} \Lambda_{ij} \delta l_{ij} - \sum_\alpha P_\alpha \delta V_\alpha, \tag{5}$$

where the indices i, j label vertices, the index k labels interfaces, and the index α labels cells. The volume of cell α, the surface area of interface k and the length of the bond joining the vertices i and j are denoted V_α, A_k, and l_{ij} respectively. The pressure acting in cell α is denoted P_α, the surface tension on interface k, T_k, and the line tension acting on the bond $\langle i, j \rangle$, Λ_{ij}. Surface tensions T_k and line tensions Λ_{ij} are thought to arise from the actomyosin cortical cytoskeleton. The contractility of myosin molecular motors in the surface actin network leads to the establishment of a surface tension [S4]. In general the tensions generated in surfaces and along lines can depend on the area of the surface or the length of the actin cable (i.e. $T_k = T_k(A_k)$, $\Lambda_{ij} = \Lambda_{ij}(l_{ij})$). For cyst simulations, we assume that all surfaces have a constant tension if their area is larger than a preset threshold, but if further constricted they exhibit a linearly elastic behaviour towards a preferred area value:

$$T_k(A_k) = \begin{cases} T_k^0 & \text{for } A_k > A_k^0 \\ T_k^* + \frac{A_k}{A_k^0}(T_k^0 - T_k^*) & \text{for } 0 \leq A_k < A_k^0. \end{cases} \tag{6}$$

This choice is motivated by the experimental observation that apical surfaces vanish rarely completely during cyst formation. Only apical cell surfaces were assumed to experience this elasticity and for them we chose in simulations $A_k^0 = \bar{A}/2$ and $T_k^* = -T_k^0$, with $\bar{A}$ being the average apical surface area of wildtype cells. Line tensions are assumed to be constant and independent of the length of the bond.
Cells are assumed to be able to maintain a preferred volume. The cell pressure P_α is taken to be linear in the deviation of the volume from the preferred volume

$$P_\alpha = -K_\alpha (V_\alpha - V_\alpha^0), \tag{7}$$

where V_α^0 is the preferred cell volume of cell α and the proportionality constant K_α is its bulk elastic modulus. In this study, K_α is chosen large enough that cells are close to incompressibility, with a relative deviation of the cells' volume from their preferred volume smaller than 0.001.

External forces acting on the epithelium

We take into account two additional forces: an external in-plane surface tension constraining the area of the tissue, and a term representing tissue attachment to the extracellular matrix (ECM). The ECM is considered

to be a flat surface located at $z = 0$. The external virtual work then reads

$$\delta W_e = \sum_v k_v x_v \delta x_v - T_{\text{ext}} \delta A^{tot}, \tag{8}$$

where the distance of a vertex v to the closest ECM point is denoted x_v, and the total tissue surface area is denoted A^{tot}. The spring modulus k_v represents elastic bonds attaching the tissue to the extracellular matrix, and it is non-zero only for basal vertices as the apical tissue surface is not connected to the ECM. In the wing disc, experimental evidence indicates that the tissue is under compression, $T_{\text{ext}} < 0$. The compression forces possibly arise from global constraints imposed by the ECM, which surrounds the wing disc. Indeed, removal of the ECM leads to area expansion of the tissue (Fig.S4D-G).

Forces acting on vertices

The force $\mathbf{F}_i$ that acts on vertex $\mathbf{x}_i$ can then be obtained by taking the derivative of the virtual work with respect to the vertex position (Fig. 3A):

$$\mathbf{F}_i = -\frac{\partial W}{\partial \mathbf{x}_i}. \tag{9}$$

Note that the centers of mass of the surfaces are not taken as degrees of freedom (cf. Eq. 3), but have to be considered in the calculation of the forces acting on the single vertices. To clarify this, we note that the virtual work introduced in Eq. 4 is in general a function of the positions of the vertices and centers of mass, $\delta\bar{W}(\mathbf{x}_i, \mathbf{c}_i)$. As the centers of mass depend on vertices positions through Eq. 3, the virtual work in Eq. 9 can be obtained from

$$\delta W(\mathbf{x}_i) = \delta\bar{W}(\mathbf{x}_i, \mathbf{c}_k(\mathbf{x}_i)), \tag{10}$$

such that the force on any vertex i can be obtained as follows:

$$\mathbf{F}_i = -\frac{\partial\bar{W}}{\partial \mathbf{x}_i} - \sum_{k=1}^{M} \frac{\partial\bar{W}}{\partial \mathbf{c}^k}\frac{\partial \mathbf{c}^k}{\partial \mathbf{x}_i}. \tag{11}$$

The tissue is in equilibrium if the forces on all vertices i vanish:

$$\mathbf{F}_i = \mathbf{0}. \tag{12}$$

Topological transitions

In addition to changes in the position of vertices the tissue can undergo T_1 topological transitions, in the course of which cells exchange neighbours. Transitions occur through the shrinkage of the interface in between two cells and the expansion of a new interface between two cells which were previously not in contact.

We impose here that T_1 topological transitions must occur both on the apical and the basal side, such that both networks maintain the same topology. In the implementation of the 3D Vertex Model, a T_1 transition occurs if the apical and the basal length of an edge e fall under the threshold length l^{T1} and the forces acting on the vertices of the edge tend to further reduce the apical and basal length of the edge (Fig. S4A). In the exemplary illustration the edge e, common to cells 2 and 4, is then replaced by a single fourfold vertex. This

new vertex can be replaced by a new bond, connecting the cells 2 and 4 which were previously unconnected. To decide if this topological transition is performed, a hypothetic new bond $e^{1,3}$ is introduced between the two cells 1 and 3, with the direction of the apical/basal edge obtained by connecting the apical/basal cell centres of the other two cells. The apical and basal cell centres of cell α are denoted $\mathbf{M}_a^\alpha$ and $\mathbf{M}_b^\alpha$ respectively. We denote $\mathbf{F}_a^2$ and $\mathbf{F}_a^4$ the forces acting on the apical vertices of the hypothetic edge e which are part of cell 2 and 4, and $\mathbf{F}_b^2$ and $\mathbf{F}_b^4$ the respective forces on the basal vertices. The separating forces for the hypothetic edge $e^{1,3}$ are given by the projection of the force on the opening direction:

$$f_a^{1,3} = (\mathbf{F}_a^2 - \mathbf{F}_a^4) \cdot \frac{\mathbf{M}_a^2 - \mathbf{M}_a^4}{|\mathbf{M}_a^2 - \mathbf{M}_a^4|} \tag{13}$$

$$f_b^{1,3} = (\mathbf{F}_b^2 - \mathbf{F}_b^4) \cdot \frac{\mathbf{M}_a^2 - \mathbf{M}_a^4}{|\mathbf{M}_a^2 - \mathbf{M}_a^4|} \tag{14}$$

$$f^{1,3} = f_a^{1,3} + f_b^{1,3}. \tag{15}$$

The hypothetic edge $e^{1,3}$ is allowed to open if both apical and basal opening forces are positive $f_a^{1,3} > 0, f_b^{1,3} > 0$. The newly established edge is assigned the initial apical and basal lengths l_a^{new} and l_b^{new}. The topology remains unchanged and the fourfold vertex is conserved if the formation of the new edge is unfavourable.

3D Vertex Model implementation for the numerical study of cyst formation

Periodic boundary conditions

In the simulation of the wing disc in the 3D Vertex Model, the epithelium is assumed to have periodic boundary conditions in x and y directions. A rectangular box with sizes L_x and L_y is therefore used for simulations (Fig.S4B). The size of the box is not fixed, and the positions of all vertices in the box are affinely rescaled when the lengths L_x and L_y are modified: under a change of tissue size $L_x \to L_x + \delta L_x, L_y \to L_y + \delta L_y$ every position $\mathbf{P}$ changes according to

$$\mathbf{P} \to \begin{pmatrix} \frac{L_x+\delta L_x}{L_x} & 0 & 0 \\ 0 & \frac{L_y+\delta L_y}{L_y} & 0 \\ 0 & 0 & 1 \end{pmatrix} \mathbf{P}. \tag{16}$$

The equilibrium system size (Fig.S4C) is obtained through the force balance equations:

$$\frac{\partial W}{\partial L_x} = \frac{\partial W_i(L_x, L_y)}{\partial L_x} - T_{\text{ext}} L_y = 0 \tag{17}$$

$$\frac{\partial W}{\partial L_y} = \frac{\partial W_i(L_x, L_y)}{\partial L_y} - T_{\text{ext}} L_x = 0. \tag{18}$$

Starting configurations

Simulations were performed with a tissue containing 2000 cells. To obtain different starting configurations of the tissue, random tissues were created by performing a 2D Voronoi tessellation of randomly distributed points in a periodic box of size $L_x^0 \times L_y^0$. Apical and basal vertices were then assigned the same x and y coordinates, but different z coordinates (Fig.S4B). This random initial configuration was then relaxed to an equilibrium shape by minimising the work function with respect to system size, vertex positions and topology

(Fig.S4C). For each set of examined mechanical parameters and each cyst size, we ran simulations with 15 different starting configurations of the tissue.

Minimisation algorithm

For the choice of the virtual work function we use for cyst simulations, δW can be integrated to yield

$$W = \sum_{\alpha} -P_\alpha V_\alpha + \sum_k W_k(A_k) + \sum_{ij} \Lambda_{ij} l_{ij} + \sum_v \frac{k_v}{2} d_v^2 - T_{\text{ext}} A^{tot}, \tag{19}$$

where $W_k(A_k) = \int_0^{A_k} T_k(\tilde{A}_k) d\tilde{A}_k$. For a fixed topology of the network of bonds and vertices, the work W can be minimised with respect to the position of the vertices and the system size L_x, L_y to find mechanical equilibrium configurations of the network. This is numerically done in a C++ implementation of the Polak-Ribiére conjugate gradient algorithm [S5].

Clone specification

An initial clone consisting of N mutant cells is created by assigning mutant properties to the N cells closest to an arbitrarily selected point in the relaxed homogeneous tissue (Fig.4A). To account for interface contraction, changes are applied to apical line and/or lateral surface tension at the interfaces between the two different cell populations, whereas all other mechanical properties of the mutant cells remain unchanged (Fig.4D-E). To account for increased bulk contraction in simulations, the lateral interface tension between mutant cells has been changed in comparison to the lateral surface tension between wild type cells, with the interface tension between mutant and wild type cells being the arithmetic mean of the two (Fig.4B-C).

Parameter search

In this section, we describe the parameter search procedure that was performed to reproduce the experimental dependency of four clone geometrical measurements on the number of cells in the clone N_c (Fig. 5C-D). Simulations involve 10 independent parameters listed in Table 1. Renormalising all lengths with the reference length scale $l_0 = (V_0)^{1/3}$ and line and surface tensions by the surface tensions T_l leaves 8 remaining unknown parameters.
To account for the shape of the wild type tissue, two additional constraints were introduced that we now describe. Eq. 67 is then used to obtain an relation between the average tissue aspect ratio $\beta = \langle h/\sqrt{A} \rangle$ and the external tension acting on the tissue T_{ext}:

$$\beta \simeq \frac{\gamma_a(\beta) + \gamma_b(\beta) - T_{\text{ext}}}{\frac{3^{1/4}}{\sqrt{2}} T_l}. \tag{20}$$

γ_a and γ_b denote the total tensions acting in the apical and basal surfaces of the tissue, and the corresponding expressions are given in Equations 68 and 69. Note that Eq. 67 was obtained for a regular hexagonal packing, and we assume it holds approximately true for the disordered packing considered here.
The average wild type aspect ratio of the pouch in the presence of the ECM was found to be $\beta^{wt} \simeq 16.2$ (Fig.S4F). The ECM possibly exerts an external tension T_{ext} on the tissue, and to estimate this tension the relaxation of the tissue after the removal of the ECM was analysed (Fig.S4D-G). Treatment of the wing discs

with collagenase led to significant ECM removal (Fig.S4D-E) and to the subsequent relaxation of the tissue shape on timescales of minutes, in the course of which the pouch flattened and the cells assumed a new aspect ratio $\beta^{coll} \simeq 11.6$ (Fig.S4F). Assuming that collagenase application only results in removal of mechanical constraints exerted by the ECM, this observation indicates the epithelium is under compression. Denoting T_{ext}^{wt} the compression due to the ECM, and T_{ext}^{coll} the remaining compression after collagenase treatment and removal of the ECM, the following two constraints enforce the tissue to have the right aspect ratio in both cases:

$$\beta^{wt} = \frac{\gamma_a(\beta^{wt}) + \gamma_b(\beta^{wt}) - T_{\text{ext}}^{wt}}{\frac{3^{1/4}}{\sqrt{2}} T_l} \tag{21}$$

$$\beta^{coll} = \frac{\gamma_a(\beta^{coll}) + \gamma_b(\beta^{coll}) - T_{\text{ext}}^{coll}}{\frac{3^{1/4}}{\sqrt{2}} T_l}. \tag{22}$$

Replacing the experimental values of β^{wt} and β^{coll} in equalities 21 and 22 imposes two constraints on the model parameters. Under these additional two constraints, a parameter search was performed to adjust the 8 last parameters listed in Table 1. We verified that the apical line tension and lateral surface tension around the clone must both be increased to explain experimental observations (Fig. S5C-G), we therefore assume here for simplicity that they are increased by the same factor. For each set of tested parameters 450 simulations with 30 different cyst sizes and 15 different initial tissue configurations were run, from which averages and standard error of the mean of the resulting cyst deformations were obtained, as shown in Fig. 5C-D. The corresponding curves of cyst deformation as a function of the number of cells within the cyst were generated for $\sim$200 different parameter sets. The set of parameters given in Table 1 was found to give rise to equilibrium shapes that closely resemble the experimental data. In order to identify the key mechanical parameters controlling the deformations, simulations were run where only single parameters were changed in comparison to the preferred set of parameters given in Table 1. The results are shown and described in Supplementary Figure S5.

**description**	**parameter**	**value**
cell volume	V_0/l_0^3	1
lateral surface tension	T_l/T_l	1.0
lateral surface tension around clone	T_l^c/T_l	3.0
apical surface tension	T_a/T_l	3.1
basal surface tension	T_b/T_l	6.95
external compression	T_{ext}/T_l	-4.2
apical line tension	$\Lambda_a/(l_0 T_l)$	0.18
apical line tension around clone	$\Lambda_a^c/(l_0 T_l)$	0.53
basal line tension	$\Lambda_b/(l_0 T_l)$	0.18
stiffness of ECM attachment	k_v/T_l	5.0

Table 1: List of parameters obtained from comparison of the vertex model to clone shape measurements as a function of clone size.

Description of cyst formation with a continuum theory

In this section an alternative modeling approach is introduced where the epithelium is represented by a thin layer, coupled to a solid substrate by elastic links. The tissue mechanical properties are accounted for by a Helfrich mechanical free energy based on the curvature and applied tension ([S6, S7]). An additional term represents the elastic coupling of the tissue to the flat ECM. We use this continuum description to derive conditions for the stability of a flat, homogeneous tissue. We also study the buckling threshold of a clone subjected to compression due to an additional tension acting along its boundary. A similar question of budding of a domain under line tension has been discussed for lipid membranes in [S8]. For a classical introduction into the buckling of circular plates under a uniform load see for instance [S9, S10].

Parametrisation and the free energy

The tissue is described here as a thin 2D layer, where a point on the mid-surface representing the tissue $\mathbf{X}$ is parametrised in the Monge gauge (Fig.S4H)

$$\mathbf{X} = h(x, y)\mathbf{e}_z. \tag{23}$$

The tissue has a bending rigidity κ which penalizes deviation of the local curvature C from the preferred curvature C_0. In addition, an elastic material with elastic modulus k is assumed to connect the tissue to the underlying ECM, represented as a flat surface at position $h = 0$ (Fig.S4H). The corresponding mechanical work for a region of tissue S then reads

$$W = \int_S dA \left(\frac{\kappa}{2} C^2 - \kappa C_0 C + w_0(\rho) + \frac{k}{2} h^2 \right), \tag{24}$$

where $C = \frac{1}{R_1} + \frac{1}{R_2}$ denotes the mean curvature with R_1 and R_2 the two principal radii, ρ is the cell density, and w_0 is the mechanical energy density at zero curvature and zero height. In the limit of weak bending $|\nabla h| \ll 1$, the work function reads at second order in height deviation

$$W \approx W_0 + \int dA \left(\gamma \frac{(\nabla h)^2}{2} + \frac{\kappa}{2} (\Delta h)^2 - \kappa C_0 \Delta h + \frac{k}{2} h^2 \right) \tag{25}$$

where we have introduced the surface tension $\gamma = d(\rho^{-1} w_0)/d(\rho^{-1})$.

Linear stability of the flat, homogeneous tissue

The tissue mid plane position is described here in the 2D Monge gauge by the height function $h = h(t, \mathbf{x})$ where $\mathbf{x} = (x, y)$ describes the position in the plane. The tissue is defined on a disc centred at the origin with radius R, $\Omega(R) = \{\mathbf{x} : |\mathbf{x}| \leq R\}$. Introducing an effective friction coefficient $\alpha > 0$, the dynamical equation for the tissue height reads by differentiating Eq. 25 with respect to h:

$$\alpha \partial_t h(\mathbf{x}, t) = -\kappa \Delta^2 h(\mathbf{x}, t) + \gamma \Delta h - k h(\mathbf{x}, t) \tag{26}$$

with $\Delta = \partial_x^2 + \partial_y^2$ the Laplacian operator. If the tissue is periodic on the region $\Omega = [0, L] \times [0, L]$, the Fourier transform of $h(\mathbf{x}, t)$ on Ω is defined as:

$$\tilde{h}(\mathbf{q}, t) = \int_{\Omega} h(\mathbf{x}, t) e^{i\langle \mathbf{q}, \mathbf{x} \rangle} d\mathbf{x} \tag{27}$$

for $\mathbf{q} \in \tilde{\Omega} = [1/L, \infty] \times [1/L, \infty]$. Denoting $q = |\mathbf{q}|$, the dynamic equation (26) can be written in Fourier space:

$$\alpha \partial_t \tilde{h}(\mathbf{q}, t) = (-\kappa q^4 - \gamma q^2 - k) \tilde{h}(\mathbf{q}, t), \tag{28}$$

and therefore the criterion for mode $\mathbf{q}$ to be stable is

$$\kappa q^4 + \gamma q^2 + k > 0. \tag{29}$$

In the case of an infinite tissue all modes $q > 0$ are allowed, such that an infinite tissue is stable only for large enough tension γ:

$$\gamma > \gamma^* = -2\sqrt{\kappa k}. \tag{30}$$

The above criterion implies that a large tissue under compression ($\gamma < 0$) is stable only if the tissue is connected to the ECM with a nonzero attachment stiffness, $k \neq 0$.

Stability analysis of a clamped circular tissue region subjected to a contractile boundary

We consider here a stable tissue under tension $\gamma_0 > -2\sqrt{\kappa k}$. A circular region of the tissue is surrounded by a contractile boundary exerting a line tension Λ (Fig.5A, Fig.S4H). The resulting compression γ_c on the tissue circular region with radius R is then given by the law of Laplace:

$$\gamma_c = \gamma_0 - \frac{\Lambda}{R}. \tag{31}$$

For simplicity we consider that the circular region is clamped at its boundary. The dynamic equation for the shape of the tissue region is given by Eq. 26 with $\gamma = \gamma_c$. We assume rotational symmetry of the tissue region and denote r the radial coordinate. The clamped boundary conditions then read

$$h(R) = 0 \tag{32}$$

$$\partial_r h(R) = 0. \tag{33}$$

To study the stability of the circular tissue region, we make the following ansatz for $h(r, t)$:

$$h(r, t) = \tilde{h}(r) e^{st}. \tag{34}$$

We require that $Im(s) = 0$ and find that the tissue is unstable when a solution can be found for non-zero $\tilde{h}(r)$ and $s > 0$. Following this ansatz Eq. 26 can be rewritten

$$-\kappa\Delta_r^2\tilde{h}(r) + \gamma_c\Delta_r\tilde{h}(r) - (k + \alpha s)\tilde{h}(r) = 0, \tag{35}$$

where Δ_r is the Laplacian in polar coordinates with rotational symmetry

$$\Delta_r = \frac{\partial^2}{\partial r^2} + \frac{1}{r}\frac{\partial}{\partial r}. \tag{36}$$

The solution of Eq. 35 can be written

$$\tilde{h}(r, s) = AJ_0(\lambda_+ r) + BJ_0(\lambda_- r) + CY_0(\chi_+ r) + DY_0(\chi_- r), \tag{37}$$

where we have introduced 4 unknown constants A, B, C, D which have to be specified by the boundary conditions, and $J_0(x)$ and $Y_0(x)$ are Bessel functions of the first and second kind, respectively. Because $Y_0(x)$ diverges for $x \to 0$ and the height function must stay finite for $r \to 0$, it follows that $C = D = 0$. The two inverse length scales $\lambda_\pm > 0$ are solutions of the polynomial equation

$$\kappa\lambda_\pm^4 + \gamma_c\lambda_\pm^2 + (k + \alpha s) = 0, \tag{38}$$

which follows from the relation $\Delta_r J_0(\lambda r) = -\lambda^2 J_0(\lambda r)$. Solving Eq. 38, we find the following expressions for λ_+ and λ_-:

$$\lambda_\pm = \sqrt{-\frac{\gamma_c}{2\kappa} \pm \sqrt{\frac{\gamma_c^2}{4\kappa^2} - \frac{k + \alpha s}{\kappa}}}. \tag{39}$$

Imposing the boundary conditions (32)-(33) then results in the following two relations

$$AJ_0(\lambda_+ R) + BJ_0(\lambda_- R) = 0 \tag{40}$$

$$A\lambda_+ J_1(\lambda_+ R) + B\lambda_- J_1(\lambda_- R) = 0, \tag{41}$$

which have a non-zero solution for A and B provided that

$$F(s) = \lambda_- J_0(\lambda_+ R)J_1(\lambda_- R) - \lambda_+ J_0(\lambda_- R)J_1(\lambda_+ R) = 0. \tag{42}$$

where the dependency on s arises from λ_+ and λ_-. Solutions of Eq. 42 yields the rates of growth of perturbations of the shape of the circular region. Stability of the circular tissue region requires that all these solutions verify $Re(s) < 0$. The stability threshold of the clone can be found by solving the equation $F(0) = 0$. In doing so, a trivial solution $\lambda_+ = \lambda_-$ can be discarded as it results in a vanishing height profile. To obtain Figures S4K and S4L, we solve this equation numerically to obtain a phase diagram as a function of R and Λ.

We now give analytical expressions for the boundaries of the stability diagram in simple limits. We first note that in the limit of a large tissue region $R \to \infty$, the stability criterion converges to the stability threshold of

an infinite tissue, $\gamma_c > -2\sqrt{\kappa k}$ (Eq. 30). As a result, for large R the circular tissue region is unstable for

$$R < \frac{\Lambda}{\gamma_0 + 2\sqrt{k\kappa}}. \tag{43}$$

Subsequently we find the stability line in the limit where there is no elastic attachment to the ECM, $k = 0$. The stability threshold then reads simply

$$J_1\left(\sqrt{\frac{-\gamma_c}{\kappa}}R\right) = 0, \tag{44}$$

which has solution only for $\gamma_c < 0$, as the region is always stable for $\gamma_c > 0$. Denoting $j_{11} \simeq 3.832$ the first zero of the Bessel function J_1 and using Eq. 31, the condition for the circular region to be unstable can be rewritten

$$\gamma_0 R^2 - \Lambda R + j_{11}^2 \kappa < 0 \tag{45}$$

such that the sizes of unstable circular region are given for $k = 0$ by

$$\frac{\Lambda}{2\gamma_0} - \sqrt{\left(\frac{\Lambda}{2\gamma_0}\right)^2 - \frac{j_{11}^2\kappa}{\gamma_0}} < R < \frac{\Lambda}{2\gamma_0} + \sqrt{\left(\frac{\Lambda}{2\gamma_0}\right)^2 - \frac{j_{11}^2\kappa}{\gamma_0}} \qquad \text{for } \gamma_0 > 0 \tag{46}$$

$$R > \frac{j_{11}^2\kappa}{\Lambda} \qquad \text{for } \gamma_0 = 0 \tag{47}$$

$$R > \frac{\Lambda}{2\gamma_0} + \sqrt{\left(\frac{\Lambda}{2\gamma_0}\right)^2 - \frac{{j_{11}}^2\kappa}{\gamma_0}} \qquad \text{for } \gamma_0 < 0. \tag{48}$$

The lower limit for all values of γ_0 is $R > j_{11}^2\kappa/\Lambda$ for large $\Lambda \gg \sqrt{\kappa|\gamma_0|}$. An upper limit to stability exists only for positive tension $\gamma_0 > 0$; for negative tension $\gamma_0 < 0$ an infinite tissue is unstable, such that for $R \to \infty$ the circular region is always unstable when $k = 0$. The asymptotic stability criterion 43 indicates however that for $k > 0$ and $\gamma_0 < 0$, a region of large stable tissue sizes arises at large R when the attaching spring elasticity is large enough, $k > \gamma_0^2/(4\kappa)$.

Relation between the continuous model and the 3D Vertex Model

In this section the coarse grained parameters γ_0, κ, k and Λ of the continuum theory are related to the parameters of the 3D Vertex Model. The coarse grained tissue parameters are derived for a homogeneous tissue of identical cells arranged in a hexagonal packing. A similar approach is used in Ref. [S11]. We also do not discuss here anisotropic effects in the plane of the tissue. We take into account surface tensions on lateral, apical, and basal surfaces (T_l, T_a and T_b respectively), apical and basal line tensions (Λ_a, Λ_b respectively), elastic attachment of the vertices to the ECM (k_v) and the external tension acting on the tissue (T_{ext}). These parameters are taken here to be independent of the tissue geometry. The apical and basal areas of cell α are denoted A_α^a and A_α^b, the perimeters are denoted P_α^a and P_α^b, the volume is denoted V_α and the average Euclidean distance of the basal vertices to the ECM is denoted d_α. Following these assumptions, the mechanical energy W_Ω of a region of tissue Ω can be written as a sum of the effective free

energies of cells contained in this region of tissue

$$W_\Omega = \sum_{\alpha \in \Omega} \left(\frac{K_{3D}}{2}(V_\alpha - V^0)^2 + T^a A^a_\alpha + T^b A^b_\alpha + \frac{T^l}{2} A^l_\alpha + \frac{\Lambda^a}{2} P^a_\alpha + \frac{\Lambda^b}{2} P^b_\alpha + 2\frac{k_v}{2} d^2_\alpha \right). \tag{49}$$

where the factor 2 in the last term arises from the fact that each cell has on average 2 vertices. If all cells are assumed to have the same shape, the corresponding energy density $w = W_\Omega \rho / N_\Omega$ can be written:

$$w = \rho \left(\frac{K_{3D}}{2}(V - V^0)^2 + T^a A^a + T^b A^b + \frac{T^l}{2} A^l + \frac{\Lambda^a}{2} P^a + \frac{\Lambda^b}{2} P^b + k_v d^2 \right). \tag{50}$$

with N_Ω the number of cells within the piece of tissue Ω. We restrict ourselves here to cells with apical and basal surfaces being regular hexagons.The energy density $w(h, R_a, R_b)$ can then be reexpressed in terms of the cell height h and apical and basal radii R_a and R_b. The apical, basal and lateral surface areas, apical and basal perimeters and cell volume can indeed be related to h, R_a, R_b through the following relations:

$$A^a = \frac{3\sqrt{3}}{2} R_a^2 \tag{51}$$

$$A^b = \frac{3\sqrt{3}}{2} R_b^2 \tag{52}$$

$$A^l = 3\,(R_a + R_b) \sqrt{h^2 + \frac{3}{4}(R_a - R_b)^2} \tag{53}$$

$$P^a = 6R_a \tag{54}$$

$$P^b = 6R_b \tag{55}$$

$$V = \frac{\sqrt{3}}{2} h \left(R_a^2 + R_b^2 + R_a R_b \right). \tag{56}$$

We consider here the limit $K_{3D} \to \infty$ where the cell volume V is constrained to be equal to the reference volume V_0. The energy density $w(\rho, C, V_0)$ can then be written in terms of the cell density ρ, the total tissue curvature C and the reference volume V_0 through the following change of variables (Fig.S4I-J):

$$\rho = \frac{4}{(\sqrt{A^a} + \sqrt{A^b})^2} = \frac{8}{3\sqrt{3}(R_a + R_b)^2} \tag{57}$$

$$C = \frac{4(R_b - R_a)}{(R_a + R_b)h} \tag{58}$$

$$V_0 = \frac{\sqrt{3}}{2} h \left(R_a^2 + R_b^2 + R_a R_b \right). \tag{59}$$

For small curvature C, the energy density w can be expanded in power of the curvature C:

$$w(\rho, C, V_0) \simeq w_0(\rho, V_0) - \kappa C_0 C + \frac{\kappa}{2} C^2 + \frac{k}{2} d^2, \tag{60}$$

where the bending modulus of the tissue κ and the preferred curvature C_0 are functions of ρ, V_0 and the tissue line and surface tensions. In addition, we have intro cued the coarse-grained ECM elastic resistance $k = 2k_v\rho$. We obtain below the explicit expressions for these two mechanical quantities.

Bending modulus and preferred curvature

The effective bending modulus of the tissue κ is given by

$$\kappa = \left.\frac{\partial^2 w}{\partial C^2}\right|_{C=0} \tag{61}$$

$$= \frac{T^a + T^b}{8} V_0^2 \rho^2 + \frac{\sqrt{2}}{8 \cdot 3^{3/4}} T_l V_0 \sqrt{\rho} \left(\sqrt{3} - V_0^2 \rho^3\right) \tag{62}$$

and the preferred curvature of the tissue reads

$$C_0 = \frac{V_0\,\rho}{2\kappa} \left((T_a - T_b) + \frac{3^{1/4}}{\sqrt{2}} \sqrt{\rho} (\Lambda_a - \Lambda_b)\right). \tag{63}$$

Note that although κ can become negative for sufficiently large ρ, we have performed simulations here with parameters such that $\kappa > 0$.

Total in-plane surface tension

The effective surface tension γ exerted by the tissue depends on the apical and basal surface tensions, the apical and basal line tensions and the lateral surface tension:

$$\gamma = \frac{d(\rho^{-1} w_0)}{d(\rho^{-1})} \tag{64}$$

$$= T^a + T^b + \frac{3^{1/4}}{\sqrt{2}} \sqrt{\rho} (\Lambda^a + \Lambda^b) - \frac{3^{1/4}}{\sqrt{2}} \rho^{3/2} T_l V_0. \tag{65}$$

The effective surface tension exerted by the tissue has to be balanced by the external tension (i.e. $T_{\text{ext}} = \gamma$) for the tissue to be in mechanical equilibrium.
We rewrite the expression for the external tension by introducing the cell aspect ratio

$$\beta = \frac{h}{\sqrt{A}}. \tag{66}$$

With this definition, the total stress given in Eq. 65 can be rewritten

$$T_{\text{ext}} = \gamma_a(\beta) + \gamma_b(\beta) - \beta \frac{3^{1/4}}{\sqrt{2}} T_l, \tag{67}$$

where the total tensions exerted in the apical and the basal plane of the tissue γ_a and γ_b are respectively given by

$$\gamma_a(\beta) = T_a + \frac{3^{1/4}}{\sqrt{2}} \beta^{1/3} \frac{\Lambda_a}{l_0} \tag{68}$$

$$\gamma_b(\beta) = T_b + \frac{3^{1/4}}{\sqrt{2}} \beta^{1/3} \frac{\Lambda_b}{l_0}. \tag{69}$$

ECM elastic resistance

The coupling to the ECM in the 3D Vertex Model is represented by springs with spring constant k_v, that connect the basal vertices to the ECM. In a regular hexagonal packing every cell contains 6 vertices, which it each shares with two neighbouring cells, giving rise to the following coarse grained parameter of ECM attachment:

$$k = \frac{6}{3}\frac{k_v}{A_\alpha} = 2k_v\rho. \tag{70}$$

Clone boundary line tension

The excess tension due to the boundary effect around the clone can be due to an increase in apical and basal line tensions, as well as in lateral surface tension, Λ_a^e, Λ_b^e and T_l^e respectively, where the lateral surface tension has to be integrated over the cell height:

$$\Lambda = \Lambda_a^e + \Lambda_b^e + T_l^e V_0 \rho. \tag{71}$$

Critical buckling radii in the 3D Vertex Model

Using expressions for the coarse grained mechanical parameters as functions of the tensions generated in the 3D Vertex Model, an approximate critical buckling radii can be derived for simulations performed with the set of mechanical parameters given in Table 1. This can be done by using the stability criterion 42 obtained with the continuum theory and rewriting the continuum theory parameters in terms of the 3D Vertex Model parameters.

In order to relate the simulations and the analytical calculations, we also have to estimate the number of cells N in a circular clone with given radius R:

$$N = R^2\pi\rho. \tag{72}$$

Then we can use the experimental value of the aspect ratio of cells $\beta = h\sqrt{\rho} \approx 16.2$, to estimate the 2D cell density ρ for a given fixed cellular volume V_0:

$$\rho = \left(\frac{\beta}{V_0}\right)^{2/3}, \tag{73}$$

and using the relations 62, 65, 70 and 71, the values of the normalised effective mechanical parameters in simulations can be obtained:

$$\kappa/(T_l l_0^2) \simeq 0.34 \tag{74}$$

$$\gamma_0/T_l \simeq -4.2 \tag{75}$$

$$k/\left(\frac{T_l}{l_0^2}\right) \simeq 64.0 \tag{76}$$

$$\Lambda/(\beta^{2/3} l_0\, T_l + \Lambda_a) \simeq 2.0 \tag{77}$$

These normalised parameter values are used in Supplementary Figure S4L to plot phase spaces of predicted buckling instabilities for the simulations, assuming clamped boundaries of the clone.

Using the stability criterion given in Eq. 42, we show in Fig. S4L a phase diagram of buckling instability for a clone clamped at its boundary, with the resulting coarse grained parameters obtained above. For the increase in line tension corresponding to a 3-fold increase in apical line tension and lateral surface tension, the predicted range of buckling lies between about 3 and 106 cells. The maximum value of cyst formation is in qualitative agreement with the maximum size of observed cysts. Note however that the clamped boundary conditions used in this calculation are different from 3D Vertex Model simulations where the clone is embedded in the tissue.

References

[S1] Brandon D Bunker et al. ``The transcriptional response to tumorigenic polarity loss in Drosophila''. In: *eLife* 4 (2015), e03189.

[S2] Anne-Kathrin Classen et al. ``A tumor suppressor activity of Drosophila Polycomb genes mediated by JAK-STAT signaling.'' In: *Nature genetics* 41.10 (2009), pp. 1150–1155. ISSN: 1061-4036. DOI: 10.1038/ng.445. URL: http://dx.doi.org/10.1038/ng.445.

[S3] Reza Farhadifar et al. ``The influence of cell mechanics, cell-cell interactions, and proliferation on epithelial packing.'' In: *Current biology : CB* 17.24 (Dec. 2007), pp. 2095–104. ISSN: 0960-9822. DOI: 10.1016/j.cub.2007.11.049. URL: http://www.ncbi.nlm.nih.gov/pubmed/18082406.

[S4] Guillaume Salbreux, Guillaume Charras, and Ewa Paluch. ``Actin cortex mechanics and cellular morphogenesis''. In: *Trends in Cell Biology* 22.10 (2012), pp. 536–545. ISSN: 09628924. DOI: 10.1016/j.tcb.2012.07.001. arXiv: 84866882608. URL: http://dx.doi.org/10.1016/j.tcb.2012.07.001.

[S5] William H Press. *Numerical recipes 3rd edition: The art of scientific computing*. Cambridge university press, 2007.

[S6] W Helfrich. ``Elastic properties of lipid bilayers: theory and possible experiments.'' In: *Zeitschrift fur Naturforschung. Teil C: Biochemie, Biophysik, Biologie, Virologie* 28.11 (1973), pp. 693–703. ISSN: 0341-0471. DOI: 10.1002/mus.880040211.

[S7] Reinhard Lipowsky. ``The conformation of membranes.'' In: *Nature* 349 (1991). ISSN: 0028-0836. DOI: 10.1038/349475a0.

[S8] Reinhard Lipowsky. ``Budding of membranes induced by intramembrane domains''. In: *Journal de Physique II* 2.10 (1992), pp. 1825–1840. ISSN: 1155-4312. DOI: 10.1051/jp2:1992238.

[S9] Stephen Timoshenko and Serge Woinowksy-Krieger. *Theory of Plates and Shells*. McGraw-Hill Book Company, 1987.

[S10] Stephen P Timoshenko and James M Gere. *Theory of elastic stability, Engineering societies monographs*. 1961.

[S11] Edouard Hannezo, Jacques Prost, and Jean-Francois Joanny. ``Theory of epithelial sheet morphology in three dimensions.'' In: *Proceedings of the National Academy of Sciences of the United States of America* 111.1 (Jan. 2014), pp. 27–32. ISSN: 1091-6490. DOI: 10.1073/pnas.1312076111. URL: http://www.ncbi.nlm.nih.gov/pubmed/24367079.
